# Robust Inference of Genetic Exchange Communities from Microbial Genomes Using TF-IDF

**DOI:** 10.3389/fmicb.2017.00021

**Published:** 2017-01-19

**Authors:** Yingnan Cong, Yao-ban Chan, Charles A. Phillips, Michael A. Langston, Mark A. Ragan

**Affiliations:** ^1^Institute for Molecular Bioscience and ARC Centre of Excellence in Bioinformatics, University of Queensland, St LuciaQLD, Australia; ^2^School of Mathematics and Statistics, University of Melbourne, ParkvilleVIC, Australia; ^3^Department of Electrical Engineering and Computer Science, University of Tennessee, KnoxvilleTN, USA

**Keywords:** TF-IDF, lateral genetic transfer, horizontal genetic transfer, microbial genomes, genetic exchange community, lateral genetic transfer network, clique analysis

## Abstract

Bacteria and archaea can exchange genetic material across lineages through processes of lateral genetic transfer (LGT). Collectively, these exchange relationships can be modeled as a network and analyzed using concepts from graph theory. In particular, densely connected regions within an LGT network have been defined as genetic exchange communities (GECs). However, it has been problematic to construct networks in which edges solely represent LGT. Here we apply term frequency-inverse document frequency (TF-IDF), an alignment-free method originating from document analysis, to infer regions of lateral origin in bacterial genomes. We examine four empirical datasets of different size (number of genomes) and phyletic breadth, varying a key parameter (word length *k*) within bounds established in previous work. We map the inferred lateral regions to genes in recipient genomes, and construct networks in which the nodes are groups of genomes, and the edges natively represent LGT. We then extract maximum and maximal cliques (i.e., GECs) from these graphs, and identify nodes that belong to GECs across a wide range of *k*. Most surviving lateral transfer has happened within these GECs. Using Gene Ontology enrichment tests we demonstrate that biological processes associated with metabolism, regulation and transport are often over-represented among the genes affected by LGT within these communities. These enrichments are largely robust to change of *k*.

## Introduction

Bacteria and archaea (BA) comprise much of the planet’s biodiversity. Although individually inconspicuous, communities of these organisms are responsible for key biological and geochemical processes including nitrogen fixation, aerobic and anaerobic digestion of biomass, and oxidative dissolution of minerals. Bacteria also cause a range of diseases in plants, animals, and humans. Since 1996, genome-sequencing technologies have been applied initially to study bacterial pathogenesis, and more recently to understand environmental processes and explore biodiversity. Genome sequences are publicly available for more than 30,000 BA, and large international projects are underway to sequence many thousands more.

Arguably, the two most-notable discoveries from the first two decades of microbial genomics have been the extent of strain-to-strain variation in gene content (Tettelin et al., 2005; Segerman, 2012; Croucher et al., 2014), and the prevalence of lateral genetic transfer (LGT). It has long been known that bacteria can take up genetic material from their surroundings, incorporate it into their main genome (or maintain it on extrachromosomal elements) and transmit it to subsequent generations. More than 35 years ago, unexpected patterns of gene presence among bacterial taxa and anomalous topologies of phylogenetic trees inferred for bacterial proteins were attributed, somewhat controversially, to LGT (Ambler et al., 1979a,b; Dickerson, 1980; Woese et al., 1980). In the last 10–15 years, large-scale analysis has revealed the surprising extent of LGT among BA, with many estimates indicating that 10–40% of genes may have a relatively recent lateral origin; for details see the review by Ragan and Beiko (2009). Thus while all organisms transmit genetic information vertically from parent to offspring, BA simultaneously operate an orthogonal genetics that links important components of their genomes with viruses, phage, plasmids and free environmental DNA in a vast web (Doolittle, 1999; Bryant and Moulton, 2004; Beiko et al., 2005; Kunin et al., 2005; Dagan et al., 2008; Dagan and Martin, 2009; Halary et al., 2010; Puigbò et al., 2010; Bapteste et al., 2013; Koonin, 2015).

We and others (Beiko et al., 2005; Dagan et al., 2008; Dagan and Martin, 2009; Popa et al., 2011) have sought to model this web of genetic relationships as a graph in which vertices represent observed entities that carry DNA (genomes, and in some applications also plasmids and phage), and edges represent the inferred transmission of genetic material between them. However, resolving the lateral signal turns out to be unexpectedly tricky. Two genomes that have descended only recently from a common ancestor are unlikely to differ greatly in genome sequence or gene content, and if they are accorded individual vertices, the similarity between them will arise almost entirely from vertical signal. To the extent that our graph is intended to help us understand patterns of LGT, it makes sense to combine such genomes into a single vertex (node). As genomes diversify through time, it becomes increasingly desirable to represent them as separate vertices, because doing so potentially increases the resolution at which LGT can be studied; but pairwise edges represent a mixture of vertical and lateral signal. Moreover, older LGT (more-basal in the tree of vertical signal) becomes established in lineages and begins to be allocated among present-day genomes in hierarchical patterns that reinforce local vertical signal (Gogarten et al., 2002; Gogarten and Townsend, 2005). Thus by flattening the temporal (historical) dimension into the plane of the (present-day) graph, we hide sequence diversity in the vertices and admix vertical and lateral signal in the edges. Although an optimal balance (or multiple locally optimal balances across the tree) can be sought, these issues remain.

Until now, the nature of the edges has received the most attention. LGT detection methods can be classified into two general types: surrogate and phylogenetic (Ragan, 2001a,b; Gogarten and Townsend, 2005). The former include methods based on all-versus-all sequence comparison (Bansal et al., 1998; Lima-Mendez et al., 2008; Fondi and Fani, 2010; Halary et al., 2010) or reciprocal best matches (Tatusov et al., 1997; Bork et al., 1998) of genes or proteins. Some additional filter must then be applied to distinguish matches that are unexpectedly strong after correction for shared vertical relationship (and perhaps other factors, e.g., functional constraints), and therefore candidates for LGT. This filter might involve a more-stringent match threshold (Halary et al., 2010) and/or subtracting edges present in a trusted reference tree (Dagan et al., 2008; Dagan and Martin, 2009). A converse strategy was employed by Clarke et al. (2002), who were interested only in the vertical component. Alternatively in the phylogenetic approach, a test tree (inferred for a putatively orthologous gene or protein family) is compared with a reference (genome or organismal) tree, and instances of topological incongruence that meet a statistical support criterion are considered *prima facie* cases of LGT (Goldman et al., 2000; Beiko et al., 2005; Zhaxybayeva et al., 2006; Beiko and Ragan, 2008). Even so, reconstructing the pathway of inferred LGT as shortest edit paths is computationally hard and may not yield a unique solution, or any solution at all (Beiko and Hamilton, 2006). Popa et al. (2011) employ a hybrid approach in which only genes assessed as having regions of anomalous G+C content are input into phylogenetic discordance analysis.

Several objections have been raised to these approaches, both individually and collectively. We have repeatedly argued that as genes are not the actual units of LGT, gene families should not be the primary units of analysis (Chan et al., 2009a,b). Doolittle and Bapteste (2007) and Doolittle (2009) have argued that by using a reference tree external to the analysis, we impose a higher standard of evidence on rejecting the reference topology (and thereby inferring LGT) than on accepting (or failing to reject) it, thereby according the vertical paradigm a methodologically unfair and theoretically unjustified advantage; for a conflicting opinion see O’Malley and Koonin (2011). A way is needed to infer LGT directly, positively and fairly in large genome-scale datasets.

Recently we (Cong et al., 2016a,b) introduced term frequency-inverse document frequency (TF-IDF) as an accurate, scalable approach to infer LGT among microbial genomes. Using TF-IDF, edges represent only lateral signal and can be inferred directly from whole genomes without first parsing them into individual genes. These edges are directional: transfers are inferred from a group of donor genomes to a single recipient genome. No comparison with an external topology is required, although inference quality may be improved if the group structure reflects phylogeny (Cong et al., 2016b).

Direct access to edges that represent only the lateral component of genetic relationships greatly simplifies the interpretation of such graphs: they are natively LGT networks. Skippington and Ragan (2011) defined a genetic exchange community (GEC) as a densely connected region of an LGT network. Recognizing the limitations of then-existing methods and data, these authors operationally defined a GEC as “a set of entities, each of which has over time both donated genetic material to, and received genetic material from, every other entity in that GEC, via a path of lateral transfer.” These GECs do not exist *a priori* in nature, but rather are “actively fashioned (and continually refashioned) by the complex ongoing interplay among habitats, donors, vectors, recipients, mechanisms, sequences, population structures and selection” (Skippington and Ragan, 2011). Biological problems that could be modeled as involving dense edge sets in LGT graphs include the number, size, geospatial extent, taxonomic or habitat diversity of GECs in the microbial biosphere, and the role of vectors in mediating the exchange of pathogenicity, virulence or resistance factors among pathogens, primary hosts and secondary hosts (Halary et al., 2010; Popa et al., 2011; Skippington and Ragan, 2011, 2012).

Skippington and Ragan (2011) further proposed that dense regions in LGT graphs might be described using concepts from graph theory, including cliques (complete subgraphs, i.e., groups of nodes that are all connected directly to each other), paracliques (cliques missing a few edges: Chesler and Langston, 2007; Hagan et al., 2016), other forms of near-cliques, or looser structures such as transitively closed sets, cycles, paths or walks. They were not, however, in a position to recommend one of these notions over the others. Our previous results make it clear that edges, hence dense edge sets in LGT graphs and their biological interpretations, can be sensitive to the choice of TF-IDF parameters. Notably, precision and recall can be sensitive to the size of *k* (Cong et al., 2016a), and edges to the structure and delineation of groups (Cong et al., 2016b). It may be that different values of *k* are more sensitive to different assumptions or biological processes; because of this, we are interested in inferences of GECs that are robust to change of *k*. In the present work, three empirical genome-scale datasets we studied in detail earlier (Cong et al., 2016b) provide a solid foundation for addressing these issues. We add a fourth dataset to control further for balance across taxa, while removing a few poorly represented and/or anomalous taxa; and present the alignment-free LGT network analytical workflow end-to-end, including extraction of maximum and maximal cliques.

Specifically, here we examine (a) whether and how *k* affects cliques in LGT networks; (b) whether *core nodes*, stable to variation of *k* within biologically reasonable bounds, exist in different cliques; and (c) whether and how our biological process (functional) interpretation is consequently affected. More broadly, we believe that the approach pioneered here will provide a framework for understanding the extent and biological significance of LGT in complex environments.

## Materials and Methods

### Datasets and Groups

Here we analyze four datasets, three of which we introduced earlier (Cong et al., 2016b): 20 *Escherichia coli* and seven *Shigella* genomes (ECS dataset), 110 enteric bacterial genomes (EB) and 143 genomes from BA. To these we now add a dataset of 144 bacterial genomes (BAC) purpose-built for this analysis. When this latter dataset was constructed, 24 bacterial orders in 12 classes were represented by at least one genus from which at least six genomes had been sequenced to high quality. Within each of these orders we selected one genus at random, and if that genus was represented by more than six genomes we chose six of them at random, thereby constituting BAC with 144 genomes in 24 genera. In this way we attempt to achieve as broad and balanced selection of genomes across Bacteria as possible, given the available data, a synthetic classification (NCBI) and the underlying biology.

As noted above, TF-IDF infers transfers from identified groups of donor genomes into a single recipient genome. It is therefore necessary to delineate groups prior to analysis. Here we recognize groups within the ECS dataset according to multi-locus sequence type (MLST; Gordon et al., 2008); within EB by genus, sometimes combining *Escherichia* and *Shigella* genomes into a single group; within BA by phylum, or alternatively by class; and within BAC by order. Other approaches to grouping are possible, some of which we explored earlier (Cong et al., 2016b).

### Inference of Lateral Segments Using TF-IDF

The TF-IDF method *per se* proceeds in four steps, as follows: for each dataset we (A) extract unique *k*-mers and construct a *k*-mer dictionary; and (B) build a relationship matrix *R* in which rows represent individual genomes, columns represent the identified groups of genomes, and elements count the number of identical *k*-mers present in a genome *and* in each group other than its own. These counts are then normalized, and the mean element value computed over *R*. Unless indicated otherwise, this mean is used as the threshold for recognizing that a genome in the dataset may contain *k*-mers donated by a group in the dataset. (C) Within each genome, we then construct segments from neighboring *k*-mers that are present in the same donor group. We further merge these segments if they are separated by less than a gap threshold *G*, yielding *potential lateral segments*. (D) If the average frequency of *k*-mers in a potential lateral segment is less than that of all *k*-mers in the target genome’s own group, then we consider it an *inferred lateral segment*. Step B implements the IDF component, and step D the TF component (Cong et al., 2016a,b). Pseudocode is available in the Supplementary Material to Cong et al. (2016a), and the TF-IDF source code at https://github.com/congyingnan/TF-IDF.git.

Because the TF component requires a potential lateral segment to be infrequent in genomes of its own group, TF-IDF is expected to identify recent LGT events, i.e., those affecting one or a very few genomes in a target group. By contrast, *k*-mers descendant from transfers more ancient than the common ancestor of a target group would tend to occur widely within that group, and thus fail to be inferred as lateral.

### Mapping Inferred Lateral Regions to Genes

We consider a gene to be lateral if it contains, or is overlapped by, at least one inferred lateral segment such that two distinct length thresholds are met. The inferred lateral segment must itself contain at least a specified minimum number of *k*-mers (including *k*-mers in any intervening gaps up to *G* = 2*k*); this minimum number is 10 for the BA dataset, 100 for EB, 500 for ECS (Cong et al., 2016b) and 10 for BAC. These values approximate the average length of all LGT detections in each dataset, thereby controlling in part for differences in sequence diversity among the datasets. In addition, the overlap must extend for at least a specified minimum number of *k*-mers (again including *k*-mers in gaps up to *G* = 2*k*); this minimum number is 10 for BA, 100 for ECS and EB (Cong et al., 2016b) and 10 for BAC.

### End-to-End Workflow: Overview

As introduced above, GECs might variously be described as *paths, transitively closed sets, paracliques* or *cliques* (Skippington and Ragan, 2011). The first two structures fail to capture the density of connectivity, and many such structures of nearly equivalent size or value can often be found in relatively highly connected graphs such as the LGT networks we derive above. Paracliques differ from the corresponding cliques by relaxing the strict requirement that all edges be present, and in this way might better ameliorate the effects of incomplete or imperfect data. In the absence of theory or established practice, paraclique parameters would have to be optimized for each dataset, requiring intense computation. Constrained by these considerations, here we adopt the strictest yet clearest definition of GEC, as *a set of vertices that share (donate or receive) genetic material from all other nodes within this set*. That is, there must be at least one direct path between each node and every other node. Using this definition, GECs correspond to cliques in the LGT network.

The discovery and analysis of such cliques proceeds in four main steps:

(a) construct LGT networks based on the results of TF-IDF;
(b) consolidate these networks by collapsing recipient genomes to recipient groups;
(c) extract maximum and maximal cliques from the LGT network; and
(d) perform enrichment tests on biological processes underlying the cliques.

### Construction of LGT Networks

From our previous work (Cong et al., 2016a,b) we know that *k* can strongly affect the detection of LGT, hence potentially the topologies of LGT networks. For that reason, we explore different values of *k* to test the stability of clique topology. In step (*a*) we explore values of *k* from 20 to 40. Depending on the data, false positives can predominate at *k* ≤ 20, while at *k* ≥ 40 shared *k*-mers become too rare, resulting in diminished performance. For consistency with earlier studies on the ECS, EB and BA datasets (Cong et al., 2016b), gap size *G* was fixed at 2*k*. The step size is 10 for the ECS and EB datasets, while for BA (where LGT signal is much weaker) and BAC (not studied heretofore) we set step size as 5 for improved resolution against *k*.

### Consolidation of the LGT Network Graph

**FIGURE 1:**
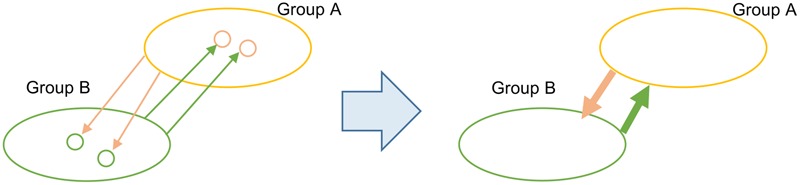
**Merger of LGT relationships from group-to-sequence into group-to-group**.

Our TF-IDF procedure infers LGT from a donor group to a recipient sequence, so at this point the vertices in our inferred networks are of two types: individual genomes when they are recipients of LGT, and groups of genomes when they are donors. Of course, members of a group may individually be (and often are) recipients of LGT from outside that group. Edges are directional, so we depict them using an arrow from donor to recipient (**Figure 1**).

We aim to delineate GECs, introduced above as sets of nodes that have both donated genetic material to, and received genetic material from, each other. However, it is unclear how to extract these relationships when nodes are of two types, individual sequences and groups. For this reason we take only groups as nodes in our network analysis. In step (*b*) we subsume each genome into its respective own-group identity, and merge all directed edges into those sequences into a single directed edge from the donor group to the recipient group (**Figure 1**). The integer weight on each edge gives the total number of genes inferred in this way to have been affected by LGT from donor to recipient groups.

### Extraction of Maximum and Maximal Cliques

Extracting cliques [step (*c*)] is known to be NP-hard (Karp, 1972), although a parameterized complexity approach (Abu-Khzam et al., 2006) is possible whenever the clique size can be bounded (Downey et al., 1999). For this we used the Graph Algorithms Pipeline for Pathway Analysis (GrAPPA) software suite (Langston Lab, the University of Tennessee^1^). GrAPPA integrates multiple graph-theoretical tools for biological data analysis, including those designed to find cliques and paracliques. It implements tools to extract patterns efficiently from graphs, but deals only with undirected and unweighted networks. For this reason, we reformulated our directed networks as undirected graphs (i.e., we disregarded the arrowhead), deleted all weight annotations (number of inferred LGT genes) on each edge, and merged edges between pairs of nodes that are both donors and recipients. Such reformulation does not make full use of the LGT information (e.g., directionality) provided by TF-IDF, but nonetheless it preserves connectivity information sufficient for discovery of GECs as currently defined.

Here we use GrAPPA to find, for each dataset, one *maximum clique* (a clique with the greatest number of vertices) and all *maximal cliques* (cliques that are not included in a larger clique). We report only those maximal cliques with at least three vertices.

### Gene Ontology Enrichment Tests

In step (*d*) we determine the biological processes that are enriched in the cliques previously extracted. As we show below (Results), the cliques inferred for the ECS and EB datasets encompass almost all the respective lateral genes, so the biological process enrichments are essentially the same as described previously (Cong et al., 2016b). Most LGT inferred for BAC involves the EB genera. Thus, here we report biological process enrichment only for cliques inferred, at different values of *k*, for the BA dataset. These genes are extracted from the GenBank genome record using GI numbers and coordinates, and collected as a test set. All genes in the dataset form the respective reference set. The enrichment statistic is a Fisher’s exact test, for which we set false discovery rate FDR = 0.05 as the threshold for selecting over- and under-represented Gene Ontology (Ashburner et al., 2000; Gene Ontology Consortium, 2004) terms.

## Results

Detailed results including LGT networks, maximum and maximal cliques, gene lists, NCBI accession numbers for all genome sequences, and group composition are available as Supplementary Material (Supplementary Figures 1–19, and Supplementary Tables 1–34). Very large or detailed Supplementary Figures are also available for download in high resolution at http://bioinformatics.org.au/tools-data/ under the category “Other.”

### ECS Dataset

We divide the ECS dataset (20 *Escherichia coli* and seven *Shigella* genomes) into six groups according to MLST (Gordon et al., 2008). In an earlier analysis of this dataset (Skippington and Ragan, 2012), lateral events identified by topological incongruence between trees inferred from individual putative orthogroups and an MRP (Ragan, 1992) reference supertree were shown to be biased more by phylogeny than by environment or lifestyle; concern was also expressed that defining GECs as cliques or paracliques might be too rigorous a standard.

**FIGURE 2:**
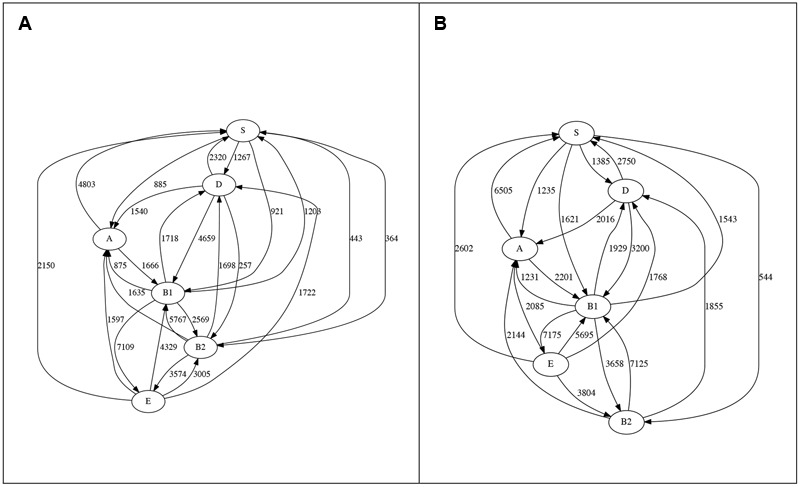
**LGT networks for ECS at (A)** *k* = 20 and **(B)** *k* = 30. At *k* = 40 connectivity is the same as in **(B)**, although values on the edges are usually larger.

Here, we use our TF-IDF method to infer LGT networks (**Figure 2**). For all *k* examined here, all six phyletic groups belong to a single clique, so the whole dataset forms one large GEC. Indeed, at *k* = 30 or 40, topologies of the two networks are identical (as before: Cong et al., 2016b). There is a clear trend overall of more detections on each edge as *k* increases, but with some exceptions: at *k* = 20 we find three edges not seen at *k* = 30, from group D to B2 (257 transfers), from B2 to S (443) and from B2 to E (3574), while transfers from D to B1 decrease from 4659 at *k* = 20 to 3200 at *k* = 30. For all other edges, more genes are affected by LGT at *k* = 30 than at *k* = 20. Likewise, when *k* is increased from 30 to 40, three edges show fewer detections (D to B1, 3200 to 1842; B1 to B2, 3658 to 3563; E to B2, 3804 to 3363) but all others have more.

**FIGURE 3:**
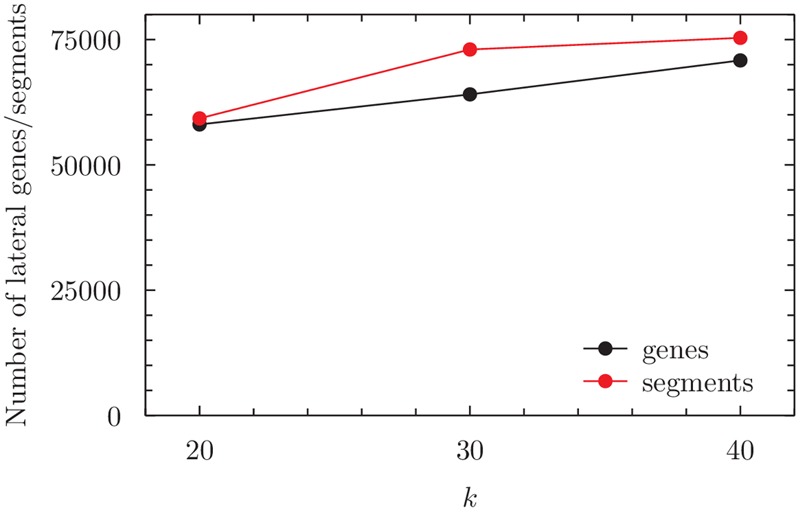
**Total number of lateral segments of length ≥500 *k*-mers (i.e., not only those mapping to genes), and total number of lateral genes, within the (maximum) clique inferred for the ECS dataset, as a function of *k*.** For numerical values see Supplementary Table 32.

Although clique topology is stable for 20 ≤*k* ≤ 40, the total number of lateral genes underlying each clique increases with *k* (**Figure 3**). This increase might appear to contradict our earlier finding that when *k* increases, the total number of detections and detection length should remain the same or decrease (at *G* = 2*k*). However, when *k* is small, more short segments tend to be detected as lateral (Supplementary Table 1). For example, at *k* = 20, 26% of lateral segments are ≥500 *k*-mers in length, our threshold for selecting the segments for mapping to genes. This proportion increases to 31% at *k* = 40. Thus, we infer more lateral segments of ≥500 *k*-mers at *k* = 40, which leads to more genes being inferred as affected by LGT.

### EB Dataset

The enteric bacteria dataset contains 110 genome sequences from five genera: *Escherichia, Shigella, Salmonella, Klebsiella* and *Yersinia*. Because the delineation of groups affects the detection results (Cong et al., 2016b), we infer LGT networks and extract cliques from five variants of this dataset: all genera present (referred to as EB-1); all genera except *Shigella* (EB-2) or alternatively, all except *Escherichia* (EB-3); with *Escherichia* and *Shigella* combined into a single group (EB-4); and with both *Escherichia* and *Shigella* removed (EB-5). These are five of the six variants we examined earlier (Cong et al., 2016b).

If we keep all 110 sequences and group them by genus (EB-1 dataset), the LGT network topologies change as *k* steps from 20 to 30 to 40. At *k* = 20, *Escherichia, Shigella, Klebsiella* constitute a single clique. At *k* = 30, we find two cliques, one consisting of *Escherichia* and *Shigella*, the other of *Escherichia* and *Klebsiella*. At *k* = 40 only one clique is found, consisting of *Escherichia* and *Shigella*. We infer many more LGT events between *Escherichia* and *Shigella* than between any other pair of genera. As *Escherichia* and *Shigella* are present in the clique across the examined range of *k*, we can say that they are the *core nodes* of this GEC.

Because genomes from *Escherichia* and *Shigella* share many more identical *k*-mers than do other groups, the lateral signal between these genera can drown out weaker lateral signal from or between other genera. This happens because the IDF values (elements of the *R* matrix) for these genomes are much higher than for the others (Cong et al., 2016a). This pushes up the IDF threshold, with the consequence that few lateral events are detected involving the other genera. To explore this effect, we also analyzed variant datasets which are modified so that *Escherichia* and *Shigella* do not both appear in the dataset as separate genera.

We first removed the *Shigella* genomes from the dataset while retaining those from *Escherichia*, thereby eliminating the effect of *Shigella* (EB-2 dataset). We now infer additional lateral events in both directions between all pairs of *Escherichia, Salmonella*, and *Klebsiella*. Thus we find a GEC composed of *Escherichia, Klebsiella* and *Salmonella* that remains stable with respect to *k*. We find similar results when we instead retain *Shigella* sequences while removing those of *Escherichia* (EB-3 dataset); the GEC here is *Shigella, Klebsiella* and *Salmonella.* We do infer lateral events between *Klebsiella* and *Yersinia* in EB-3, but these are not sufficient for *Yersinia* to join the GEC. In EB-4 we combine *Escherichia* and *Shigella* into a single group (ES); more lateral events were inferred from *Salmonella* to ES, but the GEC membership remains ES, *Salmonella* and *Klebsiella*. Lastly, to eliminate completely the effects of *Escherichia* and *Shigella* on LGT inference, we use only *Klebsiella, Salmonella* and *Yersinia* as input (EB-5). At *k* = 20 the sole clique contains all three genera, but at *k* = 30 or 40 the previous clique is split into two, one containing *Klebsiella* and *Salmonella* and the other *Klebsiella* and *Yersinia*. We thus conclude that *Escherichia, Shigella, Klebsiella* and *Salmonella* are all members of a larger GEC. Details are provided in **Table 1**.

**Table 1:** Lateral genes and cliques inferred for variants of the EB dataset at *k* = 20, 30, or 40.

Dataset	*k* size	Nodes in clique	Number of lateral genes in cliques	Number of lateral genes in network	Proportion (%)
EB-1	20	*Escherichia, Shigella, Klebsiella*	29527	29527	100%
	30	*Escherichia, Shigella*	29258	29264	99.9%
	30	*Escherichia, Klebsiella*	6	29264	0.1%
	40	*Escherichia, Shigella*	16968	16968	100%
EB-2	20	*Escherichia, Klebsiella, Salmonella*	23964	23970	99.9%
	30	*Escherichia, Klebsiella, Salmonella*	10840	10840	100%
	40	*Escherichia, Klebsiella, Salmonella*	7420	7426	99.9%
EB-3	20	*Klebsiella, Salmonella, Shigella*	15290	15290	100%
	30	*Klebsiella, Salmonella, Shigella*	6473	6501	99.5%
	40	*Klebsiella, Salmonella, Shigella*	3869	3909	98.9%
EB-4	20	*ES, Klebsiella, Salmonella*	24806	24811	99.9%
	30	*ES, Klebsiella, Salmonella*	10762	10762	100%
	40	*ES, Klebsiella, Salmonella*	7951	7952	99.9%
EB-5	20	*Klebsiella, Salmonella, Yersinia*	6721	6721	100%
	30	*Klebsiella, Yersinia*	123	2586	4.8%
	30	*Klebsiella, Salmonella*	2463	2586	95.2%
	40	*Klebsiella, Yersinia*	140	1559	9%
	40	*Klebsiella, Salmonella*	1419	1559	91%

### BA Dataset

The BA dataset, 143 genome sequences across BA, has been studied in our group using classical alignment-based and other computational methods for more than a decade (Beiko et al., 2005; Chan et al., 2009a,b). Like many empirical datasets it is unbalanced, with many more genomes representing some taxa (e.g., Proteobacteria, Firmicutes) than others. We group these genomes into fifteen phyla or, alternatively, into 31 classes. With more nodes than in the two previous datasets, there is potential for inferred LGT networks to be more complex. On the other hand, these genomes are more dissimilar to each other (Cong et al., 2016b), so fewer *k*-mers are shared and fewer instances of LGT are inferred.

**FIGURE 4:**
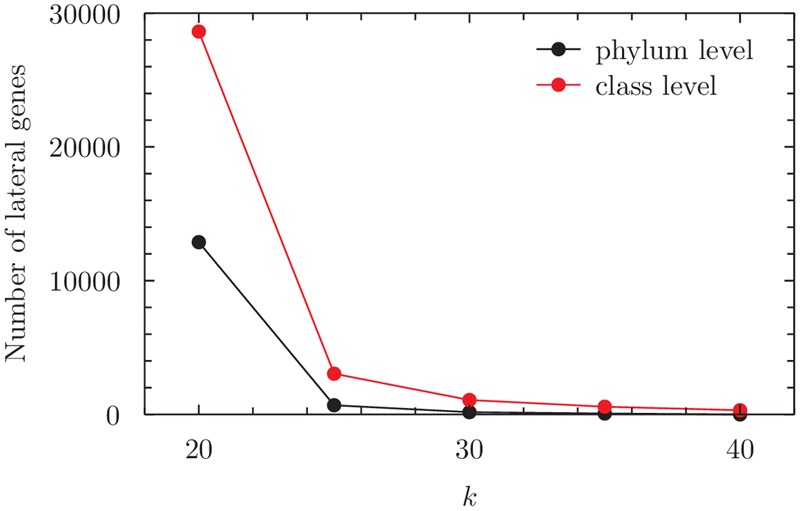
**Number of inferred lateral genes in the BA dataset at 20 ≤ *k* ≤ 40, analyzed at the level of phylum or of class.** For numerical values see Supplementary Table 33.

When groups are delineated by phylum, the number of total LGT detections decreases significantly as *k* increases (**Figure 4**), and this causes edges in the LGT network to vanish and the cliques to shrink. At the smallest value of *k* = 20 six maximal cliques are found, each with five phyla. Five of these contain the High G+C Firmicutes, Proteobacteria and Low G+C Firmicutes, which together represent 14797 lateral genes, 95.5% of the total inferred over the entire network. Thus these phyla form the core of the inter-phylum GEC. We also observe a smaller GEC of Nanoarchaeota, Euryarchaeota and Crenarchaeota; although based on only 10 lateral genes, it is notable for showing potential GECs among Archaea. In addition, the *Thermus/Deinococcus* phylum contributes 244 lateral events, 1.5% of the total; as our dataset contains only one strain in this phylum, this particular genome appears to be more LGT-active than many other bacterial genomes.

The number of detections drops sharply at *k* > 20; recall that our earlier simulations (Cong et al., 2016a) indicate potential false positives at *k* ≤ 20, presumably due to identical *k*-mers shared between sequences and groups simply by coincidence. As *k* increases and LGT detections decrease in number, some edges in the LGT network vanish, but the core nodes – the High G+C Firmicutes, Low G+C Firmicutes and Proteobacteria – remain as members of the maximal cliques. *Thermus*/*Deinococcus* also remains active in sharing LGT with Proteobacteria for all investigated *k*.

When these genomes are alternatively grouped by class, the LGT networks are more complex. Again we see a sharp drop in detections for *k* ≥ 20. At *k* = 20, all but one of the 31 classes are involved in LGT (30696 genes), and we observe 23 maximal cliques (≥3 nodes) in the LGT network; however, five classes form core members of the GEC, with each being present in 17 maximal cliques (≥5 nodes) and in the maximum clique. These classes are the Actinomycetales (5377 genes with lateral origin), *Bacillus*/*Clostridium* (2277) and the α- (5944), β- (7322) and γ-Proteobacteria (8596). Together they contain 77.7% of all genes that contain regions of inferred lateral origin.

Since the sequences within BA are relatively dissimilar from each other, many fewer *k*-mers are shared between sequences than in the ECS and EB datasets. Thus the LGT detections are very sensitive to *k* (**Figure 4**). At *k* = 30 the γ- and β-Proteobacteria, Actinomycetales and *Bacillus*/*Clostridium* are hubs and play key roles in most cliques; at *k* = 40 fewer genes are inferred as lateral, and only the former two classes remain as the core.

*Deinococcus* is inferred to exchange genetic material with β- and γ-Proteobacteria at 20 ≤*k* ≤ 40. Lateral events are also inferred between *Deinococcus* and Actinomycetales, and between *Deinococcus* and Chlorococcales, at *k* < 40.

### BAC Dataset

**FIGURE 5:**
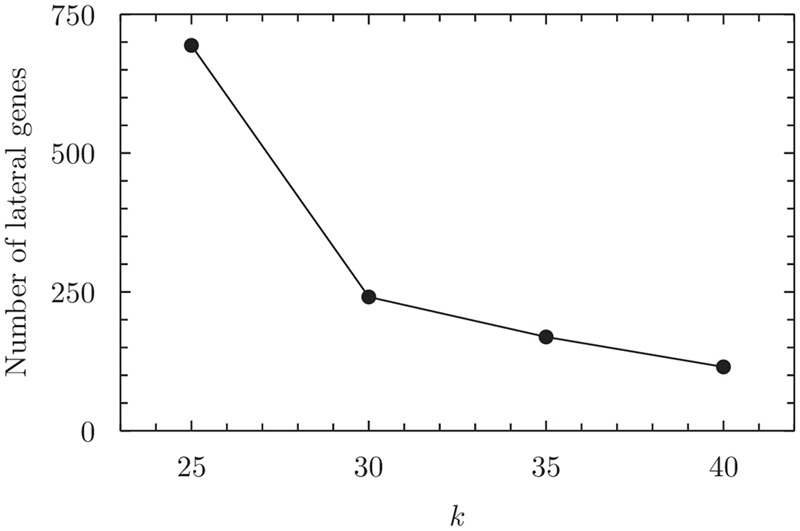
**Number of inferred lateral genes in the BAC dataset at 25 ≤ k ≤ 40.** For numerical values see Supplementary Table 34.

With the BAC dataset we again explore a broad phyletic range (24 orders representing 12 classes across Bacteria); but unlike the situation with BA (above), with BAC we maintain numerical balance (six genomes per order) and a comparable degree of local sequence diversity (each set of six genomes represents a single genus) to the extent possible, given the underlying biology and the availability of high-quality genome sequences. As simulations (Cong et al., 2016a) indicate a high likelihood of false positive detections at *k* = 20, here we vary *k* from 25 to 40 in steps of 5. As above, the total number of genes inferred to be affected by LGT events decreases with increased *k* (**Figure 5**).

At the smallest value of *k* = 25 we infer 81 edges in the LGT network, connecting 17 nodes corresponding to orders of Proteobacteria (12), Low G+C Firmicutes (four) and High G+C Firmicutes (one). The largest clique inferred contains seven orders (Supplementary Figure 16 and Supplementary Table 23). At *k* = 30 only four of these orders remain (Neisseriales, Enterobacteriales, Pasteurellales and Lactobacillales) in the maximum clique, and this clique persists through to *k* = 40 (Supplementary Figures 17–19 and Supplementary Tables 25, 27, and 29). Thus these four orders form the core nodes of the GEC for the BAC dataset. This is in complete agreement with our results from the BA dataset.

At *k* = 25, we also infer Enterobacteriales (represented here by six *E. coli* genomes) to have donated via LGT to 125 genes in other orders and to have accepted LGT from other orders into 111 genes, together 34% of all affected genes across this dataset. These results support the developing themes of LGT being more successful among more-closely related genomes, with enteric bacteria and Firmicutes particularly active.

Other maximal cliques containing more than three orders are also found in the BAC dataset (Supplementary Tables 24, 26, 28, and 30) at different *k*. Some are subsets of the maximum clique, and reflect the fractions of the maximum clique in specific parts of the LGT network, while others are independent of the maximum clique and indicate other regions of dense connection among bacterial orders.

### Enrichment of Biological Processes within Cliques

In addition to clique membership and topology, we are also interested in the biological processes enriched among the genes affected by inferred lateral events, as these may point to physiological, ecological and other processes that help to construct and maintain bacterial communities in nature. Analyses of the LGT networks inferred for the ECS and EB datasets reveal that more than 90% of the genes affected by LGT are represented in the corresponding cliques. In the ECS dataset, all vertices are in the maximum clique. In such cases there is no need to carry out enrichment tests: biological processes contributing to clique formation will be indistinguishable from those of the whole LGT network to which the cliques belong, i.e., the total LGT edge sets (Cong et al., 2016b). For the BAC dataset (Supplementary Table 31), genes annotated for involvement in metabolic processes (e.g., small-molecule and amino acid biosynthesis) are about twice as numerous as those in the next most-numerous category, ribosomal proteins: see the Supplementary Material for Cong et al. (2016b), particularly Section 4.2.

For the BA dataset, however, clique topologies change significantly with *k*. Few LGT events are detected at *k* > 30, particularly when sequences are grouped by phylum (**Figure 4**). For optimal comparison, we carried out enrichment tests on lateral genes of maximum cliques in each network at *k* = 20, 25 or 30, with genomes grouped either by phylum or by class. These tests identify biological processes related to metabolism, transport and regulation as over-represented when sequences are grouped by phylum. The term *translational elongation* (GO:0006414) ranks in first position at *k* = 20, and seventh at *k* = 25, among over-represented terms. The most significantly under-represented biological processes relate to transposition and to RNA modification at *k* = 20, and to RNA processing and biosynthetic processes at *k* = 25. The only term under-represented at *k* = 30 describes the modification of macromolecules.

When the genomes are grouped instead by class, the main categories of GO terms significantly over-represented remain those describing metabolism, transport and regulation. Those most under-represent relate to transposition, RNA metabolisms and regulation at *k* = 20 and 25; at *k* = 30, processes of protein modification are under-represented.

In general, the patterns of over-representation are similar between analyses at phylum and class levels. Interestingly, *translation elongation* is significantly over-represented at phylum level, but much less so at class level. *Transposition* (GO:0032196) is significantly under-represented in most cases.

## Discussion

Here we inferred LGT networks for four datasets of different phyletic breadth, hence evolutionary depth. For the ECS dataset, the entire LGT network is captured within a single clique encompassing all nodes, consistent with previous research (Skippington and Ragan, 2012). Interplay with the IDF threshold is seen clearly with the EB dataset and its variants. For the full EB dataset (EB-1), the LGT signal between *Escherichia* and *Shigella* is much stronger than that of any other pairwise comparison and dominates the lateral signal, with the result that the only community that can be found is *Escherichia* and *Shigella*. If we remove *Escherichia* or (alternatively) *Shigella*, or combine them into a single group, we detect LGT events from (and/or to) *Klebsiella* and *Salmonella*. This reveals a larger clique containing either *Escherichia* or *Shigella*, plus *Klebsiella* and *Salmonella* (Supplementary Figures 2–5). By contrast, *Yersinia* is relatively silent to LGT, and contributes little to the community.

Particularly in the BA dataset, we see that different parts of the LGT network are differentially sensitive to change of *k*. When *k* is small (here *k* = 20), many *k*-mers are shared by chance, resulting in many false positive inferences (Cong et al., 2016a,b). Edges supported by large numbers of lateral events (e.g., those with high weights) tend to persist, whereas those representing smaller numbers of events may disappear as *k* is incremented. Even so, when the sequences are grouped by phylum, the High-G+C Firmicutes, Low-G+C Firmicutes and Proteobacteria are found in all cliques inferred across the investigated range of parameter values (Supplementary Figures 6–10, Supplementary Tables 2–11). For this reason we identify them as core nodes of the GEC for the BA phyla. Although it does not contribute many LGT events, *Thermus*/*Deinococcus* is also a member of most communities.

When the BA dataset is grouped into 31 classes, many more clique structures are found. The α-, β- and γ-Proteobacteria, Actinomycetales and *Bacillus*/*Clostridium* are always present in at least one clique (Supplementary Figures 11–15, Supplementary Tables 12–21), i.e., are core nodes. This agrees with an earlier conclusion, based on classical alignment-based phylogenomic methods, that these groups are connected by major highways of LGT (Beiko et al., 2005). By contrast, the 𝜀-Proteobacteria appear relatively silent to LGT, with fewer inferred events per genome (Supplementary Table 22). In the class-level LGT network, the sole *Deinococcus* genome is also involved in many (maximum and maximal) cliques, linked through a lateral edge with subdivisions from Proteobacteria. Stronger connectivity might be expected if more sequences from Deinococci and its immediate relatives were represented in this dataset.

Although many fewer instances of LGT are inferred involving archaea, we nonetheless recognize one clique among them. The low frequency of inferred LGT events may arise because these genomes are relatively diverse in gene content and phylogenetically distant from each other, and/or because in reality these genomes have exchanged little genetic material, for example because they live in specialized environments (Beiko et al., 2005; Popa et al., 2011). In the former case TF-IDF should find instances of LGT but the pairwise values may not pass the IDF threshold, whereas in the latter case there would be little true-positive LGT to be found and lowering the IDF threshold would lead only to false-positive inferences. Comparing the results of TF-IDF with those of classical alignment-based methods may help distinguish between these alternative explanations.

Enrichment tests on the BA data reveal that a wide range of biological processes are over-represented in the LGT events that underpin the cliques identified. As expected (Jain et al., 1999, 2003), metabolic processes, gene regulation, and trans-membrane and intracellular transport are broadly represented. For example, at *k* = 25 with genomes grouped by class, 39 of the 50 most over-represented processes describe metabolism. Terms associated with transposition or antibiotic resistance are not seen: these genes are usually transferred within-phylum or within-class (or indeed more narrowly) and often occur on plasmids, which are not represented in the genome data files we used. As expected, few terms describing processes of transcription, translation or DNA replication (Jain et al., 1999, 2003) are overrepresented.

Fewer biological process terms are under-represented among the LGT events that underpin the BA cliques, although *transposition* (GO:0032196) is very significantly under-represented. A similar result was also found for the ECS dataset (Supplementary Table 4). From previous research (Cong et al., 2016b) we know that genes annotated with this term are widespread in the ECS genomes, making it difficult for genes annotated with this term to pass the TF threshold for detection. In the BA dataset, genomes of *E. coli* and *Shigella* are a major source of genes associated with transposition; as these are members of the same group (γ-Proteobacteria), they are not detected by TF-IDF. In the EB dataset, when *Escherichia* and *Shigella* are not treated as separate groups, *transposition* is not significantly under-represented (Supplementary Table 5). Thus TF-IDF is not blind to such mobile biological processes, but the way groups are delimited can limit their discovery.

This work represents the first systematic exploration of the sensitivity of densely connected structures (maximum and maximal cliques) in LGT graphs to choice of parameter values in an alignment-free framework. Our workflow is the first to implement alignment-free and other highly scalable methods end-to-end, from whole genome sequences to delineation GECs and functional analysis of the genes affected by LGT. Our results confirm the promise of this approach, notably the robustness of clique structure and membership at sufficiently large *k*, here *k* ≥ 25. Nonetheless, important challenges remain.

Computational simulations and empirical studies demonstrate that approaches based on *k*-mer count can support the scalable inference of phylogenies (Chan et al., 2014; Bernard et al., 2016a,b) and identify regions of lateral transfer within a dataset (Cong et al., 2016a,b). Parameters including *k* can be adjusted to minimize the effects of sequence divergence and genome rearrangement. However, word-count methods are less robust to sequence loss or truncation (Chan et al., 2014). As is the case with classical phylogenetics, other scenarios likely to erode the performance of word-count methods include compositional bias and/or rate variation within genomes or across lineages, including convergent processes in distantly related sequences. Methods will need to be developed such that alignment-free approaches, including TF-IDF, can mitigate or avoid these situations.

Graph-theoretical research has primarily concentrated on difficult combinatorial problems posed on finite, simple graphs. Graph analytical software packages such as GrAPPA (Langston Lab, the University of Tennessee^2^), therefore, are designed mainly for undirected, unweighted graphs. This has required us to ignore both directionality (by merger of incoming and outgoing edges) and weights. Such simplifications represent a classic pre-processing step for a directed network (Seidman and Foster, 1978). While other strategies have been introduced to find cliques in directed networks, all involve weakening the edges, and none can guarantee a better interpretation of properties of the original directed network (Seidman, 1980; Palla et al., 2007). Comparing these approaches across various application domains remains an open problem. Despite this limitation, some features of the role played by LGT in the evolution of microbes are still accessible. A good example is the frequent exchange inferred among *Escherichia* and *Shigella* contrasted with the relative isolation of *Yersinia*.

It is noteworthy that we have defined GECs as cliques, because the clique is a rigorous graph-theoretical structure that maps particularly well onto numerous biological concepts, in the present case the sharing of genetic information via LGT. While this makes sense in the quest for biological fidelity, in mapping GECs onto LGT graphs Skippington and Ragan (2011) expressed concern that missing data might make clique too rigorous a definition. We share this reservation, and observe that noise-resilient options such as paraclique may fare better. We need only a criterion, e.g., paraclique’s *glom* term (Hagan et al., 2016), by which to estimate the number or proportion of “missing” edges. An exploration of such criteria may be the subject of future work.

## Author Contributions

All authors designed the experiments. YC implemented and carried out the computational analyses. CP and ML provided software. All authors analyzed the data, and wrote and edited the paper.

## Conflict of Interest Statement

The authors declare that the research was conducted in the absence of any commercial or financial relationships that could be construed as a potential conflict of interest.

## Footnotes

1 https://grappa.eecs.utk.edu/

2 https://grappa.eecs.utk.edu/
