# Supplementary material for: Robust Inference of Genetic Exchange Communities from Microbial Genomes Using TF-IDF
Source: Front Microbiol. 2017 Jan 19;8:21. doi: 10.3389/fmicb.2017.00021 (PMC5243798; doi:10.3389/fmicb.2017.00021)
Supplement: Supplementary file 1 [file Data_Sheet_1.pdf]

Supplementary Material

Robust inference of genetic exchange communities from microbial genomes using TF-IDF

Yingnan Cong, Yao-ban Chan, Charles A. Phillips, Michael A. Langston, Mark A. Ragan*

*** Correspondence:** Mark Ragan: m.ragan@uq.edu.au

Overview of Supplementary Material:

Section 1. Supplementary material for EB and BA datasets. LGT networks inferred for different datasets at different values of *k*.

Supplementary Figures 1-15. *Note:* High-resolution versions of Figures S6 - S15 are available for download at http://bioinformatics.org.au/tools-data/

Section 2. Distribution of lengths of lateral segments inferred for the ECS dataset, at different values of *k*.

Supplementary Table 1

Section 3. Cliques in the BA dataset and its variants, with different values of *k* and grouping.

Supplementary Tables 2-21

Section 4. Connectivity of classes within Proteobacteria.

Supplementary Table 22

Section 5. LGT networks inferred for the BAC dataset at different values of *k*.

Supplementary Figures 16-19. *Note:* High-resolution versions of Supplementary Figures 16 - 19 are available for download at http://bioinformatics.org.au/tools-data/

Section 6. Cliques in the BAC dataset at different values of *k*.

Supplementary Tables 23-30

Section 7. Lateral genes inferred in BAC dataset, *k* = 25.

Supplementary Table 31

Section 8. Numerical data supporting main text Figures 4 and 5.

Supplementary Tables 32-34

Section 9. Genomes, NCBI accession numbers and group information for the ECS, EB, BA and BAC datasets.

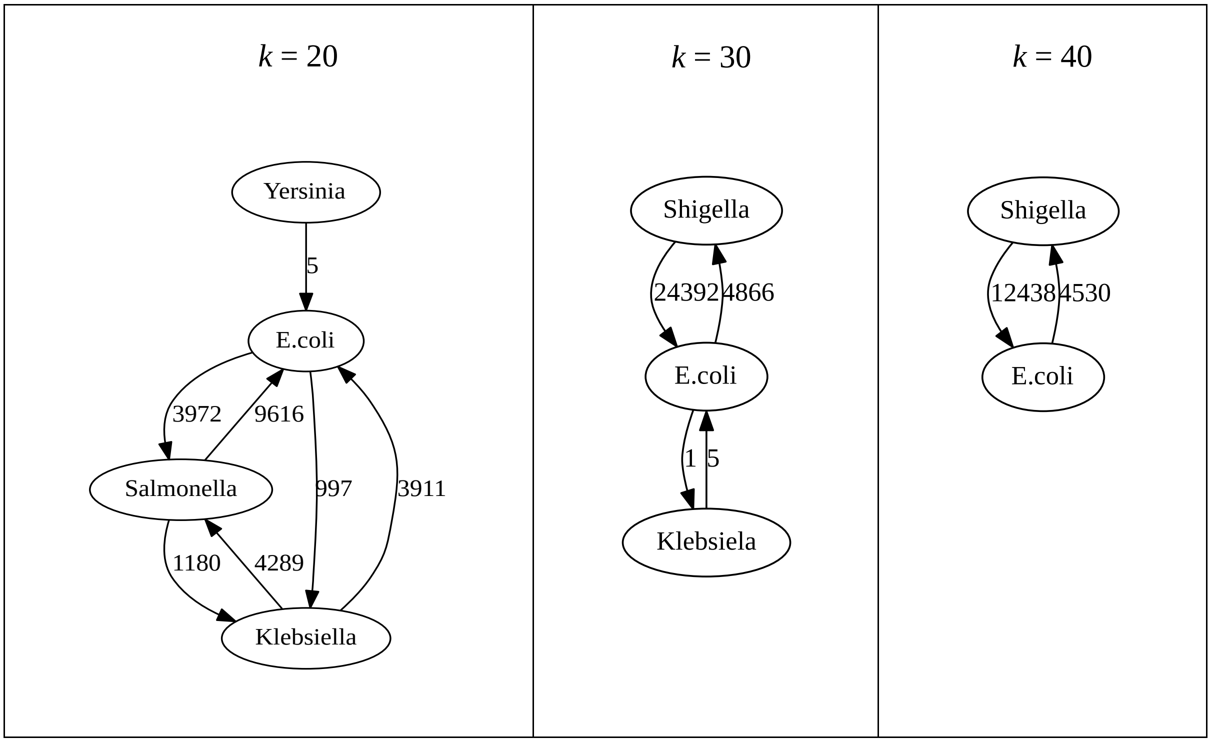

Supplementary Figure 1. LGT networks for EB-1 dataset at different values of *k*.

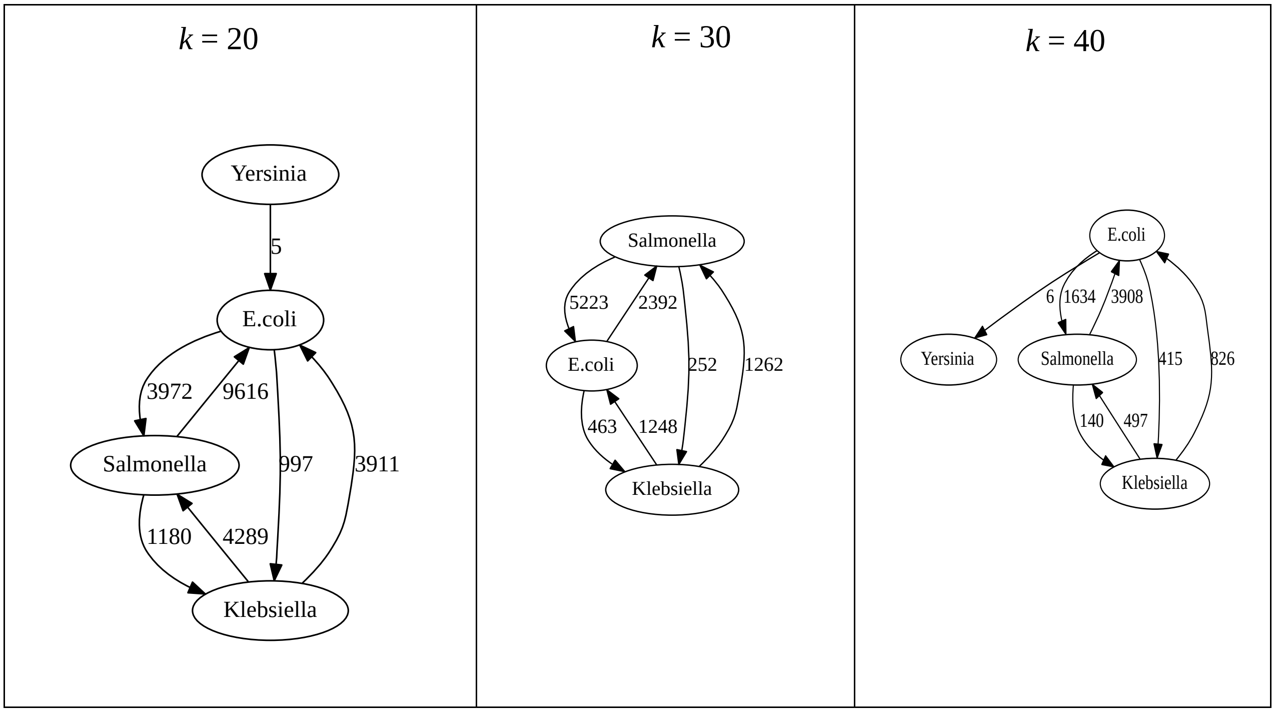

Supplementary Figure 2. LGT networks for the EB-2 dataset at different values of *k*.

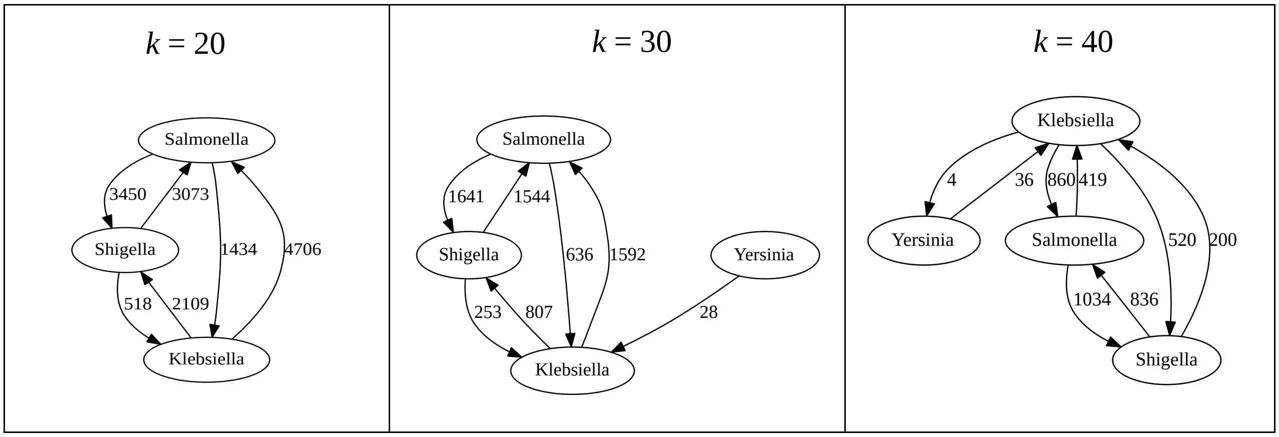

Supplementary Figure 3. LGT networks for the EB-3 dataset at different values of *k*.

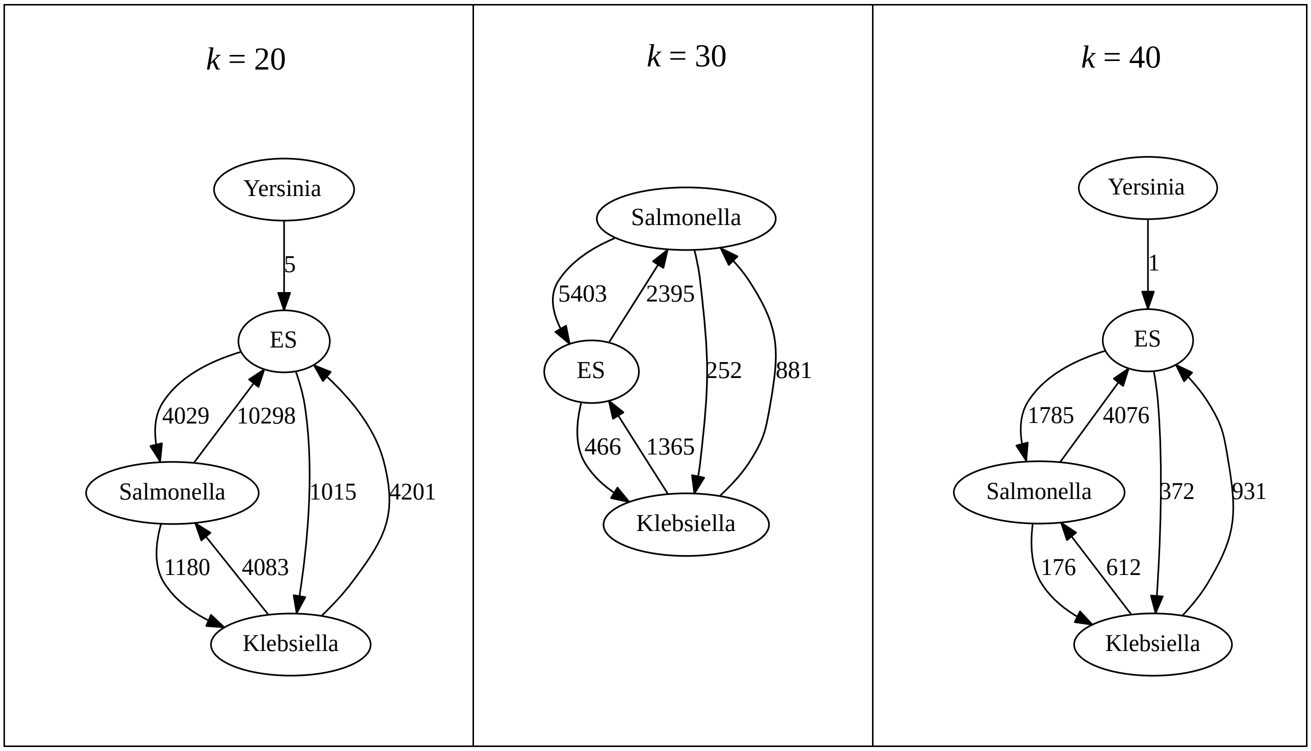

Supplementary Figure 4. LGT networks for the EB-4 dataset at different values of *k*.

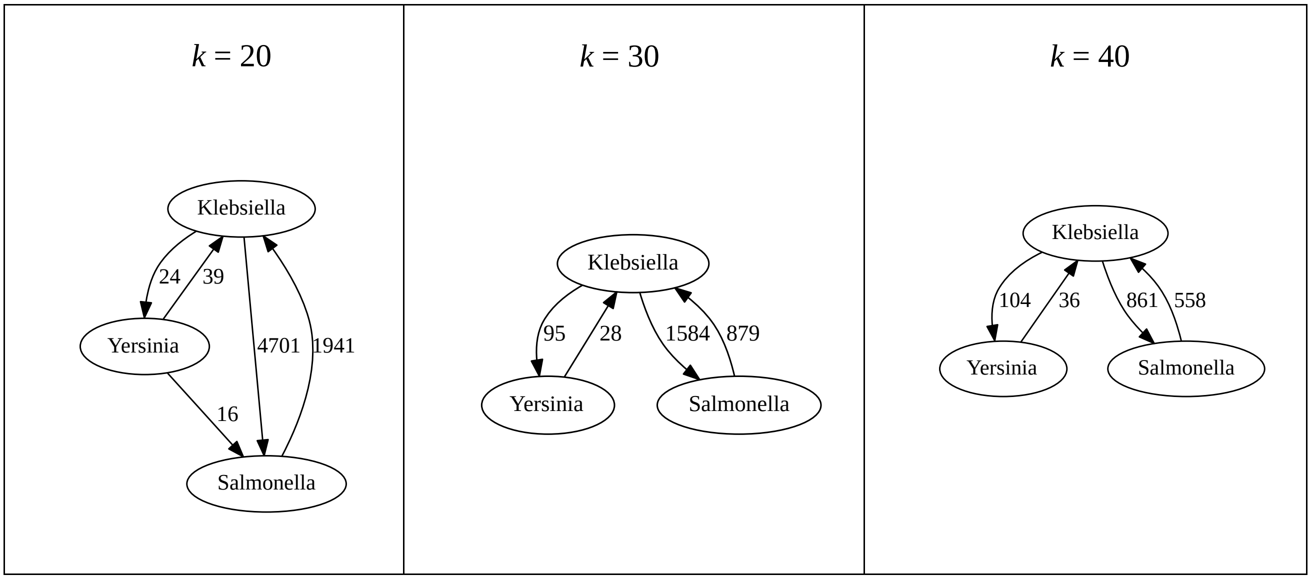

Supplementary Figure 5. LGT networks for the EB-5 dataset at different values of *k*.

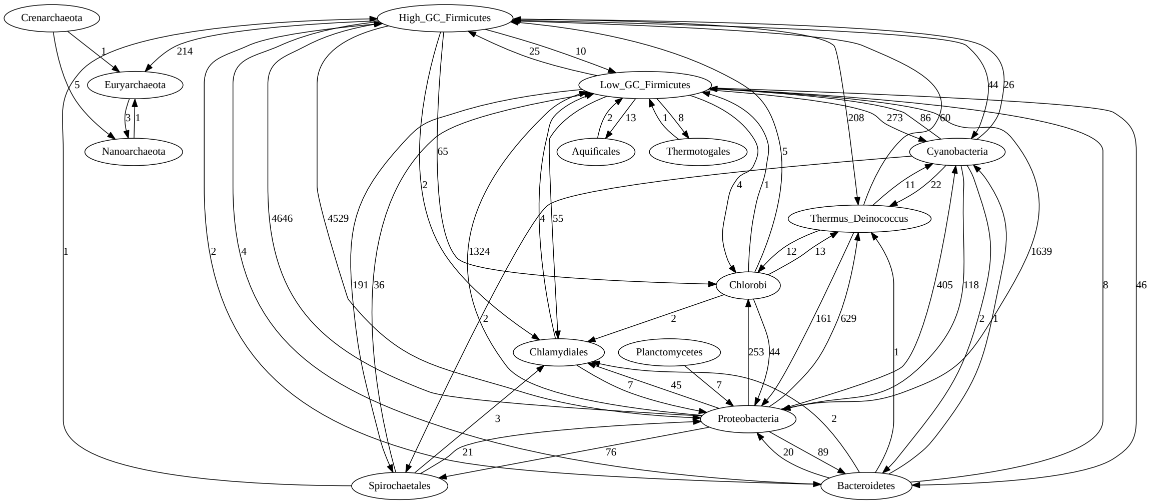

Supplementary Figure 6. LGT network for the BA dataset: genomes grouped by phylum, *k* = 20.

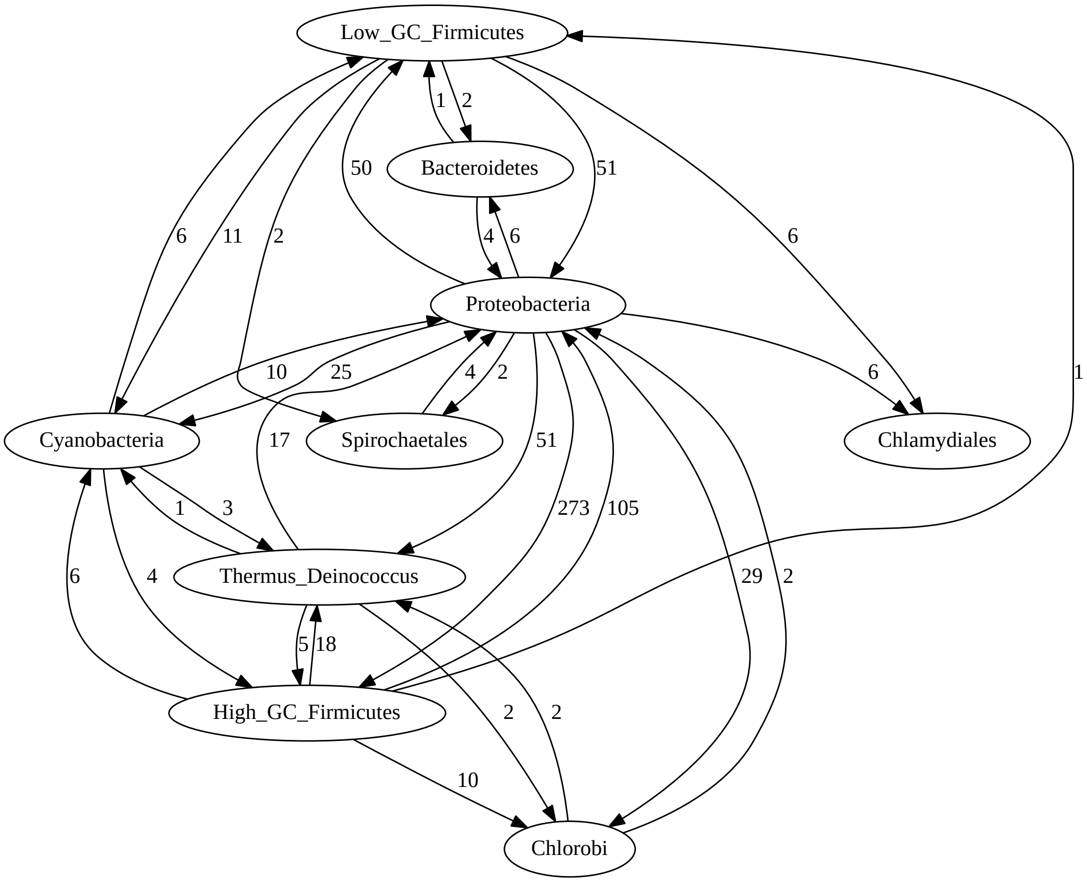

Supplementary Figure 7. LGT network for the BA dataset: genomes grouped by phylum, *k* = 25.

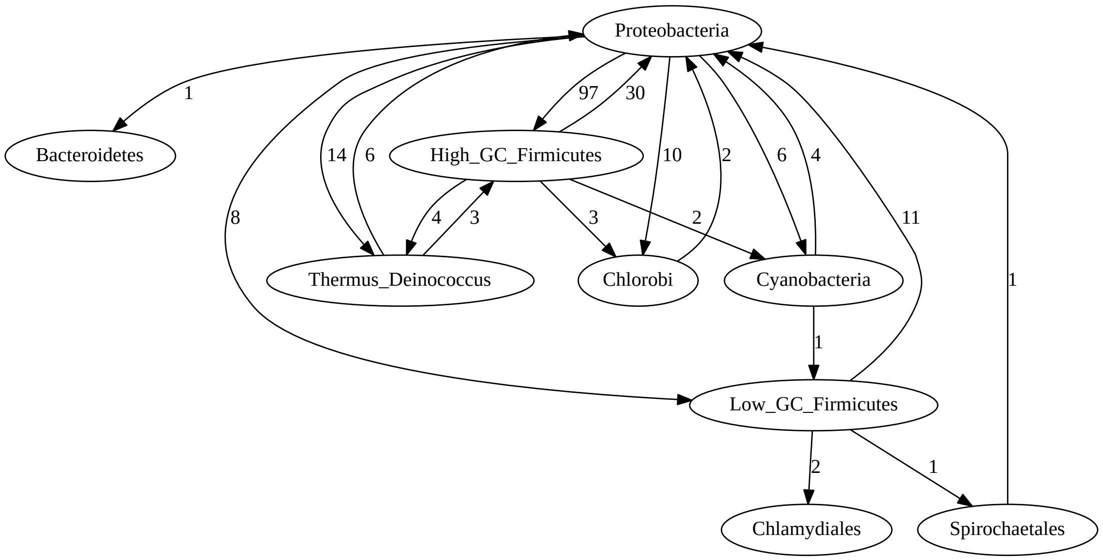

Supplementary Figure 8. LGT network for the BA dataset: genomes grouped by phylum, *k* = 30.

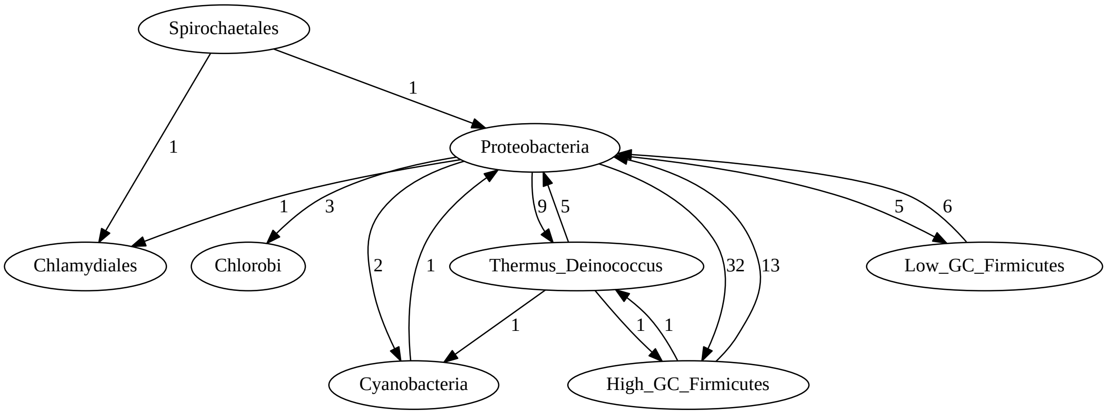

Supplementary Figure 9. LGT network for the BA dataset: genomes grouped by phylum, *k* = 35.

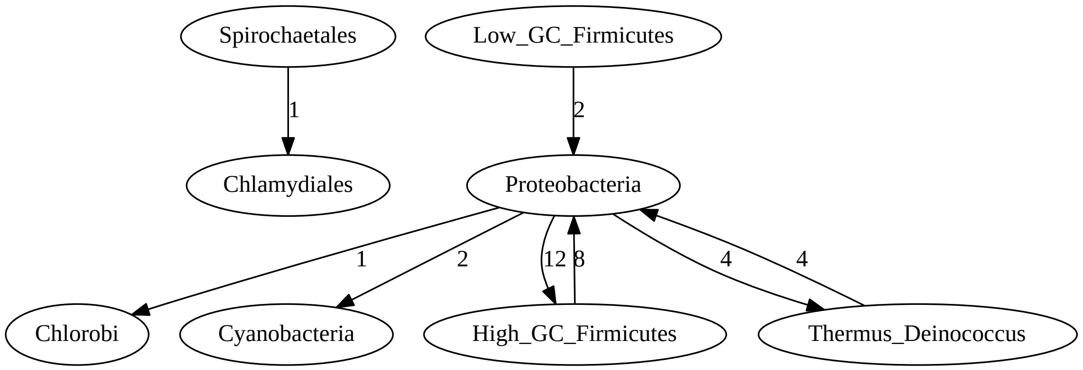

Supplementary Figure 10. LGT network for the BA dataset: genomes grouped by phylum, *k* = 40.

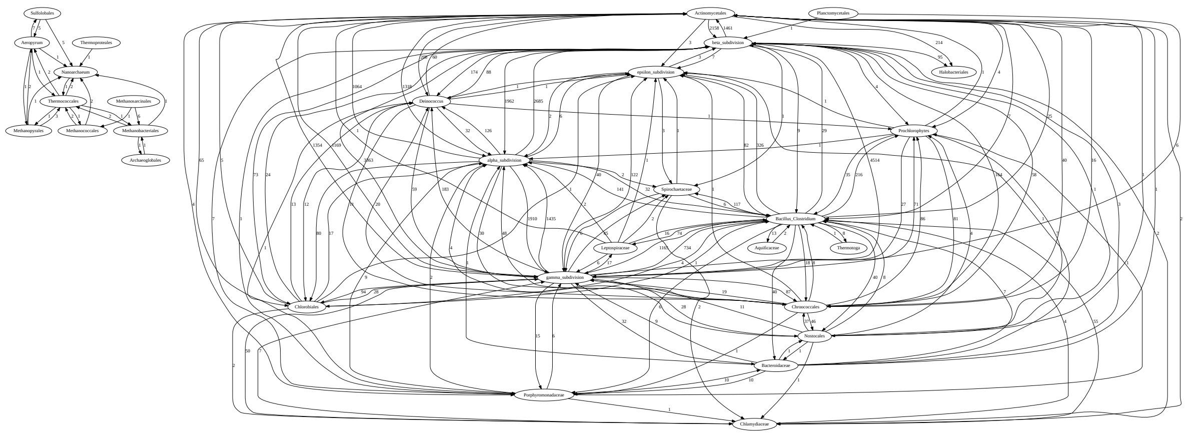

Supplementary Figure 11. LGT network for the BA dataset: genomes grouped by class, *k* = 20.

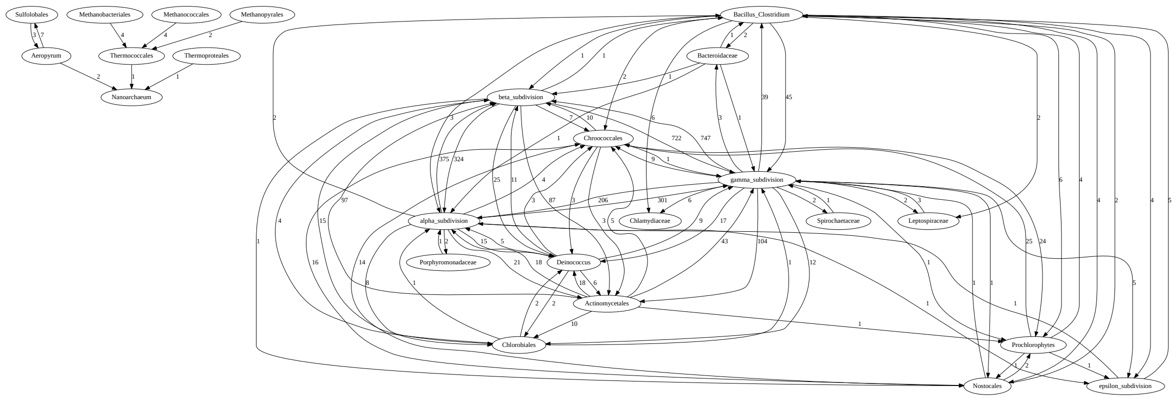

Supplementary Figure 12. LGT network for the BA dataset: genomes grouped by class, *k* = 25.

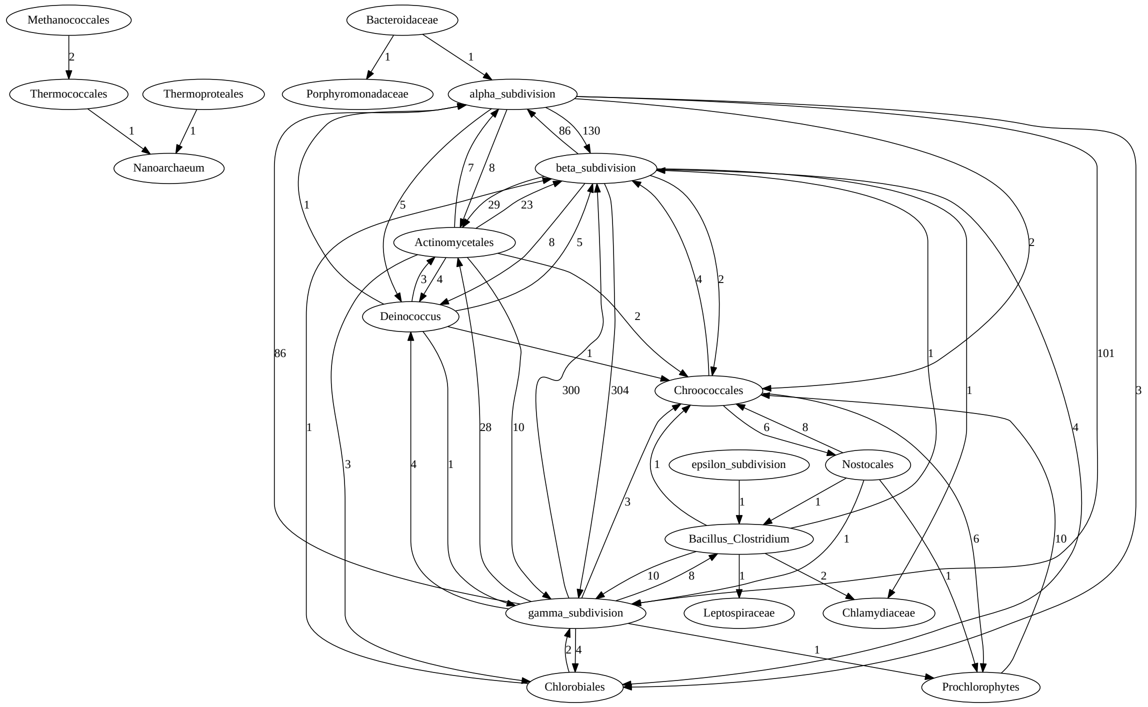

Supplementary Figure 13. LGT network for the BA dataset: genomes grouped by class, *k* = 30.

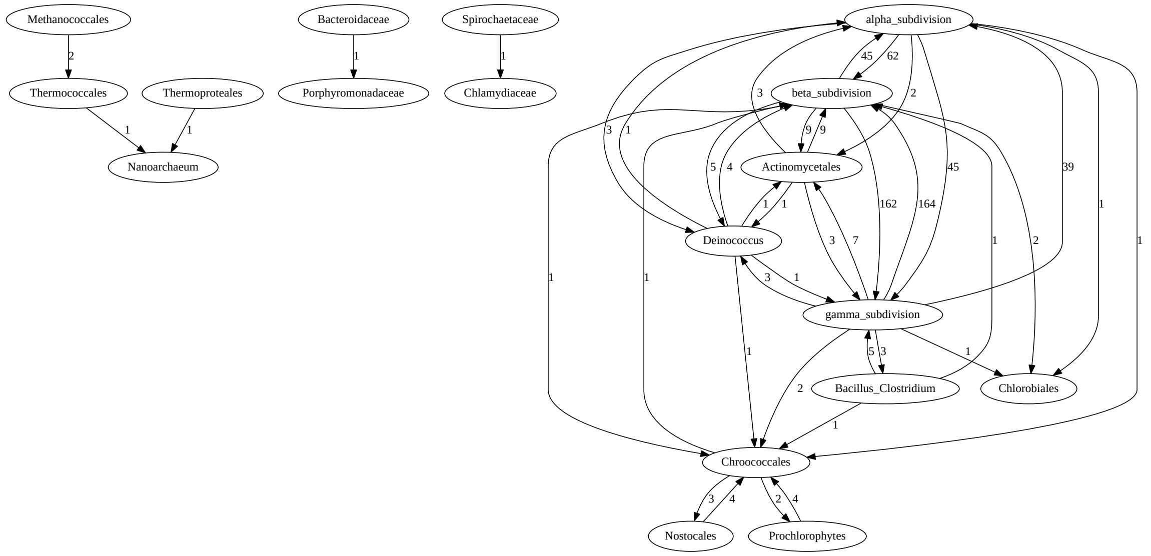

Supplementary Figure 14. LGT network for the BA dataset: genomes grouped by class, *k* = 35.

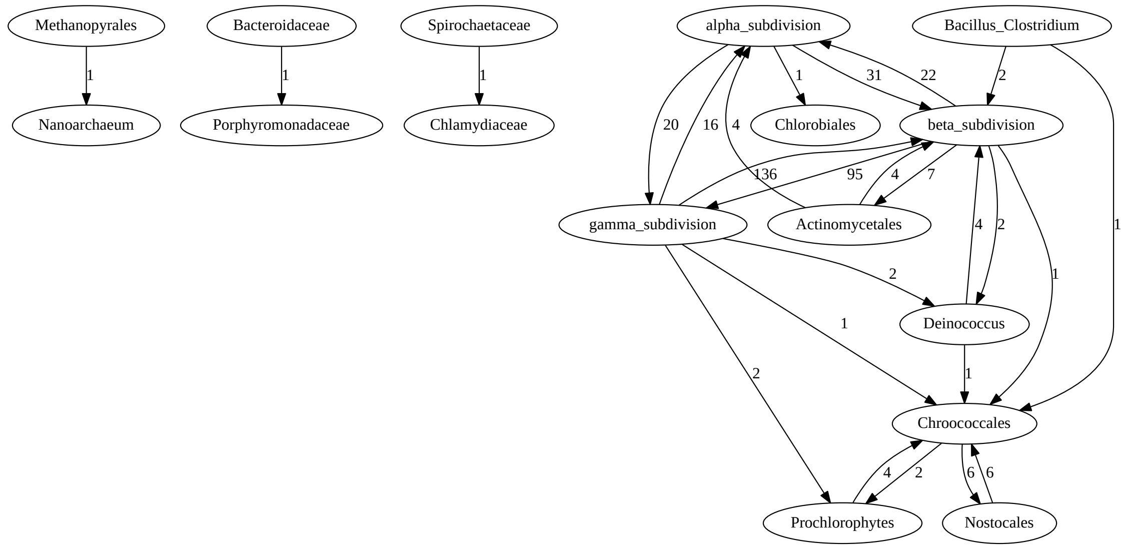

Supplementary Figure 15. LGT network for the BA dataset: genomes grouped by class, *k* = 40.

Supplementary Table 1. Distributions of lengths of inferred lateral segments in the ECS dataset at different *k*.

**Length of lateral segment (nt)**	***k* = 20**	***k* = 30**	***k* = 40**
0-100	41587	37297	27972
100-200	21620	26318	23783
200-300	13343	17161	16043
300-400	8890	11240	11333
400-500	6189	8394	8510
500-600	4656	6261	6235
600-700	3674	4814	5148
700-800	2971	3824	3926
800-900	2281	2894	3286
900-1000	1929	2449	2614
1000-1100	1634	2037	2187
1100-1200	1417	1644	1845
1200-1300	1264	1215	1539
1300-1400	970	1074	1354
1400-1500	917	1018	1106
1500-1600	736	824	991
1600-1700	659	701	844
1700-1800	625	789	722
1800-1900	581	488	563
1900-2000	452	449	598
2000-2100	399	432	504
2100-2200	413	414	460
2200-2300	359	335	424
2300-2400	320	358	379
2400-2500	314	283	342
2500-2600	255	272	267
2600-2700	260	216	275
2700-2800	218	159	236
2800-2900	224	225	214
2900-3000	190	171	213
3000-3100	210	166	182
3100-3200	182	143	153
3200-3300	157	142	157
3300-3400	153	121	138
3400-3500	179	136	165
3500-3600	123	126	119
3600-3700	125	85	104
3700-3800	121	87	98
3800-3900	91	87	109
3900-4000	86	82	81

4000-4100	68	73	78
4100-4200	80	44	66
4200-4300	52	44	82
4300-4400	77	67	72
4400-4500	72	65	45
4500-4600	63	65	56
4600-4700	71	40	51
4700-4800	65	50	51
4800-4900	50	58	51
4900-5000	44	34	31
5000+	1164	858	868

Supplementary Table 2. BA dataset grouped by phylum, *k* = 20; maximum clique as the GEC.

1	2	3	4	5
Spirochaetales	Chlamydiales	High_GC_Firmicutes	Low_GC_Firmicutes	Proteobacteria

Supplementary Table 3. BA dataset grouped by phylum, *k* = 20; maximal cliques as the GECs.

1	2	3	4	5
High_GC_Firmicutes	Proteobacteria	Low_GC_Firmicutes	Chlamydiales	Bacteroidetes
High_GC_Firmicutes	Proteobacteria	Low_GC_Firmicutes	Chlamydiales	Chlorobi
High_GC_Firmicutes	Proteobacteria	Low_GC_Firmicutes	Chlamydiales	Spirochaetales
High_GC_Firmicutes	Proteobacteria	Low_GC_Firmicutes	Cyanobacteria	Bacteroidetes
High_GC_Firmicutes	Proteobacteria	Low_GC_Firmicutes	Cyanobacteria	Spirochaetales
High_GC_Firmicutes	Proteobacteria	Thermus_Deinococcus	Bacteroidetes	Cyanobacteria
High_GC_Firmicutes	Proteobacteria	Thermus_Deinococcus	Chlorobi	
Nanoarchaeota	Euryarchaeota	Crenarchaeota		

Supplementary Table 4. BA dataset grouped by phylum, *k* = 25; maximum clique as the GEC.

1	2	3	4
Thermus_Deinococcus	Cyanobacteria	High_GC_Firmicutes	Proteobacteria

Supplementary Table 5. BA dataset grouped by phylum, *k* = 25; maximal cliques as the GECs.

1	2	3	4
Proteobacteria	Low_GC_Firmicutes	High_GC_Firmicutes	Cyanobacteria
Proteobacteria	Low_GC_Firmicutes	Bacteroidetes	
Proteobacteria	Low_GC_Firmicutes	Chlamydiales	
Proteobacteria	Low_GC_Firmicutes	Spirochaetales	
Proteobacteria	Chlorobi	Thermus_Deinococcus	High_GC_Firmicutes
Proteobacteria	Thermus_Deinococcus	Cyanobacteria	High_GC_Firmicutes

Supplementary Table 6. BA dataset grouped by phylum, *k* = 30; maximum clique as the GEC.

1	2	3
Thermus_Deinococcus	High_GC_Firmicutes	Proteobacteria

Supplementary Table 7. BA dataset grouped by phylum, *k* = 30; maximal cliques as the GECs.

1	2	3
Proteobacteria	High_GC_Firmicutes	Chlorobi
Proteobacteria	High_GC_Firmicutes	Cyanobacteria
Proteobacteria	High_GC_Firmicutes	Thermus_Deinococcus
Proteobacteria	Low_GC_Firmicutes	Cyanobacteria
Proteobacteria	Low_GC_Firmicutes	Spirochaetales

Supplementary Table 8. BA dataset grouped by phylum, *k* = 35; maximum clique as the GEC.

1	2	3
High_GC_Firmicutes	Thermus_Deinococcus	Proteobacteria

Supplementary Table 9. BA dataset grouped by phylum, *k* = 35; maximal cliques as the GECs.

1	2	3
Proteobacteria	Thermus_Deinococcus	Cyanobacteria
Proteobacteria	Thermus_Deinococcus	High_GC_Firmicutes
Proteobacteria	Spirochaetales	Chlamydiales

Supplementary Table 10. BA dataset grouped by phylum, *k* = 40; maximum clique as the GEC.

1	2
Thermus_Deinococcus	Proteobacteria

Supplementary Table 11. BA dataset grouped by phylum, *k* = 40; maximal cliques as the GECs.

1	2
Proteobacteria	Chlorobi
Proteobacteria	Cyanobacteria
Proteobacteria	High_GC_Firmicutes
Proteobacteria	Low_GC_Firmicutes
Proteobacteria	Thermus_Deinococcus
Chlamydiales	Spirochaetales

Supplementary Table 12. BA dataset grouped by class, *k* = 20; maximum clique as the GEC.

1	2	3	4	5	6	7	8
Chroococcales	Prochlorophytes	epsilon_subdivision	alpha_subdivision	Actinomycetales	gamma_subdivision	Bacillus_Clostridium	beta_subdivision

Supplementary Table 13. BA dataset grouped by class, *k* = 20; maximal cliques as the GECs.

1	2	3	4	5	6	7	8
beta_subdivision	gamma_subdivision	Bacillus_Clostridium	Actinomycetales	alpha_subdivision	Chroococcales	Prochlorophytes	Nostocales
beta_subdivision	gamma_subdivision	Bacillus_Clostridium	Actinomycetales	alpha_subdivision	Chroococcales	Prochlorophytes	Porphyromonadaceae
beta_subdivision	gamma_subdivision	Bacillus_Clostridium	Actinomycetales	alpha_subdivision	Chroococcales	Prochlorophytes	epsilon_subdivision
beta_subdivision	gamma_subdivision	Bacillus_Clostridium	Actinomycetales	alpha_subdivision	Bacteroidaceae	Nostocales	
beta_subdivision	gamma_subdivision	Bacillus_Clostridium	Actinomycetales	alpha_subdivision	Bacteroidaceae	Porphyromonadaceae	
beta_subdivision	gamma_subdivision	Bacillus_Clostridium	Actinomycetales	alpha_subdivision	Bacteroidaceae	epsilon_subdivision	
beta_subdivision	gamma_subdivision	Bacillus_Clostridium	Actinomycetales	alpha_subdivision	Chlorobiales	epsilon_subdivision	
beta_subdivision	gamma_subdivision	Bacillus_Clostridium	Actinomycetales	Chlamydiaceae	Porphyromonadaceae		
beta_subdivision	gamma_subdivision	Bacillus_Clostridium	Actinomycetales	Chlamydiaceae	Nostocales		
beta_subdivision	gamma_subdivision	Bacillus_Clostridium	Actinomycetales	Chlamydiaceae	epsilon_subdivision		
beta_subdivision	gamma_subdivision	Bacillus_Clostridium	Actinomycetales	Chlamydiaceae			
beta_subdivision	gamma_subdivision	Bacillus_Clostridium	Spirochaetaceae	Chlamydiaceae			
beta_subdivision	gamma_subdivision	Deinococcus	Actinomycetales	alpha_subdivision	Chroococcales	Prochlorophytes	Porphyromonadaceae
beta_subdivision	gamma_subdivision	Deinococcus	Actinomycetales	alpha_subdivision	Chroococcales	Prochlorophytes	epsilon_subdivision
beta_subdivision	gamma_subdivision	Deinococcus	Actinomycetales	alpha_subdivision	Chlorobiales	epsilon_subdivision	
beta_subdivision	gamma_subdivision	Planctomycetales					
beta_subdivision	Halobacteriales	Actinomycetales					
Aeropyrum	Nanoarchaeum	Thermococcales	Methanopyrales				
Aeropyrum	Nanoarchaeum	Sulfolobales					
Thermococcales	Nanoarchaeum	Methanobacteriales					
Thermococcales	Nanoarchaeum	Methanococcales					
Leptospiraceae	Bacillus_Clostridium	gamma_subdivision	alpha_subdivision	epsilon_subdivision	Actinomycetales		
Leptospiraceae	Bacillus_Clostridium	gamma_subdivision	alpha_subdivision	epsilon_subdivision	Spirochaetaceae		

Supplementary Table 14. BA dataset grouped by class, *k* = 25; maximum clique as the GEC.

1	2	3	4	5	6
Deinococcus	Actinomycetales	Chroococcales	beta_subdivision	alpha_subdivision	gamma_subdivision

Supplementary Table 15. BA dataset grouped by class, *k* = 25; maximal cliques as the GECs.

1	2	3	4	5	6
gamma_subdivision	Bacillus_Clostridium	alpha_subdivision	beta_subdivision	Bacteroidaceae	
gamma_subdivision	Bacillus_Clostridium	alpha_subdivision	beta_subdivision	Chroococcales	
gamma_subdivision	Bacillus_Clostridium	alpha_subdivision	epsilon_subdivision		
gamma_subdivision	Bacillus_Clostridium	Chlamydiaceae			
gamma_subdivision	Bacillus_Clostridium	Nostocales	Chroococcales	beta_subdivision	
gamma_subdivision	Bacillus_Clostridium	Nostocales	Chroococcales	Prochlorophytes	
gamma_subdivision	Bacillus_Clostridium	Prochlorophytes	epsilon_subdivision		
gamma_subdivision	Bacillus_Clostridium	Leptospiraceae			
gamma_subdivision	Chlorobiales	alpha_subdivision	Actinomycetales	beta_subdivision	Deinococcus
gamma_subdivision	Actinomycetales	Chroococcales	beta_subdivision	Deinococcus	alpha_subdivision
gamma_subdivision	Actinomycetales	Chroococcales	Prochlorophytes		

Supplementary Table 16. BA dataset grouped by class, *k* = 30; maximum clique as the GEC.

1	2	3	4	5	6
Deinococcus	Actinomycetales	alpha_subdivision	beta_subdivision	Chroococcales	gamma_subdivision

Supplementary Table 17. BA dataset grouped by class, *k* = 30; maximal cliques as the GECs.

1	2	3	4	5	6
gamma_subdivision	Chroococcales	beta_subdivision	Actinomycetales	alpha_subdivision	Deinococcus
gamma_subdivision	Chroococcales	beta_subdivision	Bacillus_Clostridium		
gamma_subdivision	Chroococcales	Nostocales	Bacillus_Clostridium		
gamma_subdivision	Chroococcales	Nostocales	Prochlorophytes		
gamma_subdivision	Chlorobiales	Actinomycetales	beta_subdivision	alpha_subdivision	
Chlamydiaceae	Bacillus_Clostridium	beta_subdivision			

Supplementary Table 18. BA dataset grouped by class, *k* = 35; maximum clique as the GEC.

1	2	3	4	5
Actinomycetales	Deinococcus	alpha_subdivision	gamma_subdivision	beta_subdivision

Supplementary Table 19. BA dataset grouped by class, *k* = 35; maximal cliques as the GECs.

1	2	3	4	5

beta_subdivision	gamma_subdivision	alpha_subdivision	Deinococcus	Chroococcales
beta_subdivision	gamma_subdivision	alpha_subdivision	Deinococcus	Actinomycetales
beta_subdivision	gamma_subdivision	alpha_subdivision	Chlorobiales	
beta_subdivision	gamma_subdivision	Bacillus_Clostridium	Chroococcales	

Supplementary Table 20. BA dataset grouped by class, *k* = 40; maximum clique as the GEC.

1	2	3	4
Deinococcus	gamma_subdivision	Chroococcales	beta_subdivision

Supplementary Table 21. BA dataset grouped by class, *k* = 40; maximal cliques as the GECs.

1	2	3	4
Chroococcales	beta_subdivision	gamma_subdivision	Deinococcus
Chroococcales	beta_subdivision	Bacillus_Clostridium	
Chroococcales	Prochlorophytes	gamma_subdivision	
alpha_subdivision	beta_subdivision	gamma_subdivision	
alpha_subdivision	beta_subdivision	Actinomycetales	

Supplementary Table 22. Lateral events inferred involving Proteobacteria. In-degree, number of genes affected by lateral events; out-degree, number of LGT events originating from this node. Nodes in the network represent classes; *k* = 25, *G* = 2*k*.

Class name	In-degree	Out-degree	Total degree	Number of sequences	Number of LGT events per genome
Alpha-	560	729	1289	9	143
Beta-	1246	1182	2428	8	303
Gamma-	1128	1154	2282	33	69
Epsilon-	11	6	17	5	3

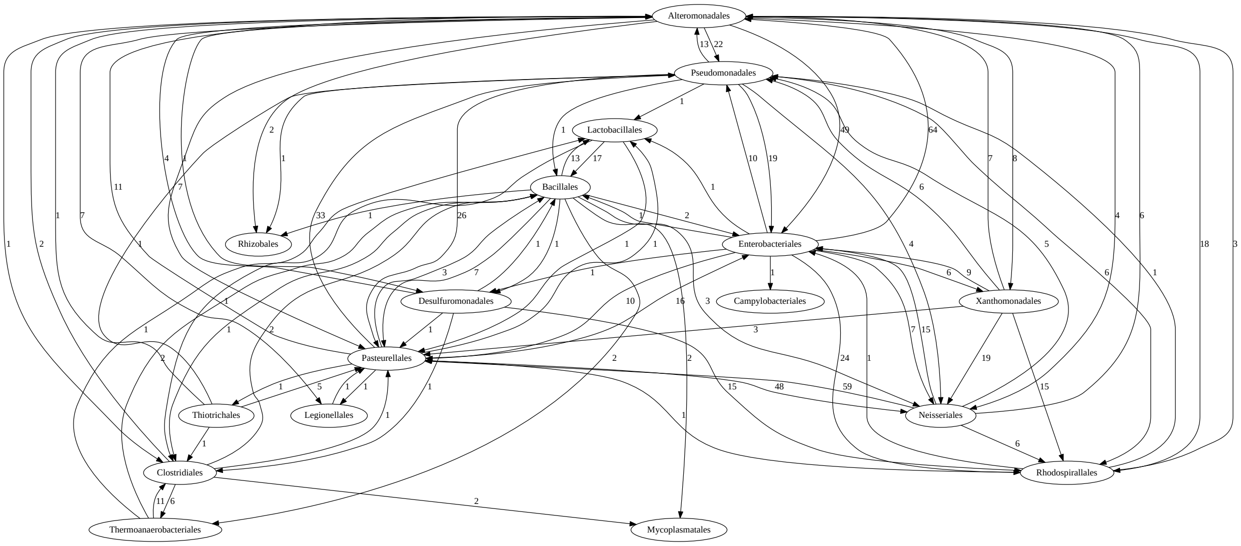

Supplementary Figure 16. LGT network for the BAC dataset, *k* = 25.

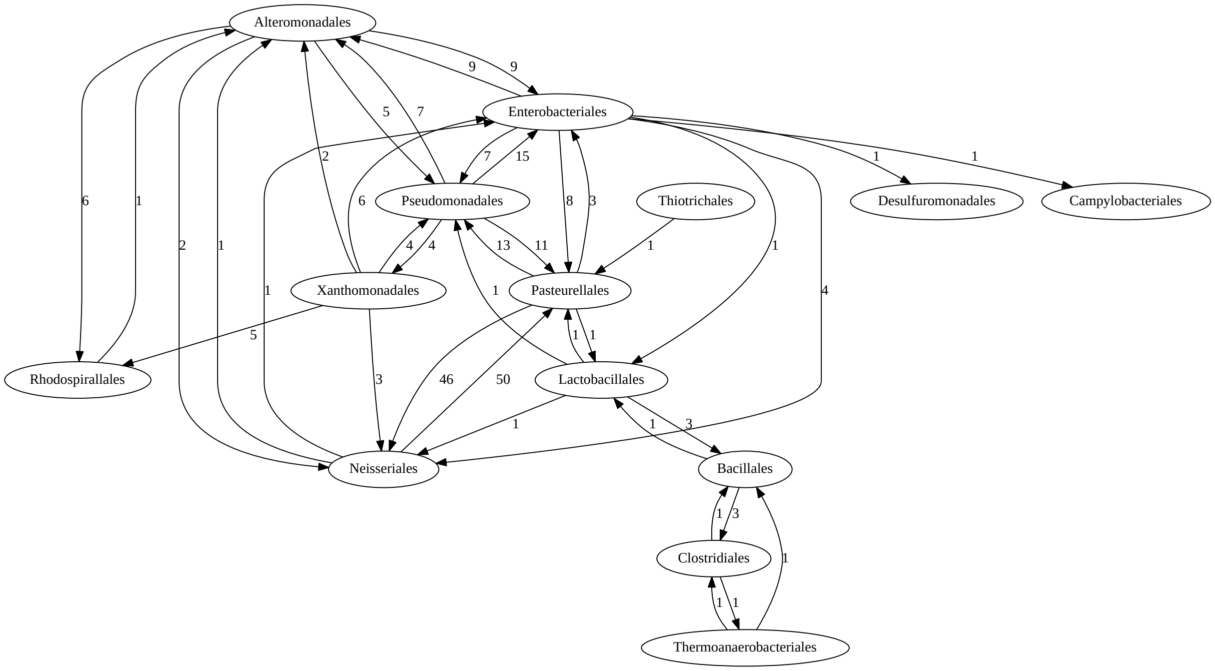

Supplementary Figure 17. LGT network for the BAC dataset, *k* = 30.

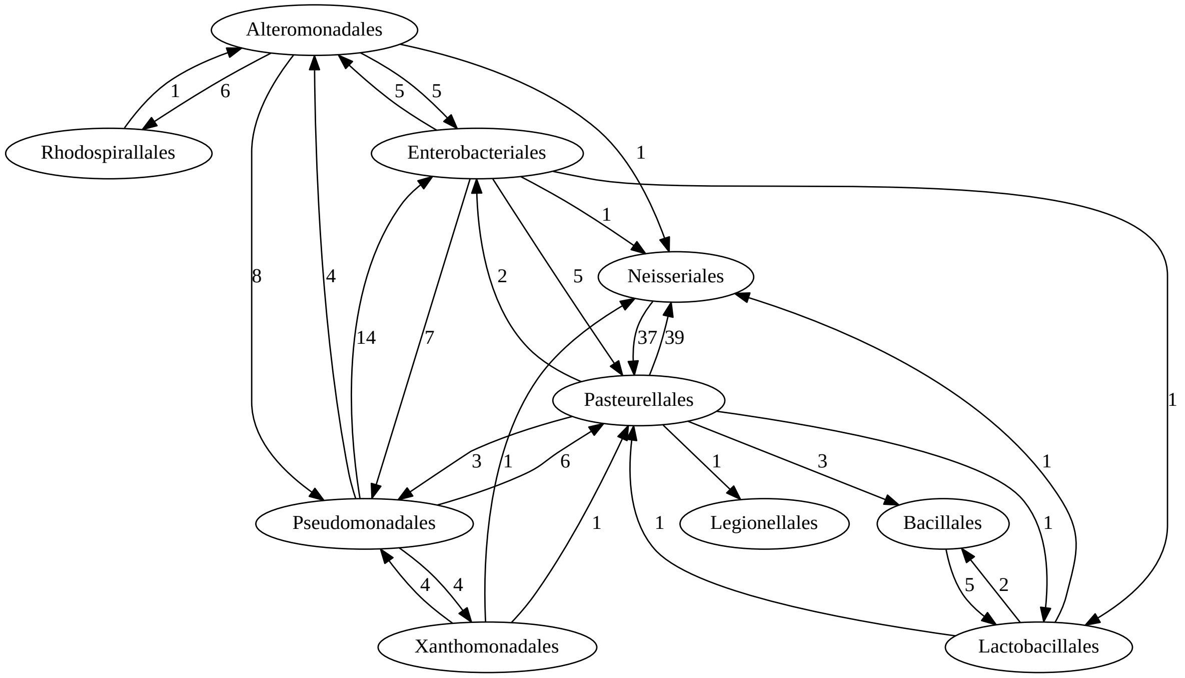

Supplementary Figure 18. LGT network for the BAC dataset, *k* = 35.

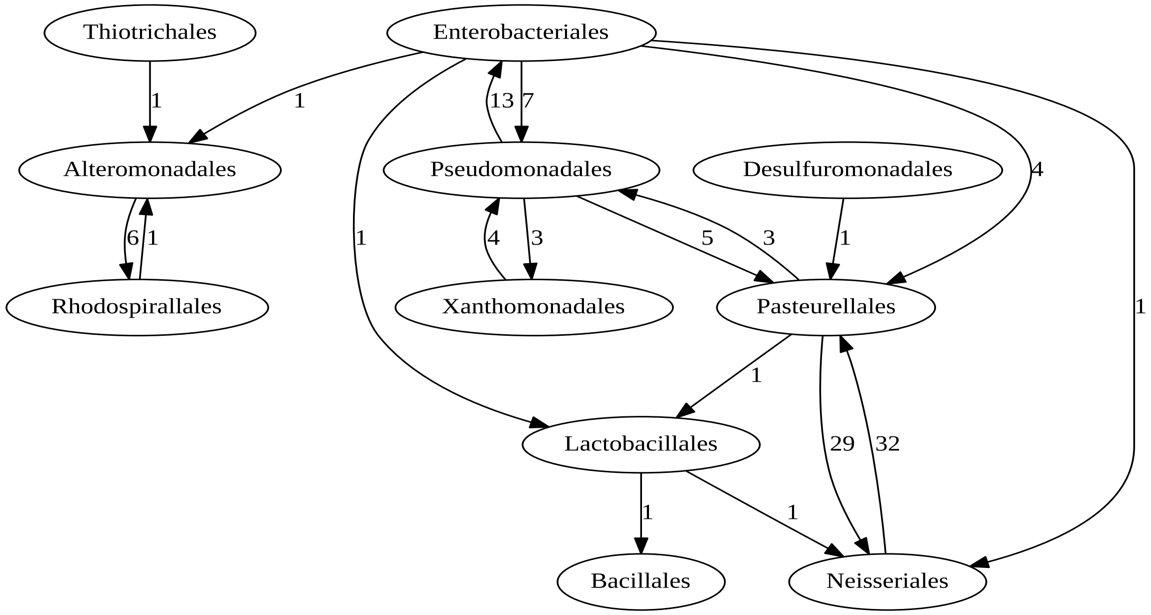

Supplementary Figure 19. LGT network for the BAC dataset, *k* = 40.

Supplementary Table 23. BAC dataset, *k* = 25; maximum clique as the GEC.

1	2	3	4	5	6	7
Xanthomonadales	Neisseriales	Rhodospirallales	Enterobacteriales	Pseudomonadales	Alteromonadales	Pasteurellales

Supplementary Table 24. BAC dataset, *k* = 25; maximal cliques as the GECs.

1	2	3	4	5	6	7
Pasteurellales	Alteromonadales	Rhodospirallales	Enterobacteriales	Xanthomonadales	Neisseriales	Pseudomonadales
Pasteurellales Alteromonadales Rhodospirallales Enterobacteriales Desulfuromonadales						
Pasteurellales Alteromonadales Thiotrichales Pseudomonadales						
Pasteurellales Alteromonadales Thiotrichales Clostridiales						
Pasteurellales Alteromonadales Clostridiales Desulfuromonadales						
Pasteurellales Alteromonadales Legionellales						
Pasteurellales Bacillales Enterobacteriales Pseudomonadales Neisseriales						
Pasteurellales Bacillales Enterobacteriales Pseudomonadales Lactobacillales						
Pasteurellales Bacillales Enterobacteriales Desulfuromonadales						
Pasteurellales Bacillales Clostridiales Desulfuromonadales						
Pasteurellales Bacillales Clostridiales Lactobacillales						
Rhizobales Pseudomonadales Bacillales						
Rhizobales Pseudomonadales Alteromonadales						
Thermoanaerobacteriales Clostridiales Lactobacillales Bacillales						
Mycoplasmatales Clostridiales Bacillales						

Supplementary Table 25. BAC dataset, *k* = 30; maximum clique as the GEC.

1	2	3	4
Neisseriales	Pasteurellales	Lactobacillales	Enterobacteriales

Supplementary Table 26. BAC dataset, *k* = 30; maximal cliques as the GECs.

1	2	3	4
Enterobacteriales	Pseudomonadales	Alteromonadales	Xanthomonadales
Enterobacteriales	Pseudomonadales	Pasteurellales	Lactobacillales
Enterobacteriales	Neisseriales	Xanthomonadales	Alteromonadales
Enterobacteriales	Neisseriales	Pasteurellales	Lactobacillales
Rhodospirallales Xanthomonadales Alteromonadales			
Clostridiales Bacillales Thermoanaerobacteriales			

Supplementary Table 27. BAC dataset, k = 35; maximum clique as the GEC.

1	2	3	4
Lactobacillales	Enterobacteriales	Neisseriales	Pasteurellales

Supplementary Table 28. BAC dataset, *k* = 35; maximal cliques as the GECs.

1	2	3	4
Pasteurellales	Enterobacteriales	Lactobacillales	Neisseriales
Pasteurellales Enterobacteriales Pseudomonadales			
Pasteurellales Xanthomonadales Pseudomonadales			
Pasteurellales Xanthomonadales Neisseriales			
Pasteurellales Bacillales Lactobacillales			
Alteromonadales Enterobacteriales Pseudomonadales			
Alteromonadales Enterobacteriales Neisseriales			

Supplementary Table 29. BAC dataset, *k* = 40; maximum clique as the GEC.

1	2	3	4
Neisseriales	Lactobacillales	Enterobacteriales Pasteurellales	

Supplementary Table 30. BAC dataset, *k* = 40; maximal cliques as the GECs.

1	2	3	4
Enterobacteriales	Pasteurellales	Neisseriales	Lactobacillales
Enterobacteriales	Pseudomonadales Pasteurellale		

Supplementary Table 31. Lateral genes inferred in BAC dataset, *k* = 25.

GI	LOCUS	Gene name	Coordinates	Recipient_genome	Donor_order
110342700	ABG68937.1	30S ribosomal protein S1	972554-974228	Escherichia_coli_536	Alteromonadales
110344676	ABG70913.1	transketolase 1	3096688-3098680	Escherichia_coli_536	Alteromonadales
110344682	ABG70919.1	S-adenosylmethionine synthetase	3104510-3105665	Escherichia_coli_536	Neisseriales
110344682	ABG70919.1	S-adenosylmethionine synthetase	3104510-3105665	Escherichia_coli_536	Alteromonadales
110344903	ABG71140.1	RNA polymerase sigma factor RpoD	3324732-3326574	Escherichia_coli_536	Neisseriales
110345147	ABG71384.1	30S ribosomal protein S19	3559897-3560176	Escherichia_coli_536	Alteromonadales
110345907	ABG72144.1	elongation factor Tu	4387901-4389086	Escherichia_coli_536	Alteromonadales
110345907	ABG72144.1	elongation factor Tu	4387901-4389086	Escherichia_coli_536	Pasteurellales
110345914	ABG72151.1	DNA-directed RNA polymerase beta chain	4393081-4397110	Escherichia_coli_536	Alteromonadales
115512253	ABJ00328.1	RpsA	965474-967148	Escherichia_coli_APEC_O1	Alteromonadales
115514288	ABJ02363.1	transketolase 1, thiamin-binding	3273504-3275496	Escherichia_coli_APEC_O1	Alteromonadales
115514628	ABJ02703.1	glutamate synthase small subunit	3647206-3651775	Escherichia_coli_APEC_O1	Alteromonadales
115514719	ABJ02794.1	30S ribosomal protein S19	3740972-3741251	Escherichia_coli_APEC_O1	Alteromonadales
115515368	ABJ03443.1	translation elongation factor EF-Tu	4497524-4498709	Escherichia_coli_APEC_O1	Alteromonadales
115515368	ABJ03443.1	translation elongation factor EF-Tu	4497524-4498709	Escherichia_coli_APEC_O1	Pasteurellales
115515374	ABJ03449.1	DNA-directed RNA polymerase beta chain protein RpoB	4502671-4506733	Escherichia_coli_APEC_O1	Alteromonadales
118423389	ABK89779.1	holliday junction DNA helicase, subunit B	947377-948424	Francisella_tularensis_subsp._novicida_U112	Pasteurellales
119765667	ABL98237.1	Glycine--tRNA ligase	23456-25526	Shewanella_amazonensis_SB2B	Enterobacteriales
119765702	ABL98272.1	phosphoglycerate mutase	65148-66690	Shewanella_amazonensis_SB2B	Enterobacteriales
119765847	ABL98417.1	DNA-directed RNA polymerase	238653-242682	Shewanella_amazonensis_SB2B	Rhodospirallales
119765847	ABL98417.1	DNA-directed RNA polymerase	238653-242682	Shewanella_amazonensis_SB2B	Neisseriales
119765847	ABL98417.1	DNA-directed RNA polymerase	238653-242682	Shewanella_amazonensis_SB2B	Enterobacteriales
119765848	ABL98418.1	DNA-directed RNA polymerase	242767-246982	Shewanella_amazonensis_SB2B	Rhodospirallales
119765848	ABL98418.1	DNA-directed RNA polymerase	242767-246982	Shewanella_amazonensis_SB2B	Enterobacteriales
119765848	ABL98418.1	DNA-directed RNA polymerase	242767-246982	Shewanella_amazonensis_SB2B	Pseudomonadales
119765857	ABL98427.1	LSU ribosomal protein L2P	253803-254628	Shewanella_amazonensis_SB2B	Pasteurellales
119765858	ABL98428.1	SSU ribosomal protein S19P	254643-254922	Shewanella_amazonensis_SB2B	Enterobacteriales
119765866	ABL98436.1	LSU ribosomal protein L5P	257716-258256	Shewanella_amazonensis_SB2B	Pseudomonadales
119765876	ABL98446.1	SSU ribosomal protein S13P	262637-262994	Shewanella_amazonensis_SB2B	Enterobacteriales
119765878	ABL98448.1	SSU ribosomal protein S4P	263432-264053	Shewanella_amazonensis_SB2B	Enterobacteriales
119766016	ABL98586.1	pyruvate dehydrogenase, E1 component	453822-456519	Shewanella_amazonensis_SB2B	Enterobacteriales
119766023	ABL98593.1	aconitase	470281-472879	Shewanella_amazonensis_SB2B	Enterobacteriales
119766066	ABL98636.1	3-octaprenyl-4hydroxybenzoate decarboxylase	522663-524145	Shewanella_amazonensis_SB2B	Neisseriales
119766154	ABL98724.1	Excinuclease ABC subunit A	631611-634440	Shewanella_amazonensis_SB2B	Neisseriales
119766154	ABL98724.1	Excinuclease ABC subunit A	631611-634440	Shewanella_amazonensis_SB2B	Enterobacteriales
119766427	ABL98997.1	valyl-tRNA synthetase	973084-975946	Shewanella_amazonensis_SB2B	Enterobacteriales
119766460	ABL99030.1	RNA polymerase, sigma 70 subunit, RpoD	1006838-1008701	Shewanella_amazonensis_SB2B	Desulfuromonadales
119766460	ABL99030.1	RNA polymerase, sigma 70 subunit, RpoD	1006838-1008701	Shewanella_amazonensis_SB2B	Enterobacteriales
119766515	ABL99085.1	GTP-binding protein LepA	1066894-1068685	Shewanella_amazonensis_SB2B	Enterobacteriales
119766561	ABL99131.1	Isoleucyl-tRNA synthetase	1116413-1119236	Shewanella_amazonensis_SB2B	Enterobacteriales
119766591	ABL99161.1	membrane protease FtsH catalytic subunit	1153138-1155091	Shewanella_amazonensis_SB2B	Enterobacteriales
119766683	ABL99253.1	recA protein	1265501-1266569	Shewanella_amazonensis_SB2B	Enterobacteriales
119766773	ABL99343.1	2,3,4,5-tetrahydropyridine-2,6-dicarboxylate N-succinyltransferase	1384756-1385581	Shewanella_amazonensis_SB2B	Neisseriales
119766776	ABL99346.1	SSU ribosomal protein S2P	1389251-1390028	Shewanella_amazonensis_SB2B	Enterobacteriales
119766777	ABL99347.1	translation elongation factor Ts (EF-Ts)	1390155-1391007	Shewanella_amazonensis_SB2B	Thiotrichales
119766778	ABL99348.1	uridylate kinase	1391075-1391801	Shewanella_amazonensis_SB2B	Enterobacteriales
119766858	ABL99428.1	cysteinyl-tRNA synthetase	1501834-1503214	Shewanella_amazonensis_SB2B	Enterobacteriales
119767066	ABL99636.1	succinyl-CoA synthetase (ADP-forming) beta subunit	1740652-1741819	Shewanella_amazonensis_SB2B	Pseudomonadales
119767256	ABL99826.1	ATP-dependent helicase HrpA	1981025-1984895	Shewanella_amazonensis_SB2B	Enterobacteriales
119767320	ABL99890.1	asparaginyl-tRNA synthetase	2058083-2059484	Shewanella_amazonensis_SB2B	Enterobacteriales
119767371	ABL99941.1	SSU ribosomal protein S1P	2126184-2127852	Shewanella_amazonensis_SB2B	Enterobacteriales
119767403	ABL99973.1	LSU ribosomal protein L20P	2162345-2162705	Shewanella_amazonensis_SB2B	Pseudomonadales
119767539	ABM00110.1	AMP-binding family protein	2329002-2330724	Shewanella_amazonensis_SB2B	Desulfuromonadales
119767690	ABM00261.1	bacterial translation initiation factor 1 (bIF-1)	2490504-2490723	Shewanella_amazonensis_SB2B	Enterobacteriales
119767706	ABM00277.1	oxidoreductase, FAD-binding, putative	2505973-2508997	Shewanella_amazonensis_SB2B	Enterobacteriales
119768143	ABM00714.1	chaperone protein DnaK	3015982-3017896	Shewanella_amazonensis_SB2B	Enterobacteriales
119768267	ABM00838.1	methyl-accepting chemotaxis sensory transducer	3155681-3157802	Shewanella_amazonensis_SB2B	Desulfuromonadales
119768319	ABM00890.1	ATP-dependent RNA helicase, DEAD box family	3215920-3217522	Shewanella_amazonensis_SB2B	Rhodospirallales
119768392	ABM00963.1	methionine adenosyltransferase	3301223-3302375	Shewanella_amazonensis_SB2B	Enterobacteriales
119768703	ABM01274.1	LSU ribosomal protein L13P	3649329-3649758	Shewanella_amazonensis_SB2B	Enterobacteriales
119768707	ABM01278.1	UDP-N-acetylglucosamine 1-carboxyvinyltransferase	3654273-3655530	Shewanella_amazonensis_SB2B	Enterobacteriales
119768756	ABM01327.1	chaperonin GroEL	3704503-3706141	Shewanella_amazonensis_SB2B	Neisseriales
119768761	ABM01332.1	multi-sensor hybrid histidine kinase	3711338-3715049	Shewanella_amazonensis_SB2B	Desulfuromonadales
119768849	ABM01420.1	transcription termination factor Rho	3808939-3810202	Shewanella_amazonensis_SB2B	Enterobacteriales
119768966	ABM01537.1	ornithine decarboxylase	3960624-3962787	Shewanella_amazonensis_SB2B	Enterobacteriales
119768981	ABM01552.1	conserved hypothetical Fe-S oxidoreductase	3974615-3977036	Shewanella_amazonensis_SB2B	Enterobacteriales
119768999	ABM01570.1	3-dehydroquinate synthase	3993163-3994243	Shewanella_amazonensis_SB2B	Enterobacteriales
119769271	ABM01842.1	glutamine--fructose-6-phosphate transaminase	4287695-4289525	Shewanella_amazonensis_SB2B	Enterobacteriales
119769278	ABM01849.1	Sodium-transporting two-sector ATPase	4297037-4298579	Shewanella_amazonensis_SB2B	Enterobacteriales
120615077	ABM45678.1	GTP-binding protein TypA/BipA	320733-322560	Bartonella_bacilliformis_KC583	Bacillales
120865869	CAM09602.1	adenylosuccinate lyase	291190-292561	Neisseria_meningitidis_serogroup_C_FAM18	Xanthomonadales
120865900	CAM09636.1	putative leucyl-tRNA synthetase	327942-330573	Neisseria_meningitidis_serogroup_C_FAM18	Xanthomonadales
120866055	CAM09793.1	putative chaperone protein	523484-525413	Neisseria_meningitidis_serogroup_C_FAM18	Xanthomonadales
120866270	CAM10011.1	putative pilus retraction protein	740246-741359	Neisseria_meningitidis_serogroup_C_FAM18	Xanthomonadales
120866475	CAM10222.1	putative transposase (pseudogene)	951840-952014	Neisseria_meningitidis_serogroup_C_FAM18	Pasteurellales
120866532	CAM10282.1	putative serine hydroxymethyltransferase	1006811-1008062	Neisseria_meningitidis_serogroup_C_FAM18	Bacillales
120866639	CAM10390.1	putative ABC-transporter ATP-binding protein	1118359-1119988	Neisseria_meningitidis_serogroup_C_FAM18	Alteromonadales
120866721	CAM10474.1	putative transcriptional regulator	1217953-1218361	Neisseria_meningitidis_serogroup_C_FAM18	Pasteurellales
120866722	CAM10475.1	alcohol dehydrogenase class-III	1218481-1219618	Neisseria_meningitidis_serogroup_C_FAM18	Pasteurellales
120866723	CAM10476.1	esterase D	1219626-1220454	Neisseria_meningitidis_serogroup_C_FAM18	Pasteurellales
120866787	CAM10540.1	conserved hypothetical protein	1297476-1299711	Neisseria_meningitidis_serogroup_C_FAM18	Pasteurellales
120866788	CAM10541.1	putative type III restriction/modification system enzyme	1299658-1302475	Neisseria_meningitidis_serogroup_C_FAM18	Pasteurellales
120866861	CAM10620.1	ClpB protein	1410211-1412791	Neisseria_meningitidis_serogroup_C_FAM18	Xanthomonadales
120866902	CAM10661.1	putative virulence associated protein	1461458-1463403	Neisseria_meningitidis_serogroup_C_FAM18	Pasteurellales
120866913	CAM10672.1	RNA polymerase sigma factor	1477005-1478982	Neisseria_meningitidis_serogroup_C_FAM18	Enterobacteriales
120867091	CAM10857.1	conserved hypothetical protein	1705208-1706003	Neisseria_meningitidis_serogroup_C_FAM18	Pasteurellales
120867294	CAM11065.1	glycyl-tRNA synthetase alpha chain	1935148-1936045	Neisseria_meningitidis_serogroup_C_FAM18	Xanthomonadales
125999534	ABN63609.1	LSU ribosomal protein L17P	4864669-4865065	Shewanella_baltica_OS155	Pasteurellales
125999556	ABN63631.1	SSU ribosomal protein S19P	4875255-4875534	Shewanella_baltica_OS155	Enterobacteriales
126386404	ABO10902.1	50S ribosomal protein L33	486868-487024	Acinetobacter_baumannii_ATCC_17978	Pasteurellales
126386831	ABO11329.1	twitching motility protein	1037762-1038800	Acinetobacter_baumannii_ATCC_17978	Alteromonadales
126388056	ABO12554.1	aconitate hydratase 2	2480061-2480808	Acinetobacter_baumannii_ATCC_17978	Alteromonadales
126388458	ABO12956.1	putative tetrahydropyridine-2-carboxylate N-succinyltransferase	2937401-2938223	Acinetobacter_baumannii_ATCC_17978	Rhodospirallales
148290911	CAL85047.1	putative 50S ribosomal protein L7ae	3712425-3712665	Clostridium_botulinum_A_str._ATCC_3502	Thermoanaerobacteriales
148715301	ABQ97511.1	elongation factor EF-2	14655-16758	Haemophilus_influenzae_PittEE	Pseudomonadales
148715302	ABQ97512.1	elongation factor Tu	16822-18007	Haemophilus_influenzae_PittEE	Bacillales
148715343	ABQ97553.1	chaperonin GroEL	49781-51428	Haemophilus_influenzae_PittEE	Thiotrichales
148715418	ABQ97628.1	ATP-dependent proteinase	129439-131851	Haemophilus_influenzae_PittEE	Alteromonadales
148715424	ABQ97634.1	thymidylate kinase	136571-137204	Haemophilus_influenzae_PittEE	Neisseriales
148715425	ABQ97635.1	DNA polymerase III subunit delta	137200-138184	Haemophilus_influenzae_PittEE	Neisseriales
148715645	ABQ97855.1	preprotein translocase subunit SecF	360900-362742	Haemophilus_influenzae_PittEE	Enterobacteriales
148715710	ABQ97920.1	alcohol dehydrogenase class III	458047-459184	Haemophilus_influenzae_PittEE	Neisseriales
148715711	ABQ97921.1	esterase	459192-460020	Haemophilus_influenzae_PittEE	Neisseriales
148716007	ABQ98217.1	predicted P-loop ATPase fused to an acetyltransferase	828122-830093	Haemophilus_influenzae_PittEE	Xanthomonadales
148716173	ABQ98383.1	insertion element IS1016 transposase	1007261-1007573	Haemophilus_influenzae_PittEE	Neisseriales
148716174	ABQ98384.1	D-alanyl-D-alanine carboxypeptidase/endopeptidase	1007538-1007910	Haemophilus_influenzae_PittEE	Neisseriales
148716233	ABQ98443.1	biotin synthesis protein	1070871-1071654	Haemophilus_influenzae_PittEE	Neisseriales
148716234	ABQ98444.1	dithiobiotin synthetase	1071641-1072289	Haemophilus_influenzae_PittEE	Neisseriales
148716235	ABQ98445.1	8-amino-7-oxononanoate synthase	1072298-1073441	Haemophilus_influenzae_PittEE	Neisseriales
148716487	ABQ98697.1	putative type III restriction/modification system modification methylase	1343864-1346006	Haemophilus_influenzae_PittEE	Neisseriales

148716580	ABQ98790.1	phosphopyruvate hydratase	1456710-1458237	Haemophilus_influenzae_PittEE	Neisseriales
148716877	ABQ99087.1	putative LysR-family transcriptional regulator	1768573-1769245	Haemophilus_influenzae_PittEE	Neisseriales
148718565	ABQ99692.1	esterase	655421-656249	Haemophilus_influenzae_PittGG	Neisseriales
148718566	ABQ99693.1	alcohol dehydrogenase class III	656257-657394	Haemophilus_influenzae_PittGG	Neisseriales
148718567	ABQ99694.1	putative HTH-type transcriptional regulator	657516-657924	Haemophilus_influenzae_PittGG	Neisseriales
148718625	ABQ99752.1	queuine tRNA-ribosyltransferase	728126-729275	Haemophilus_influenzae_PittGG	Pseudomonadales
148718631	ABQ99758.1	hypothetical protein	744413-744824	Haemophilus_influenzae_PittGG	Neisseriales
148718837	ABQ99964.1	translation-associated GTPase	938893-939985	Haemophilus_influenzae_PittGG	Pseudomonadales
148718894	ABR00022.1	DNA polymerase III subunit delta'	1001350-1003522	Haemophilus_influenzae_PittGG	Neisseriales
148718947	ABR00075.1	DNA-directed RNA polymerase subunit beta'	1054348-1058599	Haemophilus_influenzae_PittGG	Thiotrichales
148719030	ABR00158.1	recombination regulator RecX	1143040-1143859	Haemophilus_influenzae_PittGG	Neisseriales
148719049	ABR00177.1	elongation factor Tu	1167433-1168618	Haemophilus_influenzae_PittGG	Bacillales
148719462	ABR00590.1	hypothetical protein	1648390-1648990	Haemophilus_influenzae_PittGG	Neisseriales
148719463	ABR00591.1	putative type III restriction-modification system HindVIP enzyme res	1649001-1651158	Haemophilus_influenzae_PittGG	Neisseriales
148719464	ABR00592.1	twin-argninine leader-binding protein DmsD	1651147-1653322	Haemophilus_influenzae_PittGG	Neisseriales
148719611	ABR00739.1	predicted membrane protein	1820138-1821155	Haemophilus_influenzae_PittGG	Neisseriales
148719623	ABR00751.1	50S ribosomal protein L25	1835808-1836096	Haemophilus_influenzae_PittGG	Xanthomonadales
148719668	ABR00796.1	arginyl-tRNA synthetase	1885130-1886864	Haemophilus_influenzae_PittGG	Enterobacteriales
148719669	ABR00797.1	hypothetical protein	0-1887192	Haemophilus_influenzae_PittGG	Xanthomonadales
148719669	ABR00797.1	hypothetical protein	0-1887192	Haemophilus_influenzae_PittGG	Desulfuromonadales
148719669	ABR00797.1	hypothetical protein	0-1887192	Haemophilus_influenzae_PittGG	Bacillales
148719669	ABR00797.1	hypothetical protein	0-1887192	Haemophilus_influenzae_PittGG	Clostridiales
148719669	ABR00797.1	hypothetical protein	0-1887192	Haemophilus_influenzae_PittGG	Rhodospirallales
148719669	ABR00797.1	hypothetical protein	0-1887192	Haemophilus_influenzae_PittGG	Neisseriales
148719669	ABR00797.1	hypothetical protein	0-1887192	Haemophilus_influenzae_PittGG	Alteromonadales
148719669	ABR00797.1	hypothetical protein	0-1887192	Haemophilus_influenzae_PittGG	Enterobacteriales
148719669	ABR00797.1	hypothetical protein	0-1887192	Haemophilus_influenzae_PittGG	Legionellales
148719669	ABR00797.1	hypothetical protein	0-1887192	Haemophilus_influenzae_PittGG	Pseudomonadales
148719669	ABR00797.1	hypothetical protein	0-1887192	Haemophilus_influenzae_PittGG	Thiotrichales
15022823	AAK77993.1	DNA gyrase (topoisomerase II) B subunit	4843-6757	Clostridium_acetobutylicum_ATCC_824	Desulfuromonadales
15023227	AAK78358.1	Glutamine ABC transporter, ATP-binding protein (gene glnQ)	440982-441714	Clostridium_acetobutylicum_ATCC_824	Lactobacillales
15023365	AAK78483.1	Excinuclease ABC subunit A (ATP-ase), (uvrA)	577259-580079	Clostridium_acetobutylicum_ATCC_824	Thiotrichales
15023863	AAK78935.1	ATPase with chaperone activity, two ATP-binding domains	1097624-1100222	Clostridium_acetobutylicum_ATCC_824	Thermoanaerobacteriales
15025267	AAK80221.1	Glycine hydroxymethyltransferase	2368161-2369397	Clostridium_acetobutylicum_ATCC_824	Thermoanaerobacteriales
15025373	AAK80318.1	Threonyl-tRNA synthetase	2471595-2473509	Clostridium_acetobutylicum_ATCC_824	Thermoanaerobacteriales
15026237	AAK81106.1	Acetolactate synthase large subunit	3303325-3304990	Clostridium_acetobutylicum_ATCC_824	Thermoanaerobacteriales
151363269	ABS06269.1	urocanate hydratase	117635-119303	Shewanella_baltica_OS185	Xanthomonadales
151363353	ABS06353.1	translation elongation factor Tu	227718-228903	Shewanella_baltica_OS185	Enterobacteriales
151363365	ABS06365.1	translation elongation factor Tu	244481-245666	Shewanella_baltica_OS185	Enterobacteriales
151363371	ABS06371.1	ribosomal protein S19	248762-249041	Shewanella_baltica_OS185	Enterobacteriales
151363722	ABS06722.1	excinuclease ABC, A subunit	636615-639498	Shewanella_baltica_OS185	Xanthomonadales
151364126	ABS07126.1	methionine synthase	1162542-1166277	Shewanella_baltica_OS185	Enterobacteriales
151364273	ABS07273.1	isoleucyl-tRNA synthetase	1333718-1336541	Shewanella_baltica_OS185	Pasteurellales
151364610	ABS07610.1	DNA polymerase III, alpha subunit	1760234-1763708	Shewanella_baltica_OS185	Pasteurellales
151365076	ABS08076.1	ATP-dependent helicase HrpA	2306890-2310772	Shewanella_baltica_OS185	Enterobacteriales
151365361	ABS08361.1	threonyl-tRNA synthetase	2646132-2648061	Shewanella_baltica_OS185	Pasteurellales
151365375	ABS08375.1	ABC transporter related	2662988-2664599	Shewanella_baltica_OS185	Neisseriales
151366668	ABS09668.1	Methionine adenosyltransferase	4214011-4215163	Shewanella_baltica_OS185	Enterobacteriales
151367366	ABS10366.1	transporter, hydrophobe/amphiphile efflux-1 (HAE1) family	5064062-5067197	Shewanella_baltica_OS185	Xanthomonadales
152929822	ABS35322.1	DNA-directed RNA polymerase, beta subunit	3692335-3696034	Clostridium_botulinum_A_str._ATCC_19397	Thermoanaerobacteriales
154350382	ABS72461.1	SerS	20660-21938	Bacillus_amyloliquefaciens_FZB42	Thermoanaerobacteriales
154350476	ABS72555.1	RpoB	122625-126216	Bacillus_amyloliquefaciens_FZB42	Lactobacillales
154350482	ABS72561.1	TufA	133587-134778	Bacillus_amyloliquefaciens_FZB42	Lactobacillales
154350488	ABS72567.1	RplB	138042-138876	Bacillus_amyloliquefaciens_FZB42	Lactobacillales
156617158	AAX87127.2	transposon Tn3 resolvase	128087-128681	Haemophilus_influenzae_86-028NP	Pseudomonadales
156617159	ABU87507.1	beta-lactamase TEM precursor	128728-129589	Haemophilus_influenzae_86-028NP	Pseudomonadales
160858834	ABX47368.1	translation elongation factor Tu	230933-232118	Shewanella_baltica_OS195	Enterobacteriales
160858834	ABX47368.1	translation elongation factor Tu	230933-232118	Shewanella_baltica_OS195	Pseudomonadales
160858846	ABX47380.1	translation elongation factor Tu	247697-248882	Shewanella_baltica_OS195	Enterobacteriales
160858852	ABX47386.1	ribosomal protein S19	251978-252257	Shewanella_baltica_OS195	Enterobacteriales
160859372	ABX47906.1	UDP-N-acetylglucosamine 1-carboxyvinyltransferase	876312-877572	Shewanella_baltica_OS195	Clostridiales
160859798	ABX48332.1	isoleucyl-tRNA synthetase	1369438-1372261	Shewanella_baltica_OS195	Pasteurellales
160859927	ABX48461.1	signal recognition particle protein	1524088-1525462	Shewanella_baltica_OS195	Pasteurellales
160860964	ABX49498.1	threonyl-tRNA synthetase	2747805-2749734	Shewanella_baltica_OS195	Pseudomonadales
160862207	ABX50741.1	methyl-accepting chemotaxis sensory transducer	4208449-4210081	Shewanella_baltica_OS195	Xanthomonadales
160863008	ABX51542.1	transporter, hydrophobe/amphiphile efflux-1 (HAE1) family	5179876-5183011	Shewanella_baltica_OS195	Xanthomonadales
161017008	CAK00566.1	heat shock protein 70 DnaK	84783-86676	Bartonella_tribocorum_CIP_105476	Alteromonadales
161594783	ABX72443.1	hypothetical inner membrane protein	245153-246170	Neisseria_meningitidis_053442	Pasteurellales
161595265	ABX72925.1	twitching motility protein	737629-738760	Neisseria_meningitidis_053442	Xanthomonadales
161595295	ABX72955.1	ATP-dependent zinc metallopeptidase	767931-769899	Neisseria_meningitidis_053442	Xanthomonadales
161595434	ABX73094.1	conserved hypothetical protein	918891-919065	Neisseria_meningitidis_053442	Pasteurellales
161595729	ABX73389.1	transcriptional regulator, MerR family	1209345-1209753	Neisseria_meningitidis_053442	Pasteurellales
161595730	ABX73390.1	alcohol dehydrogenase class-III	1209873-1211010	Neisseria_meningitidis_053442	Pasteurellales
161595731	ABX73391.1	esterase D	1211117-1211846	Neisseria_meningitidis_053442	Pasteurellales
161595799	ABX73459.1	conserved hypothetical protein	1288240-1288777	Neisseria_meningitidis_053442	Pasteurellales
161595800	ABX73460.1	type III restriction/modification system modification methylase	1289069-1290185	Neisseria_meningitidis_053442	Pasteurellales
161595801	ABX73461.1	type III restriction/modification system enzyme	1290174-1292958	Neisseria_meningitidis_053442	Pasteurellales
161595952	ABX73612.1	RNA polymerase primary sigma factor	1467469-1469398	Neisseria_meningitidis_053442	Enterobacteriales
161596155	ABX73815.1	8-amino-7-oxonoanoate synthase	1698571-1699606	Neisseria_meningitidis_053442	Pasteurellales
161596367	ABX74027.1	NADH dehydrogenase I chain I	1944572-1945052	Neisseria_meningitidis_053442	Xanthomonadales
161596397	ABX74057.1	3-oxoacyl-[acyl-carrier-protein] synthase II	1973837-1975085	Neisseria_meningitidis_053442	Xanthomonadales
169150959	CAO99574.1	membrane-bound ATP synthase, F1 sector, beta-subunit	159399-160794	Acinetobacter_baumannii_SDF	Neisseriales
169151154	CAO99826.2	50S ribosomal protein L5	403463-404000	Acinetobacter_baumannii_SDF	Alteromonadales
169151306	CAP00010.1	prolyl-tRNA synthetase	581237-582953	Acinetobacter_baumannii_SDF	Pasteurellales
169151475	CAP00226.1	DNA gyrase, subunit A, type II topoisomerase	799372-802087	Acinetobacter_baumannii_SDF	Pasteurellales
169153275	CAP02380.1	3-isopropylmalate dehydratase (isomerase), subunit with LeuD	2851559-2853089	Acinetobacter_baumannii_SDF	Thiotrichales
169153333	CAP02444.1	2-octaprenylphenol hydroxylase of ubiquinone biosynthetic pathway	2925962-2927582	Acinetobacter_baumannii_SDF	Alteromonadales
169153385	CAP02512.1	DNA-directed RNA polymerase beta' chain (Transcriptase beta' chain) (RNA polymerase beta' subunit)	2991839-2996033	Acinetobacter_baumannii_SDF	Pasteurellales
169153386	CAP02513.1	DNA-directed RNA polymerase beta chain (Transcriptase beta chain) (RNA polymerase beta subunit)	2996119-3000208	Acinetobacter_baumannii_SDF	Pasteurellales
169406026	ACA54437.1	carbamoyl-phosphate synthase, large subunit	2025568-2028775	Clostridium_botulinum_A3_str._Loch_Maree	Thermoanaerobacteriales
169407641	ACA56052.1	DNA gyrase, B subunit	4407-6321	Clostridium_botulinum_A3_str._Loch_Maree	Bacillales
169408286	ACA56697.1	aldehyde-alcohol dehydrogenase	409177-411766	Clostridium_botulinum_A3_str._Loch_Maree	Alteromonadales
183207945	ACC55343.1	Molecular chaperone	31975-33916	Acinetobacter_baumannii_ACICU	Pasteurellales
183208092	ACC55490.1	F0F1-type ATP synthase, beta subunit	199518-200913	Acinetobacter_baumannii_ACICU	Neisseriales
183208210	ACC55608.1	truncated elongation factor EF-Tu	321236-321839	Acinetobacter_baumannii_ACICU	Alteromonadales
183208371	ACC55769.1	putative ribosomal protein L33	505859-506015	Acinetobacter_baumannii_ACICU	Pasteurellales
183208731	ACC56129.1	Translation elongation factor (GTPases)	911034-913173	Acinetobacter_baumannii_ACICU	Pasteurellales
183208732	ACC56130.1	truncated elongation factor EF-Tu	913267-913870	Acinetobacter_baumannii_ACICU	Alteromonadales
183209435	ACC56833.1	predicted GTPase, probable translation factor	1633038-1634130	Acinetobacter_baumannii_ACICU	Pasteurellales
183209848	ACC57246.1	Catalase	2064156-2065677	Acinetobacter_baumannii_ACICU	Pasteurellales
183210242	ACC57640.1	Aconitase B	2452990-2455630	Acinetobacter_baumannii_ACICU	Alteromonadales
183210822	ACC58220.1	Chaperonin GroEL (HSP60 family)	3074056-3075700	Acinetobacter_baumannii_ACICU	Pasteurellales
189422319	ACD96717.1	ABC transporter related	3225885-3227523	Geobacter_lovleyi_SZ	Bacillales
193076046	ABO10638.2	membrane-bound ATP synthase F1 sector, beta-subunit	176286-177681	Acinetobacter_baumannii_ATCC_17978	Neisseriales
193076137	ABO10750.2	protein chain elongation factor EF-Tu	300452-301643	Acinetobacter_baumannii_ATCC_17978	Alteromonadales
193076477	ABO11120.2	Resolvase	795673-796411	Acinetobacter_baumannii_ATCC_17978	Xanthomonadales
193076621	ABO11300.2	protein chain elongation factor EF-G GTP-binding	1007989-1010128	Acinetobacter_baumannii_ATCC_17978	Pasteurellales
193076622	ABO11301.2	protein chain elongation factor EF-Tu	1010222-1011371	Acinetobacter_baumannii_ATCC_17978	Alteromonadales
193077132	ABO11909.2	putative GTP-binding protein	1726152-1727244	Acinetobacter_baumannii_ATCC_17978	Enterobacteriales
193078137	ABO13080.2	chaperone Hsp60	3088347-3089982	Acinetobacter_baumannii_ATCC_17978	Pasteurellales

193078184	ABO13131.2	succinyl-CoA synthetase beta chain	3154409-3155576	Acinetobacter_baumannii_ATCC_17978	Alteromonadales
193933309	ACF29133.1	queuine tRNA-ribosyltransferase	367326-368442	Neisseria_gonorrhoeae_NCCP11945	Xanthomonadales
193933661	ACF29485.1	Thymidylate kinase	656782-656947	Neisseria_gonorrhoeae_NCCP11945	Pasteurellales
193933662	ACF29486.1	autotransported protein Lav	656933-657056	Neisseria_gonorrhoeae_NCCP11945	Pasteurellales
193933664	ACF29488.1	putative virulence associated protein	657922-659014	Neisseria_gonorrhoeae_NCCP11945	Pasteurellales
193933979	ACF29803.1	Conserved hypothetical protein	922921-923776	Neisseria_gonorrhoeae_NCCP11945	Pasteurellales
193934125	ACF29949.1	Twin-argninine leader-binding protein DmsD	1041135-1042509	Neisseria_gonorrhoeae_NCCP11945	Pasteurellales
193934126	ACF29950.1	RmsR	1042498-1045273	Neisseria_gonorrhoeae_NCCP11945	Pasteurellales
193934166	ACF29990.1	putative MerR-family transcriptional regulator	1083783-1084191	Neisseria_gonorrhoeae_NCCP11945	Pasteurellales
193934167	ACF29991.1	alcohol dehydrogenase class-III	1084691-1085042	Neisseria_gonorrhoeae_NCCP11945	Pasteurellales
193934169	ACF29993.1	putative esterase	1085459-1086287	Neisseria_gonorrhoeae_NCCP11945	Pasteurellales
193934572	ACF30396.1	Biotin synthesis protein BioC, putative	1450686-1451469	Neisseria_gonorrhoeae_NCCP11945	Pasteurellales
193934929	ACF30753.1	NADH dehydrogenase I chain I	1778138-1778618	Neisseria_gonorrhoeae_NCCP11945	Xanthomonadales
193934986	ACF30810.1	PetB	1832224-1833574	Neisseria_gonorrhoeae_NCCP11945	Alteromonadales
193935106	ACF30930.1	glutamyl-tRNA synthetase	1928570-1929965	Neisseria_gonorrhoeae_NCCP11945	Xanthomonadales
193935199	ACF31023.1	DNA-directed RNA polymerase beta' subunit	2019695-2023871	Neisseria_gonorrhoeae_NCCP11945	Xanthomonadales
193935199	ACF31023.1	DNA-directed RNA polymerase beta' subunit	2019695-2023871	Neisseria_gonorrhoeae_NCCP11945	Enterobacteriales
193935208	ACF31032.1	elongation factor EF-2	2027915-2030021	Neisseria_gonorrhoeae_NCCP11945	Enterobacteriales
193935288	ACF31112.1	KatA	2106850-2108365	Neisseria_gonorrhoeae_NCCP11945	Pasteurellales
193935401	ACF31225.1	ATP synthase subunit B	2204127-2205525	Neisseria_gonorrhoeae_NCCP11945	Pseudomonadales
197085823	ACH37094.1	efflux pump, RND family, inner membrane protein, AcrB/AcrD/AcrF family	74970-78072	Geobacter_bemidjiensis_Bem	Enterobacteriales
197089415	ACH40686.1	RNA polymerase sigma-70 factor RpoD	4220855-4222610	Geobacter_bemidjiensis_Bem	Alteromonadales
21107741	AAM36428.1	5-methyltetrahydrofolate-homocysteine methyltransferase	1800284-1803038	Xanthomonas_axonopodis_pv._citri_str._306	Alteromonadales
21110182	AAM38630.1	RNA polymerase sigma-70 factor	4456171-4458049	Xanthomonas_axonopodis_pv._citri_str._306	Alteromonadales
21112585	AAM40807.1	5-methyltetrahydrofolate-homocysteine methyltransferase	1765112-1767869	Xanthomonas_campestris_pv._campestris_str._ATCC_33913	Alteromonadales
21115012	AAM42993.1	RNA polymerase sigma-70 factor	4442014-4443889	Xanthomonas_campestris_pv._campestris_str._ATCC_33913	Alteromonadales
213054578	ACJ39480.1	chaperone protein DnaK	47618-49559	Acinetobacter_baumannii_AB0057	Pasteurellales
213054722	ACJ39624.1	ATP synthase F1, beta subunit	210037-211432	Acinetobacter_baumannii_AB0057	Neisseriales
213054790	ACJ39692.1	NTP-binding protein	285181-285691	Acinetobacter_baumannii_AB0057	Xanthomonadales
213054792	ACJ39694.1	insertion sequence IS6100	285644-286466	Acinetobacter_baumannii_AB0057	Xanthomonadales
213054793	ACJ39695.1	hypothetical protein	286596-286713	Acinetobacter_baumannii_AB0057	Enterobacteriales
213054795	ACJ39697.1	mercuric reductase	287615-289301	Acinetobacter_baumannii_AB0057	Enterobacteriales
213054796	ACJ39698.1	mercuric resistance protein MerC	289339-289765	Acinetobacter_baumannii_AB0057	Enterobacteriales
213054797	ACJ39699.1	tetracycline repressor protein, class A	291387-292065	Acinetobacter_baumannii_AB0057	Enterobacteriales
213054798	ACJ39700.1	hypothetical protein	292083-292254	Acinetobacter_baumannii_AB0057	Enterobacteriales
213054799	ACJ39701.1	tetracycline resistance protein, class A	292215-293343	Acinetobacter_baumannii_AB0057	Enterobacteriales
213054800	ACJ39702.1	regulator protein PecM	293374-294259	Acinetobacter_baumannii_AB0057	Enterobacteriales
213054805	ACJ39707.1	transposase Tn3	298835-301841	Acinetobacter_baumannii_AB0057	Pasteurellales
213054806	ACJ39708.1	transposon Tn3 resolvase	302003-302561	Acinetobacter_baumannii_AB0057	Pasteurellales
213054807	ACJ39709.1	beta-lactamase TEM	302743-303604	Acinetobacter_baumannii_AB0057	Pasteurellales
213054886	ACJ39788.1	translation elongation factor Tu	383497-384688	Acinetobacter_baumannii_AB0057	Alteromonadales
213055049	ACJ39951.1	ribosomal protein L33	568494-568650	Acinetobacter_baumannii_AB0057	Pasteurellales
213055803	ACJ40705.1	translation elongation factor G	971985-974124	Acinetobacter_baumannii_AB0057	Pasteurellales
213055804	ACJ40706.1	translation elongation factor Tu	974218-975409	Acinetobacter_baumannii_AB0057	Alteromonadales
213055962	ACJ40864.1	lysyl-tRNA synthetase	1146158-1147721	Acinetobacter_baumannii_AB0057	Alteromonadales
213056196	ACJ41098.1	GTP-binding protein YchF	1805516-1806608	Acinetobacter_baumannii_AB0057	Pasteurellales
213057509	ACJ42411.1	chaperonin GroL	3176050-3177685	Acinetobacter_baumannii_AB0057	Pasteurellales
213057671	ACJ42573.1	aconitate hydratase 2	2547079-2549719	Acinetobacter_baumannii_AB0057	Alteromonadales
213986220	ACJ56519.1	DNA-directed RNA polymerase, beta subunit	3460408-3464497	Acinetobacter_baumannii_AB307-0294	Alteromonadales
213986220	ACJ56519.1	DNA-directed RNA polymerase, beta subunit	3460408-3464497	Acinetobacter_baumannii_AB307-0294	Pasteurellales
213986936	ACJ57235.1	ribosomal protein S1	2043970-2045644	Acinetobacter_baumannii_AB307-0294	Alteromonadales
213987378	ACJ57677.1	DNA-directed RNA polymerase, beta' subunit	3456128-3460322	Acinetobacter_baumannii_AB307-0294	Alteromonadales
213987378	ACJ57677.1	DNA-directed RNA polymerase, beta' subunit	3456128-3460322	Acinetobacter_baumannii_AB307-0294	Pasteurellales
213987856	ACJ58155.1	ATP synthase F1, alpha subunit	3581147-3582692	Acinetobacter_baumannii_AB307-0294	Enterobacteriales
213987863	ACJ58162.1	ribosomal protein S3	469061-469814	Acinetobacter_baumannii_AB307-0294	Pasteurellales
213988222	ACJ58521.1	Isovaleryl-CoA dehydrogenase (IVD)	2252179-2253352	Acinetobacter_baumannii_AB307-0294	Alteromonadales
213988798	ACJ59097.1	Ribosomal protein S9/S16 family protein	538656-539043	Acinetobacter_baumannii_AB307-0294	Alteromonadales
213988991	ACJ59290.1	ABC1 family protein	3394245-3395865	Acinetobacter_baumannii_AB307-0294	Alteromonadales
213989017	ACJ59316.1	AMP-binding enzyme family protein	3535274-3536954	Acinetobacter_baumannii_AB307-0294	Pasteurellales
218350314	CAU95997.1	lipoamide dehydrogenase, E3 component is part of three enzyme complexes	128215-129640	Escherichia_coli_55989	Neisseriales
218350368	CAU96051.1	uridylate kinase	191119-191845	Escherichia_coli_55989	Alteromonadales
218351116	CAU96820.1	30S ribosomal subunit protein S1	1023419-1025093	Escherichia_coli_55989	Alteromonadales
218353272	CAU99226.1	methionine adenosyltransferase 1	3316176-3317331	Escherichia_coli_55989	Alteromonadales
218353740	CAV00031.1	30S ribosomal subunit protein S19	3799885-3800164	Escherichia_coli_55989	Alteromonadales
218354377	CAV01135.1	molecular chaperone and ATPase component of HslUV protease	4502169-4503501	Escherichia_coli_55989	Pasteurellales
218354419	CAV01216.1	protein chain elongation factor EF-Tu (duplicate of tufA)	4565802-4566987	Escherichia_coli_55989	Alteromonadales
218354419	CAV01216.1	protein chain elongation factor EF-Tu (duplicate of tufA)	4565802-4566987	Escherichia_coli_55989	Pasteurellales
22533070	AAM98973.1	ribosomal protein L16	80527-80941	Streptococcus_agalactiae_2603V/R	Bacillales
22533169	AAM99067.1	DNA-directed RNA polymerase, beta subunit	179287-182863	Streptococcus_agalactiae_2603V/R	Thermoanaerobacteriales
22533778	AAM99649.1	translation elongation factor Tu	757603-758800	Streptococcus_agalactiae_2603V/R	Bacillales
22534283	AAN00132.1	cadmium efflux system accessory protein	1268733-1269102	Streptococcus_agalactiae_2603V/R	Bacillales
22534410	AAN00253.1	ribosomal protein L20	1392878-1393238	Streptococcus_agalactiae_2603V/R	Bacillales
225700225	CAW94428.1	elongation factor Tu (EF-Tu)	1510904-1512119	Streptococcus_equi_subsp._equi_4047	Pseudomonadales
225701518	CAW98708.1	elongation factor Tu (EF-Tu)	734512-735727	Streptococcus_equi_subsp._zooepidemicus_H70	Bacillales
225702141	CAW99828.1	putative RNA methyltransferase	1447450-1449082	Streptococcus_equi_subsp._zooepidemicus_H70	Pasteurellales
226840847	ACO83513.1	carbamoyl-phosphate synthase, large subunit	2065419-2068626	Clostridium_botulinum_A2_str._Kyoto	Thermoanaerobacteriales
226841129	ACO83795.1	DNA-directed RNA polymerase, beta subunit	3984261-3987960	Clostridium_botulinum_A2_str._Kyoto	Thermoanaerobacteriales
240266863	ACS50451.1	heat shock protein DnaK	85727-87620	Bartonella_grahamii_as4aup	Alteromonadales
242390191	BAH80650.1	DNA-directed RNA polymerase beta subunit	107850-111492	Streptococcus_dysgalactiae_subsp._equisimilis_GGS_124	Bacillales
242390668	BAH81127.1	elongation factor Tu	594750-595965	Streptococcus_dysgalactiae_subsp._equisimilis_GGS_124	Bacillales
253322589	ACT27191.1	phosphoglycerate mutase, 2,3-bisphosphoglycerate-independent	116866-118411	Escherichia_coli_'BL21-Gold(DE3)pLysS_AG'	Alteromonadales
253322649	ACT27251.1	glycyl-tRNA synthetase, alpha subunit	185399-186311	Escherichia_coli_'BL21-Gold(DE3)pLysS_AG'	Pasteurellales
253322728	ACT27330.1	ABC transporter related	280949-283685	Escherichia_coli_'BL21-Gold(DE3)pLysS_AG'	Rhodospirallales
253322876	ACT27478.1	ribosomal protein S12	439650-440025	Escherichia_coli_'BL21-Gold(DE3)pLysS_AG'	Pasteurellales
253322878	ACT27480.1	translation elongation factor G	440688-442803	Escherichia_coli_'BL21-Gold(DE3)pLysS_AG'	Neisseriales
253322878	ACT27480.1	translation elongation factor G	440688-442803	Escherichia_coli_'BL21-Gold(DE3)pLysS_AG'	Pasteurellales
253322879	ACT27481.1	translation elongation factor Tu	442873-444058	Escherichia_coli_'BL21-Gold(DE3)pLysS_AG'	Bacillales
253322879	ACT27481.1	translation elongation factor Tu	442873-444058	Escherichia_coli_'BL21-Gold(DE3)pLysS_AG'	Alteromonadales
253322879	ACT27481.1	translation elongation factor Tu	442873-444058	Escherichia_coli_'BL21-Gold(DE3)pLysS_AG'	Pasteurellales
253322879	ACT27481.1	translation elongation factor Tu	442873-444058	Escherichia_coli_'BL21-Gold(DE3)pLysS_AG'	Pseudomonadales
253322904	ACT27506.1	ribosomal protein S19	462107-462386	Escherichia_coli_'BL21-Gold(DE3)pLysS_AG'	Alteromonadales
253322922	ACT27524.1	ribosomal protein S13	470162-470519	Escherichia_coli_'BL21-Gold(DE3)pLysS_AG'	Alteromonadales
253322924	ACT27526.1	ribosomal protein S4	470958-471579	Escherichia_coli_'BL21-Gold(DE3)pLysS_AG'	Alteromonadales
253322926	ACT27528.1	ribosomal protein L17	472634-473018	Escherichia_coli_'BL21-Gold(DE3)pLysS_AG'	Alteromonadales
253322953	ACT27555.1	transcriptional regulator, Fis family	501080-501377	Escherichia_coli_'BL21-Gold(DE3)pLysS_AG'	Alteromonadales
253323032	ACT27634.1	ATP-dependent metalloprotease FtsH	578360-580304	Escherichia_coli_'BL21-Gold(DE3)pLysS_AG'	Alteromonadales
253323137	ACT27739.1	methyl-accepting chemotaxis sensory transducer with Pas/Pac sensor	688645-690166	Escherichia_coli_'BL21-Gold(DE3)pLysS_AG'	Xanthomonadales
253323141	ACT27743.1	RNA polymerase, sigma 70 subunit, RpoD	692833-694675	Escherichia_coli_'BL21-Gold(DE3)pLysS_AG'	Neisseriales
253323141	ACT27743.1	RNA polymerase, sigma 70 subunit, RpoD	692833-694675	Escherichia_coli_'BL21-Gold(DE3)pLysS_AG'	Alteromonadales
253323312	ACT27914.1	hypothetical protein	872761-873860	Escherichia_coli_'BL21-Gold(DE3)pLysS_AG'	Xanthomonadales
253323395	ACT27997.1	L-serine dehydratase 1	976370-977738	Escherichia_coli_'BL21-Gold(DE3)pLysS_AG'	Pasteurellales
253323490	ACT28092.1	recA protein	1076168-1077230	Escherichia_coli_'BL21-Gold(DE3)pLysS_AG'	Alteromonadales
253323576	ACT28178.1	GTP-binding protein LepA	1166179-1167979	Escherichia_coli_'BL21-Gold(DE3)pLysS_AG'	Alteromonadales
253323591	ACT28193.1	phosphoribosylformylglycinamidine synthase	1178537-1182425	Escherichia_coli_'BL21-Gold(DE3)pLysS_AG'	Alteromonadales
253323683	ACT28285.1	transporter, hydrophobe/amphiphile efflux-1 (HAE1) family	1284706-1287820	Escherichia_coli_'BL21-Gold(DE3)pLysS_AG'	Xanthomonadales
253323886	ACT28488.1	ribonucleotide reductase	1501020-1502151	Escherichia_coli_'BL21-Gold(DE3)pLysS_AG'	Neisseriales
253324211	ACT28813.1	arginyl-tRNA synthetase	1874765-1876499	Escherichia_coli_'BL21-Gold(DE3)pLysS_AG'	Pasteurellales
253324310	ACT28912.1	glyceraldehyde-3-phosphate dehydrogenase, type I	1974335-1975331	Escherichia_coli_'BL21-Gold(DE3)pLysS_AG'	Neisseriales
253325131	ACT29733.1	translation initiation factor IF-1	2850885-2851104	Escherichia_coli_'BL21-Gold(DE3)pLysS_AG'	Alteromonadales
253325348	ACT29950.1	2-oxoglutarate dehydrogenase, E1 subunit	3062307-3065109	Escherichia_coli_'BL21-Gold(DE3)pLysS_AG'	Alteromonadales
253325378	ACT29980.1	Ornithine decarboxylase	3102050-3104249	Escherichia_coli_'BL21-Gold(DE3)pLysS_AG'	Alteromonadales
253325400	ACT30002.1	PhoH family protein	3129092-3130172	Escherichia_coli_'BL21-Gold(DE3)pLysS_AG'	Xanthomonadales

253325614	ACT30216.1	ATP-dependent protease La	3352107-3354462	Escherichia_coli_'BL21-Gold(DE3)pLysS_AG'	Alteromonadales
253325617	ACT30219.1	ATP-dependent Clp protease, ATP-binding subunit ClpX	3355999-3357274	Escherichia_coli_'BL21-Gold(DE3)pLysS_AG'	Alteromonadales
253325625	ACT30227.1	cytochrome o ubiquinol oxidase, subunit I	3364060-3366052	Escherichia_coli_'BL21-Gold(DE3)pLysS_AG'	Xanthomonadales
253325848	ACT30450.1	ribosomal protein S2	3588941-3589667	Escherichia_coli_'BL21-Gold(DE3)pLysS_AG'	Alteromonadales
253325978	ACT30580.1	carbamoyl-phosphate synthase, large subunit	3745611-3748833	Escherichia_coli_'BL21-Gold(DE3)pLysS_AG'	Neisseriales
253325999	ACT30601.1	chaperone protein DnaK	3769644-3771561	Escherichia_coli_'BL21-Gold(DE3)pLysS_AG'	Alteromonadales
253326023	ACT30625.1	ABC transporter related	3794798-3796466	Escherichia_coli_'BL21-Gold(DE3)pLysS_AG'	Xanthomonadales
253326163	ACT30765.1	valyl-tRNA synthetase	3947237-3950093	Escherichia_coli_'BL21-Gold(DE3)pLysS_AG'	Alteromonadales
253326163	ACT30765.1	valyl-tRNA synthetase	3947237-3950093	Escherichia_coli_'BL21-Gold(DE3)pLysS_AG'	Pasteurellales
253326163	ACT30765.1	valyl-tRNA synthetase	3947237-3950093	Escherichia_coli_'BL21-Gold(DE3)pLysS_AG'	Pseudomonadales
253326217	ACT30819.1	ribosomal protein S18	4009886-4010114	Escherichia_coli_'BL21-Gold(DE3)pLysS_AG'	Alteromonadales
253326239	ACT30841.1	RNA methyltransferase, TrmH family, group 3	4025946-4026678	Escherichia_coli_'BL21-Gold(DE3)pLysS_AG'	Neisseriales
253326273	ACT30875.1	chaperonin GroEL	4062715-4064362	Escherichia_coli_'BL21-Gold(DE3)pLysS_AG'	Neisseriales
253326383	ACT30985.1	ABC transporter related	4187201-4188317	Escherichia_coli_'BL21-Gold(DE3)pLysS_AG'	Xanthomonadales
253326401	ACT31003.1	methionine synthase	4207590-4211274	Escherichia_coli_'BL21-Gold(DE3)pLysS_AG'	Xanthomonadales
253326427	ACT31029.1	DNA-directed RNA polymerase, beta' subunit	4244848-4249072	Escherichia_coli_'BL21-Gold(DE3)pLysS_AG'	Xanthomonadales
253326427	ACT31029.1	DNA-directed RNA polymerase, beta' subunit	4244848-4249072	Escherichia_coli_'BL21-Gold(DE3)pLysS_AG'	Neisseriales
253326427	ACT31029.1	DNA-directed RNA polymerase, beta' subunit	4244848-4249072	Escherichia_coli_'BL21-Gold(DE3)pLysS_AG'	Pseudomonadales
253326428	ACT31030.1	DNA-directed RNA polymerase, beta subunit	4249148-4253177	Escherichia_coli_'BL21-Gold(DE3)pLysS_AG'	Alteromonadales
253326428	ACT31030.1	DNA-directed RNA polymerase, beta subunit	4249148-4253177	Escherichia_coli_'BL21-Gold(DE3)pLysS_AG'	Pasteurellales
253326435	ACT31037.1	translation elongation factor Tu	4257293-4258478	Escherichia_coli_'BL21-Gold(DE3)pLysS_AG'	Pasteurellales
253326562	ACT31164.1	UbiD family decarboxylase	4410417-4411911	Escherichia_coli_'BL21-Gold(DE3)pLysS_AG'	Neisseriales
253326646	ACT31248.1	dihydroxy-acid dehydratase	4498239-4500090	Escherichia_coli_'BL21-Gold(DE3)pLysS_AG'	Alteromonadales
253326677	ACT31279.1	ATP synthase F1, beta subunit	4537633-4539016	Escherichia_coli_'BL21-Gold(DE3)pLysS_AG'	Neisseriales
253326707	ACT31309.1	ribosomal protein L34	4570537-4570678	Escherichia_coli_'BL21-Gold(DE3)pLysS_AG'	Pasteurellales
256632242	BAH98217.1	SSU ribosomal protein S1	59165-60884	Acetobacter_pasteurianus_IFO_3283-01	Enterobacteriales
256632485	BAH98460.1	transcription termination factor Rho	320127-321426	Acetobacter_pasteurianus_IFO_3283-01	Desulfuromonadales
256632813	BAH98788.1	RNA helicase	665113-666451	Acetobacter_pasteurianus_IFO_3283-01	Alteromonadales
256632843	BAH98818.1	2,3,4,5-tetrahydropyridine-2-carboxylate N-succinyltransferase	692949-693798	Acetobacter_pasteurianus_IFO_3283-01	Pseudomonadales
256633095	BAH99070.1	LSU ribosomal protein L11P	962229-962664	Acetobacter_pasteurianus_IFO_3283-01	Xanthomonadales
256633099	BAH99074.1	DNA-directed RNA polymerase beta subunit	964835-969029	Acetobacter_pasteurianus_IFO_3283-01	Xanthomonadales
256633099	BAH99074.1	DNA-directed RNA polymerase beta subunit	964835-969029	Acetobacter_pasteurianus_IFO_3283-01	Alteromonadales
256633099	BAH99074.1	DNA-directed RNA polymerase beta subunit	964835-969029	Acetobacter_pasteurianus_IFO_3283-01	Enterobacteriales
256633100	BAH99075.1	DNA-directed RNA polymerase beta' subunit	969122-973298	Acetobacter_pasteurianus_IFO_3283-01	Alteromonadales
256633103	BAH99078.1	translation elongation factor Tu (EF-TU)	974590-975781	Acetobacter_pasteurianus_IFO_3283-01	Desulfuromonadales
256633103	BAH99078.1	translation elongation factor Tu (EF-TU)	974590-975781	Acetobacter_pasteurianus_IFO_3283-01	Enterobacteriales
256633138	BAH99113.1	multidrug efflux pump acriflavin resistance protein AcrB/AcrD/AcrF	998296-1001488	Acetobacter_pasteurianus_IFO_3283-01	Desulfuromonadales
256633165	BAH99140.1	DNA-directed RNA polymerase sigma factor RpoD	1036689-1038708	Acetobacter_pasteurianus_IFO_3283-01	Neisseriales
256633475	BAH99450.1	multidrug transporter ATP-binding protein	1370873-1373609	Acetobacter_pasteurianus_IFO_3283-01	Enterobacteriales
256634611	BAI00587.1	dihydroxy-acid dehydratase	2688970-2690839	Acetobacter_pasteurianus_IFO_3283-01	Xanthomonadales
256635299	BAI01268.1	SSU ribosomal protein S1	59165-60884	Acetobacter_pasteurianus_IFO_3283-03	Enterobacteriales
256635542	BAI01511.1	transcription termination factor Rho	320121-321420	Acetobacter_pasteurianus_IFO_3283-03	Desulfuromonadales
256635870	BAI01839.1	RNA helicase	665107-666445	Acetobacter_pasteurianus_IFO_3283-03	Alteromonadales
256635900	BAI01869.1	2,3,4,5-tetrahydropyridine-2-carboxylate N-succinyltransferase	692943-693792	Acetobacter_pasteurianus_IFO_3283-03	Pseudomonadales
256636152	BAI02121.1	LSU ribosomal protein L11P	962223-962658	Acetobacter_pasteurianus_IFO_3283-03	Xanthomonadales
256636156	BAI02125.1	DNA-directed RNA polymerase beta subunit	964829-969023	Acetobacter_pasteurianus_IFO_3283-03	Xanthomonadales
256636156	BAI02125.1	DNA-directed RNA polymerase beta subunit	964829-969023	Acetobacter_pasteurianus_IFO_3283-03	Alteromonadales
256636156	BAI02125.1	DNA-directed RNA polymerase beta subunit	964829-969023	Acetobacter_pasteurianus_IFO_3283-03	Enterobacteriales
256636157	BAI02126.1	DNA-directed RNA polymerase beta' subunit	969116-973292	Acetobacter_pasteurianus_IFO_3283-03	Alteromonadales
256636160	BAI02129.1	translation elongation factor Tu (EF-TU)	974584-975775	Acetobacter_pasteurianus_IFO_3283-03	Desulfuromonadales
256636160	BAI02129.1	translation elongation factor Tu (EF-TU)	974584-975775	Acetobacter_pasteurianus_IFO_3283-03	Enterobacteriales
256636195	BAI02164.1	multidrug efflux pump acriflavin resistance protein AcrB/AcrD/AcrF	998290-1001482	Acetobacter_pasteurianus_IFO_3283-03	Desulfuromonadales
256636222	BAI02191.1	DNA-directed RNA polymerase sigma factor RpoD	1036683-1038702	Acetobacter_pasteurianus_IFO_3283-03	Neisseriales
256636534	BAI02503.1	multidrug transporter ATP-binding protein	1371884-1374620	Acetobacter_pasteurianus_IFO_3283-03	Enterobacteriales
256637667	BAI03636.1	dihydroxy-acid dehydratase	2688762-2690631	Acetobacter_pasteurianus_IFO_3283-03	Xanthomonadales
256638354	BAI04316.1	SSU ribosomal protein S1	59165-60884	Acetobacter_pasteurianus_IFO_3283-07	Enterobacteriales
256638597	BAI04559.1	transcription termination factor Rho	320121-321420	Acetobacter_pasteurianus_IFO_3283-07	Desulfuromonadales
256638925	BAI04887.1	RNA helicase	665107-666445	Acetobacter_pasteurianus_IFO_3283-07	Alteromonadales
256638955	BAI04917.1	2,3,4,5-tetrahydropyridine-2-carboxylate N-succinyltransferase	692943-693792	Acetobacter_pasteurianus_IFO_3283-07	Pseudomonadales
256639207	BAI05169.1	LSU ribosomal protein L11P	962223-962658	Acetobacter_pasteurianus_IFO_3283-07	Xanthomonadales
256639211	BAI05173.1	DNA-directed RNA polymerase beta subunit	964829-969023	Acetobacter_pasteurianus_IFO_3283-07	Xanthomonadales
256639211	BAI05173.1	DNA-directed RNA polymerase beta subunit	964829-969023	Acetobacter_pasteurianus_IFO_3283-07	Alteromonadales
256639211	BAI05173.1	DNA-directed RNA polymerase beta subunit	964829-969023	Acetobacter_pasteurianus_IFO_3283-07	Enterobacteriales
256639212	BAI05174.1	DNA-directed RNA polymerase beta' subunit	969116-973292	Acetobacter_pasteurianus_IFO_3283-07	Alteromonadales
256639215	BAI05177.1	translation elongation factor Tu (EF-TU)	974584-975775	Acetobacter_pasteurianus_IFO_3283-07	Desulfuromonadales
256639215	BAI05177.1	translation elongation factor Tu (EF-TU)	974584-975775	Acetobacter_pasteurianus_IFO_3283-07	Enterobacteriales
256639250	BAI05212.1	multidrug efflux pump acriflavin resistance protein AcrB/AcrD/AcrF	998290-1001482	Acetobacter_pasteurianus_IFO_3283-07	Desulfuromonadales
256639277	BAI05239.1	DNA-directed RNA polymerase sigma factor RpoD	1036683-1038702	Acetobacter_pasteurianus_IFO_3283-07	Neisseriales
256639587	BAI05549.1	multidrug transporter ATP-binding protein	1370867-1373603	Acetobacter_pasteurianus_IFO_3283-07	Enterobacteriales
256640721	BAI06683.1	dihydroxy-acid dehydratase	2687519-2689388	Acetobacter_pasteurianus_IFO_3283-07	Xanthomonadales
256641408	BAI07363.1	SSU ribosomal protein S1	59165-60884	Acetobacter_pasteurianus_IFO_3283-22	Enterobacteriales
256641651	BAI07606.1	transcription termination factor Rho	320121-321420	Acetobacter_pasteurianus_IFO_3283-22	Desulfuromonadales
256641979	BAI07934.1	RNA helicase	665107-666445	Acetobacter_pasteurianus_IFO_3283-22	Alteromonadales
256642009	BAI07964.1	2,3,4,5-tetrahydropyridine-2-carboxylate N-succinyltransferase	692943-693792	Acetobacter_pasteurianus_IFO_3283-22	Pseudomonadales
256642261	BAI08216.1	LSU ribosomal protein L11P	962223-962658	Acetobacter_pasteurianus_IFO_3283-22	Xanthomonadales
256642265	BAI08220.1	DNA-directed RNA polymerase beta subunit	964829-969023	Acetobacter_pasteurianus_IFO_3283-22	Xanthomonadales
256642265	BAI08220.1	DNA-directed RNA polymerase beta subunit	964829-969023	Acetobacter_pasteurianus_IFO_3283-22	Alteromonadales
256642265	BAI08220.1	DNA-directed RNA polymerase beta subunit	964829-969023	Acetobacter_pasteurianus_IFO_3283-22	Enterobacteriales
256642266	BAI08221.1	DNA-directed RNA polymerase beta' subunit	969116-973292	Acetobacter_pasteurianus_IFO_3283-22	Alteromonadales
256642269	BAI08224.1	translation elongation factor Tu (EF-TU)	974584-975775	Acetobacter_pasteurianus_IFO_3283-22	Desulfuromonadales
256642269	BAI08224.1	translation elongation factor Tu (EF-TU)	974584-975775	Acetobacter_pasteurianus_IFO_3283-22	Enterobacteriales
256642304	BAI08259.1	multidrug efflux pump acriflavin resistance protein AcrB/AcrD/AcrF	998290-1001482	Acetobacter_pasteurianus_IFO_3283-22	Desulfuromonadales
256642331	BAI08286.1	DNA-directed RNA polymerase sigma factor RpoD	1036683-1038702	Acetobacter_pasteurianus_IFO_3283-22	Neisseriales
256642643	BAI08598.1	multidrug transporter ATP-binding protein	1371884-1374620	Acetobacter_pasteurianus_IFO_3283-22	Enterobacteriales
256643776	BAI09731.1	dihydroxy-acid dehydratase	2688742-2690611	Acetobacter_pasteurianus_IFO_3283-22	Xanthomonadales
256650571	BAI16505.1	SSU ribosomal protein S1	59165-60884	Acetobacter_pasteurianus_IFO_3283-01-42C	Enterobacteriales
256651142	BAI17076.1	RNA helicase	665113-666451	Acetobacter_pasteurianus_IFO_3283-01-42C	Alteromonadales
256651172	BAI17106.1	2,3,4,5-tetrahydropyridine-2-carboxylate N-succinyltransferase	692949-693798	Acetobacter_pasteurianus_IFO_3283-01-42C	Pseudomonadales
256651428	BAI17362.1	DNA-directed RNA polymerase beta subunit	964835-969029	Acetobacter_pasteurianus_IFO_3283-01-42C	Alteromonadales
256651428	BAI17362.1	DNA-directed RNA polymerase beta subunit	964835-969029	Acetobacter_pasteurianus_IFO_3283-01-42C	Enterobacteriales
256651429	BAI17363.1	DNA-directed RNA polymerase beta' subunit	969122-973298	Acetobacter_pasteurianus_IFO_3283-01-42C	Alteromonadales
256651432	BAI17366.1	translation elongation factor Tu (EF-TU)	974590-975781	Acetobacter_pasteurianus_IFO_3283-01-42C	Enterobacteriales
256651494	BAI17428.1	DNA-directed RNA polymerase sigma factor RpoD	1036689-1038708	Acetobacter_pasteurianus_IFO_3283-01-42C	Neisseriales
256651804	BAI17738.1	multidrug transporter ATP-binding protein	1370873-1373609	Acetobacter_pasteurianus_IFO_3283-01-42C	Enterobacteriales
256653562	BAI19489.1	SSU ribosomal protein S1	59165-60884	Acetobacter_pasteurianus_IFO_3283-12	Enterobacteriales
256653805	BAI19732.1	transcription termination factor Rho	320121-321420	Acetobacter_pasteurianus_IFO_3283-12	Desulfuromonadales
256654133	BAI20060.1	RNA helicase	665107-666445	Acetobacter_pasteurianus_IFO_3283-12	Alteromonadales
256654163	BAI20090.1	2,3,4,5-tetrahydropyridine-2-carboxylate N-succinyltransferase	692943-693792	Acetobacter_pasteurianus_IFO_3283-12	Pseudomonadales
256654415	BAI20342.1	LSU ribosomal protein L11P	962223-962658	Acetobacter_pasteurianus_IFO_3283-12	Xanthomonadales
256654419	BAI20346.1	DNA-directed RNA polymerase beta subunit	964829-969023	Acetobacter_pasteurianus_IFO_3283-12	Xanthomonadales
256654419	BAI20346.1	DNA-directed RNA polymerase beta subunit	964829-969023	Acetobacter_pasteurianus_IFO_3283-12	Alteromonadales
256654419	BAI20346.1	DNA-directed RNA polymerase beta subunit	964829-969023	Acetobacter_pasteurianus_IFO_3283-12	Enterobacteriales
256654420	BAI20347.1	DNA-directed RNA polymerase beta' subunit	969116-973292	Acetobacter_pasteurianus_IFO_3283-12	Alteromonadales
256654423	BAI20350.1	translation elongation factor Tu (EF-TU)	974584-975775	Acetobacter_pasteurianus_IFO_3283-12	Desulfuromonadales
256654423	BAI20350.1	translation elongation factor Tu (EF-TU)	974584-975775	Acetobacter_pasteurianus_IFO_3283-12	Enterobacteriales
256654458	BAI20385.1	multidrug efflux pump acriflavin resistance protein AcrB/AcrD/AcrF	998290-1001482	Acetobacter_pasteurianus_IFO_3283-12	Desulfuromonadales
256654485	BAI20412.1	DNA-directed RNA polymerase sigma factor RpoD	1036683-1038702	Acetobacter_pasteurianus_IFO_3283-12	Neisseriales

256654795	BAI20722.1	multidrug transporter ATP-binding protein	1370867-1373603	Acetobacter_pasteurianus_IFO_3283-12	Enterobacteriales
256655928	BAI21855.1	dihydroxy-acid dehydratase	2686099-2687968	Acetobacter_pasteurianus_IFO_3283-12	Xanthomonadales
268582261	EEZ46937.1	ATP synthase F1, subunit beta	2205682-2207080	Neisseria_gonorrhoeae_MS11	Pseudomonadales
268582596	EEZ47272.1	queuine tRNA-ribosyltransferase	295440-296556	Neisseria_gonorrhoeae_MS11	Xanthomonadales
268583317	EEZ47993.1	type III restriction enzyme	627716-630491	Neisseria_gonorrhoeae_MS11	Pasteurellales
268583353	EEZ48029.1	MerR family transcriptional regulator	588798-589206	Neisseria_gonorrhoeae_MS11	Pasteurellales
268583355	EEZ48031.1	S-formylglutathione hydrolase	586702-587530	Neisseria_gonorrhoeae_MS11	Pasteurellales
268583604	EEZ48280.1	biotin biosynthesis protein BioC	1516443-1517226	Neisseria_gonorrhoeae_MS11	Pasteurellales
284920761	CBG33824.1	cytidylate kinase	1075711-1076395	Escherichia_coli_042	Alteromonadales
284921602	CBG34674.1	component of SufB-SufC-SufD cysteine desulfurase activator complex	1929847-1931119	Escherichia_coli_042	Alteromonadales
284922820	CBG35908.1	putative aspartate/ornithine carbamoyltransferase	3284228-3285419	Escherichia_coli_042	Pseudomonadales
284922873	CBG35962.1	putative ATP/GTP-binding protein	3350740-3351454	Escherichia_coli_042	Alteromonadales
284923083	CBG36176.1	conserved hypothetical protein	3572277-3572898	Escherichia_coli_042	Neisseriales
284923309	CBG36403.1	30S ribosomal subunit protein S5	3806093-3806597	Escherichia_coli_042	Alteromonadales
284923805	CBG36903.1	phosphate ABC transporter, permease protein	4343896-4344787	Escherichia_coli_042	Pseudomonadales
284923806	CBG36904.1	phosphate ABC transporter, permease protein	4344786-4345746	Escherichia_coli_042	Pseudomonadales
284923807	CBG36905.1	phosphate ABC transporter, substrate-binding protein	4345831-4346872	Escherichia_coli_042	Pseudomonadales
284923808	CBG36906.1	putative fimbrial adhesin	4347119-4348190	Escherichia_coli_042	Pseudomonadales
284923809	CBG36907.1	fimbrial outer membrane usher protein	4348200-4350729	Escherichia_coli_042	Pseudomonadales
284923810	CBG36908.1	fimbrial chaperone protein	4350756-4351488	Escherichia_coli_042	Pseudomonadales
284923814	CBG36912.1	chloramphenicol acetyltransferase	4353194-4353854	Escherichia_coli_042	Pseudomonadales
284923815	CBG36913.1	putative plasmid-related protein	4354054-4354432	Escherichia_coli_042	Pseudomonadales
284923818	CBG36916.1	transposon Tn21 modulator protein	4358153-4358504	Escherichia_coli_042	Pseudomonadales
284923819	CBG36917.1	integrase	4358706-4359720	Escherichia_coli_042	Pseudomonadales
284923821	CBG36919.1	putative ethidium bromide resistance protein	4360823-4361171	Escherichia_coli_042	Pseudomonadales
284923822	CBG36920.1	dihydropteroate synthase type-1	4361164-4362004	Escherichia_coli_042	Pseudomonadales
284923825	CBG36923.1	transposase	4363579-4365103	Escherichia_coli_042	Pseudomonadales
284923826	CBG36924.1	transposition protein	4365204-4366065	Escherichia_coli_042	Pseudomonadales
284923827	CBG36925.1	putative transposase	4366067-4367783	Escherichia_coli_042	Pseudomonadales
284924059	CBG37158.1	N-acetyl-gamma-glutamyl-phosphate reductase	4629644-4630649	Escherichia_coli_042	Alteromonadales
284924059	CBG37158.1	N-acetyl-gamma-glutamyl-phosphate reductase	4629644-4630649	Escherichia_coli_042	Pasteurellales
284924067	CBG37166.1	vitamin B12 TonB-dependent receptor	4638240-4640127	Escherichia_coli_042	Alteromonadales
291599878	ADE19374.1	oligopeptide ABC transporter, ATP-binding protein OppF	717360-719832	Mycoplasma_crocodyli_MP145	Clostridiales
298355599	CBI66471.1	conserved hypothetical protein	901029-901932	Helicobacter_pylori_B8	Enterobacteriales
30253620	AAP24162.1	translation elongation factor Tu	119375-120563	Bacillus_anthracis_str._Ames	Lactobacillales
30253620	AAP24162.1	translation elongation factor Tu	119375-120563	Bacillus_anthracis_str._Ames	Pasteurellales
30253629	AAP24171.1	50S ribosomal protein L16	125097-125532	Bacillus_anthracis_str._Ames	Lactobacillales
302574963	ADL42754.1	Glycine hydroxymethyltransferase	1579888-1581142	Caldicellulosiruptor_obsidiansis_OB47	Clostridiales
307551967	ADN44742.1	dihydrolipoamide dehydrogenase	134705-136130	Escherichia_coli_ABU_83972	Neisseriales
307552750	ADN45525.1	30S ribosomal protein	987782-989456	Escherichia_coli_ABU_83972	Alteromonadales
307554753	ADN47528.1	enolase	3104154-3105453	Escherichia_coli_ABU_83972	Bacillales
307554916	ADN47691.1	transketolase 1	3295351-3297343	Escherichia_coli_ABU_83972	Alteromonadales
307556123	ADN48898.1	elongation factor Tu	4576955-4578140	Escherichia_coli_ABU_83972	Alteromonadales
307556123	ADN48898.1	elongation factor Tu	4576955-4578140	Escherichia_coli_ABU_83972	Pasteurellales
307604862	CBI41233.1	RNA polymerase (beta subunit)	122978-126560	Bacillus_amyloliquefaciens_DSM7	Lactobacillales
307604862	CBI41233.1	RNA polymerase (beta subunit)	122978-126560	Bacillus_amyloliquefaciens_DSM7	Thermoanaerobacteriales
307604868	CBI41239.1	elongation factor Tu	133930-135121	Bacillus_amyloliquefaciens_DSM7	Lactobacillales
307604868	CBI41239.1	elongation factor Tu	133930-135121	Bacillus_amyloliquefaciens_DSM7	Pseudomonadales
307604874	CBI41245.1	ribosomal protein L2 (BL2)	138382-139216	Bacillus_amyloliquefaciens_DSM7	Lactobacillales
309750015	ADO79999.1	Elongation factor G (EF-G)	3156-5259	Haemophilus_influenzae_R2866	Pseudomonadales
309750016	ADO80000.1	Elongation factor Tu (EF-Tu)	5323-6508	Haemophilus_influenzae_R2866	Bacillales
309750074	ADO80058.1	DNA-directed RNA polymerase beta chain	60398-64430	Haemophilus_influenzae_R2866	Pseudomonadales
309750128	ADO80112.1	Thymidylate kinase	119008-119668	Haemophilus_influenzae_R2866	Neisseriales
309750129	ADO80113.1	DNA polymerase III, delta subunit	122010-122994	Haemophilus_influenzae_R2866	Neisseriales
309750398	ADO80382.1	Putative transcriptional regulator	404347-404755	Haemophilus_influenzae_R2866	Neisseriales
309750399	ADO80383.1	Formaldehyde dehydrogenase, glutathione-dependent	404877-406014	Haemophilus_influenzae_R2866	Neisseriales
309750400	ADO80384.1	Probable S-formylglutathione hydrolase	406022-406850	Haemophilus_influenzae_R2866	Neisseriales
309750474	ADO80458.1	4.5S-RNP protein, GTP-binding export factor, part of signal recognition particle with 4.5 RNA	492380-493769	Haemophilus_influenzae_R2866	Alteromonadales
309750665	ADO80649.1	GTP-binding membrane protein	683366-685163	Haemophilus_influenzae_R2866	Pseudomonadales
309750696	ADO80680.1	Adhesin Hia	723605-726896	Haemophilus_influenzae_R2866	Neisseriales
309751044	ADO81028.1	Putative biotin synthesis protein	1108305-1109088	Haemophilus_influenzae_R2866	Neisseriales
309751045	ADO81029.1	Conserved hypothetical protein	1109075-1109723	Haemophilus_influenzae_R2866	Neisseriales
309751046	ADO81030.1	Probable 8-amino-7-oxononanoate synthase	1109732-1110875	Haemophilus_influenzae_R2866	Neisseriales
309751154	ADO81138.1	Phosphate acetyltransferase	1222170-1224306	Haemophilus_influenzae_R2866	Pseudomonadales
309751295	ADO81279.1	Type III restriction-modification system methylase (M.HindVIP)	1368659-1370663	Haemophilus_influenzae_R2866	Neisseriales
309751296	ADO81280.1	Type III restriction-modification system restriction enzyme (HindVIP)	1370652-1373439	Haemophilus_influenzae_R2866	Neisseriales
309751405	ADO81389.1	Catalase	1493424-1494951	Haemophilus_influenzae_R2866	Pseudomonadales
309751479	ADO81463.1	ATP-dependent Clp protease ATPase subunit	1579438-1582009	Haemophilus_influenzae_R2866	Pseudomonadales
309751790	ADO81774.1	Probable hydrolase (HAD superfamily)	1909494-1910313	Haemophilus_influenzae_R2866	Neisseriales
309972269	ADO95470.1	Elongation factor G (EF-G)	3157-5260	Haemophilus_influenzae_R2846	Pseudomonadales
309972306	ADO95507.1	GroEL, chaperone Hsp60	37225-38881	Haemophilus_influenzae_R2846	Pseudomonadales
309972387	ADO95588.1	Thymidylate kinase	124188-124848	Haemophilus_influenzae_R2846	Neisseriales
309972388	ADO95589.1	DNA polymerase III, delta subunit	124844-125828	Haemophilus_influenzae_R2846	Neisseriales
309972509	ADO95710.1	23S rRNA m5U1939 methyltransferase	244977-246570	Haemophilus_influenzae_R2846	Lactobacillales
309972608	ADO95809.1	Adhesion and penetration protein precursor	341161-345319	Haemophilus_influenzae_R2846	Neisseriales
309972693	ADO95894.1	Putative transcriptional regulator	453539-453947	Haemophilus_influenzae_R2846	Neisseriales
309972694	ADO95895.1	Formaldehyde dehydrogenase, glutathione-dependent	454068-455205	Haemophilus_influenzae_R2846	Neisseriales
309972695	ADO95896.1	Probable S-formylglutathione hydrolase	455213-456041	Haemophilus_influenzae_R2846	Neisseriales
309972879	ADO96080.1	Hsf protein	677160-678033	Haemophilus_influenzae_R2846	Neisseriales
309973051	ADO96252.1	Threonyl-tRNA synthetase	901630-903562	Haemophilus_influenzae_R2846	Pseudomonadales
309973224	ADO96425.1	Putative biotin synthesis protein	1074464-1075247	Haemophilus_influenzae_R2846	Neisseriales
309973225	ADO96426.1	Conserved hypothetical protein	1075234-1075882	Haemophilus_influenzae_R2846	Neisseriales
309973226	ADO96427.1	Probable 8-amino-7-oxononanoate synthase	1075891-1077034	Haemophilus_influenzae_R2846	Neisseriales
309973469	ADO96670.1	Type III restriction-modification system restriction enzyme (HindVIP)	1342981-1345768	Haemophilus_influenzae_R2846	Neisseriales
309973894	ADO97095.1	Probable hydrolase (HAD superfamily)	1804336-1805155	Haemophilus_influenzae_R2846	Neisseriales
311774797	ADQ04284.1	seryl-tRNA synthetase	816807-818070	Caldicellulosiruptor_owensensis_OL	Bacillales
311777462	ADQ06948.1	ATP-dependent chaperone ClpB	1257385-1259980	Caldicellulosiruptor_hydrothermalis_108	Clostridiales
311778110	ADQ07596.1	threonyl-tRNA synthetase	1919652-1921572	Caldicellulosiruptor_hydrothermalis_108	Clostridiales
311778238	ADQ07724.1	ATP-dependent metalloprotease FtsH	2058654-2060505	Caldicellulosiruptor_hydrothermalis_108	Clostridiales
312180201	ADQ40371.1	ATPase AAA-2 domain protein	914670-917160	Caldicellulosiruptor_kristjanssonii_177R1B	Bacillales
312181137	ADQ41307.1	acetolactate synthase, large subunit, biosynthetic type	1872307-1873915	Caldicellulosiruptor_kristjanssonii_177R1B	Clostridiales
312181210	ADQ41380.1	translation elongation factor Tu	1958750-1959953	Caldicellulosiruptor_kristjanssonii_177R1B	Clostridiales
312950711	ADR25306.1	ribosomal protein L2	306633-307479	Mycoplasma_bovis_PG45_clone_MU_clone_A2	Bacillales
313004889	CBN86315.1	DNA-directed RNA polymerase beta' chain	82468-86644	Neisseria_lactamica_020-06	Pseudomonadales
313005388	CBN86822.1	acyl carrier protein	580130-580376	Neisseria_lactamica_020-06	Enterobacteriales
313005536	CBN86972.1	conserved hypothetical protein	763206-764073	Neisseria_lactamica_020-06	Enterobacteriales
313005615	CBN87052.1	putative integrase	845109-845796	Neisseria_lactamica_020-06	Pasteurellales
313005710	CBN87164.1	hypothetical protein	986257-986797	Neisseria_lactamica_020-06	Bacillales
313005900	CBN87356.1	putative sodium-dependent transporter	1214546-1215911	Neisseria_lactamica_020-06	Pasteurellales
313005901	CBN87357.1	cytidylate kinase	1216156-1216813	Neisseria_lactamica_020-06	Pasteurellales
313005902	CBN87358.1	30S ribosomal protein S1	1216866-1218654	Neisseria_lactamica_020-06	Pasteurellales
313005980	CBN87437.1	ATP-dependent Clp protease ATP-binding subunit	1313622-1314867	Neisseria_lactamica_020-06	Pasteurellales
313006018	CBN87477.1	hypothetical protein	1370035-1370221	Neisseria_lactamica_020-06	Pasteurellales
313006189	CBN87651.1	hypothetical membrane protein	1567187-1567547	Neisseria_lactamica_020-06	Bacillales
313006535	CBN87999.1	conserved hypothetical membrane protein	1931019-1931277	Neisseria_lactamica_020-06	Pasteurellales
313006660	CBN88126.1	putative lipoprotein	2062311-2062482	Neisseria_lactamica_020-06	Alteromonadales
313006660	CBN88126.1	putative lipoprotein	2062311-2062482	Neisseria_lactamica_020-06	Enterobacteriales
315471695	ADU28298.1	DNA gyrase, B subunit	4947-6870	Bacillus_cellulosilyticus_DSM_2522	Clostridiales
315471802	ADU28405.1	translation elongation factor Tu	147783-148974	Bacillus_cellulosilyticus_DSM_2522	Lactobacillales
315471812	ADU28415.1	ribosomal protein L2	158436-159267	Bacillus_cellulosilyticus_DSM_2522	Lactobacillales
315471874	ADU28477.1	transcriptional regulator, ArsR family	209568-209937	Bacillus_cellulosilyticus_DSM_2522	Lactobacillales
315472053	ADU28656.1	chaperonin GroEL	399186-400824	Bacillus_cellulosilyticus_DSM_2522	Desulfuromonadales
315472053	ADU28656.1	chaperonin GroEL	399186-400824	Bacillus_cellulosilyticus_DSM_2522	Enterobacteriales
315472170	ADU28773.1	alpha amylase catalytic region	542289-543951	Bacillus_cellulosilyticus_DSM_2522	Clostridiales

315474083	ADU30686.1	recA protein	2627981-2629031	Bacillus_cellulosilyticus_DSM_2522	Lactobacillales
315474729	ADU31332.1	mercuric reductase	3281619-3283260	Bacillus_cellulosilyticus_DSM_2522	Lactobacillales
322506220	ADX01674.1	Chaperone protein dnaK	42274-44215	Acinetobacter_baumannii_1656-2	Pasteurellales
322506367	ADX01821.1	atpD	204220-205615	Acinetobacter_baumannii_1656-2	Neisseriales
322506437	ADX01891.1	Streptomycin resistance protein B	280934-281771	Acinetobacter_baumannii_1656-2	Xanthomonadales
322506438	ADX01892.1	StrA	281770-282754	Acinetobacter_baumannii_1656-2	Xanthomonadales
322506503	ADX01957.1	tufA	348699-349890	Acinetobacter_baumannii_1656-2	Alteromonadales
322506511	ADX01965.1	rpoC	358039-362233	Acinetobacter_baumannii_1656-2	Xanthomonadales
322506669	ADX02123.1	rpmG	535389-535545	Acinetobacter_baumannii_1656-2	Pasteurellales
322507035	ADX02489.1	Elongation factor G	942094-944233	Acinetobacter_baumannii_1656-2	Pasteurellales
322507036	ADX02490.1	Protein chain elongation factor EF-Tu	944327-945518	Acinetobacter_baumannii_1656-2	Alteromonadales
322507318	ADX02772.1	Mercuric reductase	1237619-1239683	Acinetobacter_baumannii_1656-2	Enterobacteriales
322507330	ADX02784.1	Transposase for transposon Tn3	1251005-1251530	Acinetobacter_baumannii_1656-2	Pasteurellales
322507665	ADX03119.1	DNA polymerase III, tau and gamma subunits	1594045-1596178	Acinetobacter_baumannii_1656-2	Pasteurellales
322508156	ADX03610.1	Putative GTP-binding protein	2091922-2093014	Acinetobacter_baumannii_1656-2	Pasteurellales
322508580	ADX04034.1	Catalase	2529595-2531116	Acinetobacter_baumannii_1656-2	Pasteurellales
322509142	ADX04596.1	groEL	3112626-3114261	Acinetobacter_baumannii_1656-2	Pasteurellales
323126356	ADX23653.1	DNA-directed RNA polymerase subunit beta'	110134-113776	Streptococcus_dysgalactiae_subsp._equisimilis_ATCC_12394	Bacillales
323126895	ADX24192.1	elongation factor Tu	638929-640126	Streptococcus_dysgalactiae_subsp._equisimilis_ATCC_12394	Bacillales
33147660	AAP95182.1	chaperone protein DnaK	132285-134190	Haemophilus_ducreyi_strain_35000HP	Bacillales
33147660	AAP95182.1	chaperone protein DnaK	132285-134190	Haemophilus_ducreyi_strain_35000HP	Pseudomonadales
33148061	AAP95582.1	elongation factor G	518963-521069	Haemophilus_ducreyi_strain_35000HP	Enterobacteriales
33148061	AAP95582.1	elongation factor G	518963-521069	Haemophilus_ducreyi_strain_35000HP	Thiotrichales
33148062	AAP95583.1	elongation factor Tu	521130-522315	Haemophilus_ducreyi_strain_35000HP	Bacillales
33148121	AAP95642.1	50S ribosomal protein L33	576142-576313	Haemophilus_ducreyi_strain_35000HP	Pseudomonadales
33148142	AAP95663.1	50S ribosomal protein L34	593242-593377	Haemophilus_ducreyi_strain_35000HP	Enterobacteriales
33148224	AAP95744.1	peptide chain release factor 1 (RF-1)	681782-682865	Haemophilus_ducreyi_strain_35000HP	Enterobacteriales
33148259	AAP95779.1	transcription termination factor Rho	716333-717596	Haemophilus_ducreyi_strain_35000HP	Thiotrichales
33148354	AAP95874.1	putative GTP-binding protein	790411-791503	Haemophilus_ducreyi_strain_35000HP	Pseudomonadales
33148401	AAP95921.1	30S ribosomal protein S18	834021-834249	Haemophilus_ducreyi_strain_35000HP	Alteromonadales
33148964	AAP96482.1	transketolase	1435763-1437761	Haemophilus_ducreyi_strain_35000HP	Pseudomonadales
33149052	AAP96569.1	DNA polymerase III subunits gamma and tau	1524361-1526425	Haemophilus_ducreyi_strain_35000HP	Pseudomonadales
33149090	AAP96607.1	RNA polymerase beta' subunit	1572051-1576314	Haemophilus_ducreyi_strain_35000HP	Neisseriales
33149091	AAP96608.1	RNA polymerase beta subunit	1576479-1580508	Haemophilus_ducreyi_strain_35000HP	Pseudomonadales
33149125	AAP96642.1	prolyl-tRNA synthetase	1612593-1614309	Haemophilus_ducreyi_strain_35000HP	Pseudomonadales
33149170	AAP96687.1	insertion element IS1 1/5/6 protein InsB	1645367-1645871	Haemophilus_ducreyi_strain_35000HP	Enterobacteriales
33149171	AAP96688.1	insertion element IS1 1/2/3/5/6 protein InsA	1645789-1646065	Haemophilus_ducreyi_strain_35000HP	Enterobacteriales
33149172	AAP96689.1	hypothetical protein	1645994-1646105	Haemophilus_ducreyi_strain_35000HP	Enterobacteriales
33149177	AAP96694.1	30S ribosomal protein S3	1647150-1647855	Haemophilus_ducreyi_strain_35000HP	Enterobacteriales
333816855	AEG09521.1	translation elongation factor Tu	268580-269765	Shewanella_baltica_BA175	Enterobacteriales
333816867	AEG09533.1	translation elongation factor Tu	285343-286528	Shewanella_baltica_BA175	Enterobacteriales
333816873	AEG09539.1	ribosomal protein S19	289624-289903	Shewanella_baltica_BA175	Enterobacteriales
333816932	AEG09598.1	ribulose-phosphate 3-epimerase	348813-349488	Shewanella_baltica_BA175	Pseudomonadales
333816953	AEG09619.1	arginine/ornithine antiporter	371133-372453	Shewanella_baltica_BA175	Enterobacteriales
333817039	AEG09705.1	porphobilinogen deaminase	479210-480143	Shewanella_baltica_BA175	Enterobacteriales
333817095	AEG09761.1	transcriptional regulator, Fis family	530805-531111	Shewanella_baltica_BA175	Enterobacteriales
333817810	AEG10476.1	ATP-binding cassette protein, ChvD family	1430538-1432206	Shewanella_baltica_BA175	Enterobacteriales
333817846	AEG10512.1	recA protein	1478831-1479899	Shewanella_baltica_BA175	Enterobacteriales
333817851	AEG10517.1	Valyl-tRNA synthetase	1486842-1489719	Shewanella_baltica_BA175	Enterobacteriales
333817851	AEG10517.1	Valyl-tRNA synthetase	1486842-1489719	Shewanella_baltica_BA175	Pseudomonadales
333817963	AEG10629.1	Ribosomal RNA large subunit methyltransferase N	1601338-1602460	Shewanella_baltica_BA175	Pseudomonadales
333817976	AEG10642.1	Phosphoribosylformylglycinamidine synthase	1617875-1621757	Shewanella_baltica_BA175	Enterobacteriales
333818107	AEG10773.1	Prolyl-tRNA synthetase	1763961-1765677	Shewanella_baltica_BA175	Pasteurellales
333818362	AEG11028.1	DNA polymerase III, subunits gamma and tau	2082380-2085758	Shewanella_baltica_BA175	Enterobacteriales
333818467	AEG11133.1	2-oxoglutarate dehydrogenase, E1 subunit	2201153-2203985	Shewanella_baltica_BA175	Enterobacteriales
333818598	AEG11264.1	Nucleoside diphosphate kinase	2358581-2359013	Shewanella_baltica_BA175	Pseudomonadales
333819016	AEG11682.1	Acetaldehyde dehydrogenase (acetylating)	2878016-2880620	Shewanella_baltica_BA175	Clostridiales
333819148	AEG11814.1	putative serine protein kinase, PrkA	3026762-3028697	Shewanella_baltica_BA175	Enterobacteriales
333819310	AEG11976.1	anti-sigma H sporulation factor, LonB	3217466-3219824	Shewanella_baltica_BA175	Enterobacteriales
333819310	AEG11976.1	anti-sigma H sporulation factor, LonB	3217466-3219824	Shewanella_baltica_BA175	Pasteurellales
333819311	AEG11977.1	ATP-dependent Clp protease ATP-binding subunit clpX	3219956-3221237	Shewanella_baltica_BA175	Enterobacteriales
333819456	AEG12122.1	ribosomal protein S2	3401454-3402183	Shewanella_baltica_BA175	Enterobacteriales
333819678	AEG12344.1	Glutamate synthase (ferredoxin)	3678446-3682895	Shewanella_baltica_BA175	Enterobacteriales
333820015	AEG12681.1	S-adenosylmethionine synthase	4094654-4095806	Shewanella_baltica_BA175	Enterobacteriales
333820387	AEG13053.1	Protein translocase subunit secA	4533018-4535754	Shewanella_baltica_BA175	Pseudomonadales
333820418	AEG13084.1	50S ribosomal protein L33	4572172-4572346	Shewanella_baltica_BA175	Pasteurellales
333820758	AEG13424.1	transporter, hydrophobe/amphiphile efflux-1 (HAE1) family	4941201-4944336	Shewanella_baltica_BA175	Xanthomonadales
334861462	AEH11933.1	transporter, hydrophobe/amphiphile efflux-1 (HAE1) family	150195-153330	Shewanella_baltica_OS117	Xanthomonadales
334865482	AEH15953.1	50S ribosomal protein L17	4964009-4964405	Shewanella_baltica_OS117	Pasteurellales
334865504	AEH15975.1	ribosomal protein S19	4974595-4974874	Shewanella_baltica_OS117	Enterobacteriales
334865514	AEH15985.1	DNA-directed RNA polymerase subunit beta'	4982508-4986726	Shewanella_baltica_OS117	Pseudomonadales
334865515	AEH15986.1	DNA-directed RNA polymerase subunit beta	4986810-4990842	Shewanella_baltica_OS117	Enterobacteriales
334865515	AEH15986.1	DNA-directed RNA polymerase subunit beta	4986810-4990842	Shewanella_baltica_OS117	Pseudomonadales
384383826	AFH81487.1	Elongation factor Tu	119375-120563	Bacillus_anthracis_str._H9401	Lactobacillales
384383826	AFH81487.1	Elongation factor Tu	119375-120563	Bacillus_anthracis_str._H9401	Pasteurellales
384383835	AFH81496.1	50S ribosomal protein L16	125097-125532	Bacillus_anthracis_str._H9401	Lactobacillales
407050250	AFS78295.1	phosphate-binding protein PstS	1387755-1388664	Clostridium_acidurici_9a	Thermoanaerobacteriales
407051317	AFS79362.1	ATP-dependent metalloprotease FtsH	2482236-2484048	Clostridium_acidurici_9a	Thermoanaerobacteriales
471209799	AGI06549.1	multidrug efflux transporter	852401-855551	Xanthomonas_citri_subsp._citri_Aw12879	Enterobacteriales
471210381	AGI07131.1	Multidrug efflux transporter	1490767-1493911	Xanthomonas_citri_subsp._citri_Aw12879	Enterobacteriales
471210796	AGI07546.1	ABC-type sugar transport system, ATPase component	1946575-1947664	Xanthomonas_citri_subsp._citri_Aw12879	Enterobacteriales
471212120	AGI08870.1	Cytochrome O ubiquinol oxidase subunit I	3459596-3461597	Xanthomonas_citri_subsp._citri_Aw12879	Enterobacteriales
47500512	AAT29188.1	translation elongation factor Tu	119375-120563	Bacillus_anthracis_str._'Ames_Ancestor'	Lactobacillales
47500512	AAT29188.1	translation elongation factor Tu	119375-120563	Bacillus_anthracis_str._'Ames_Ancestor'	Pasteurellales
49237857	CAF27052.1	ABC transporter, ATP-binding protein	316413-318024	Bartonella_henselae_strain_Houston-1	Pseudomonadales
50082971	AAT29197.2	ribosomal protein L16	125097-125532	Bacillus_anthracis_str._'Ames_Ancestor'	Lactobacillales
509080981	AGN13229.1	elongation factor Tu	361344-362040	Legionella_pneumophila_subsp._pneumophila_str._Thunder	Alteromonadales
509080993	AGN13241.1	GTPases translation elongation factor	377131-378322	Legionella_pneumophila_subsp._pneumophila_str._Thunder	Alteromonadales
52627671	AAU26412.1	elongation factor Tu (EF-Tu)	365434-366625	Legionella_pneumophila_subsp._pneumophila_str._Philadelphia	Alteromonadales
52627683	AAU26424.1	translation elongation factor Tu (EF-Tu)	381716-382907	Legionella_pneumophila_subsp._pneumophila_str._Philadelphia	Alteromonadales
52627900	AAU26641.1	hypothetical protein	586503-586674	Legionella_pneumophila_subsp._pneumophila_str._Philadelphia	Alteromonadales
52628940	AAU27681.1	hypothetical protein	1766722-1766929	Legionella_pneumophila_subsp._pneumophila_str._Philadelphia	Pasteurellales
53753139	CAH14586.1	elongation factor Tu	414390-415581	Legionella_pneumophila_str._Lens	Alteromonadales
53753151	CAH14598.1	elongation factor Tu	430672-431863	Legionella_pneumophila_str._Lens	Alteromonadales
537637365	EEZ48009.2	transcription-repair coupling factor	609065-612770	Neisseria_gonorrhoeae_MS11	Xanthomonadales
537637462	EEZ47583.2	thymidylate kinase	1016387-1016693	Neisseria_gonorrhoeae_MS11	Pasteurellales
537637546	EEZ48529.2	HAD hydrolase, family IIB	1191307-1191652	Neisseria_gonorrhoeae_MS11	Pasteurellales
537637719	EEZ48736.2	NADH-quinone oxidoreductase subunit I	1774342-1774822	Neisseria_gonorrhoeae_MS11	Xanthomonadales
58426290	AAW75327.1	5-methyltetrahydrofolate-homocysteine methyltransferase	2176323-2179077	Xanthomonas_oryzae_pv._oryzae_KACC_10331	Alteromonadales
58427496	AAW76533.1	ATPases involved in chromosome partitioning	3512494-3513352	Xanthomonas_oryzae_pv._oryzae_KACC_10331	Alteromonadales
58428448	AAW77485.1	dihydroxy-acid dehydratase	4506231-4508070	Xanthomonas_oryzae_pv._oryzae_KACC_10331	Enterobacteriales
58428520	AAW77557.1	transcription termination factor Rho	4579790-4581680	Xanthomonas_oryzae_pv._oryzae_KACC_10331	Enterobacteriales
59717925	AAW89330.1	MerR family transcriptional regulator	588392-588800	Neisseria_gonorrhoeae_FA_1090	Pasteurellales
59717946	AAW89351.1	transcription-repair coupling factor	608659-612364	Neisseria_gonorrhoeae_FA_1090	Xanthomonadales
59717962	AAW89367.1	type III restriction-modification system HindVIP enzyme res	627311-630086	Neisseria_gonorrhoeae_FA_1090	Pasteurellales
59717963	AAW89368.1	restriction endonuclease subunit M	630075-632346	Neisseria_gonorrhoeae_FA_1090	Pasteurellales
59718714	AAW90119.1	malonyl-CoA O-methyltransferase	1443639-1444422	Neisseria_gonorrhoeae_FA_1090	Pasteurellales
59718887	AAW90292.1	2,3,4,5-tetrahydropyridine-2,6-carboxylate N-succinyltransferase	1621292-1622114	Neisseria_gonorrhoeae_FA_1090	Alteromonadales
59718959	AAW90364.1	NADH dehydrogenase	1702844-1703324	Neisseria_gonorrhoeae_FA_1090	Xanthomonadales
59719342	AAW90747.1	ATP synthase F0F1 subunit beta	2126518-2127916	Neisseria_gonorrhoeae_FA_1090	Pseudomonadales
66575436	AAY50846.1	RNA polymerase sigma-70 factor	4499597-4501472	Xanthomonas_campestris_pv._campestris_str._8004	Alteromonadales
68056872	AAX87125.1	transposon Tn3 transposase	124954-127960	Haemophilus_influenzae_86-028NP	Pseudomonadales
68056873	AAX87126.1	hypothetical protein	126924-127041	Haemophilus_influenzae_86-028NP	Pseudomonadales

68056988	AAX87241.1	conserved hypothetical protein	260664-261492	Haemophilus_influenzae_86-028NP	Neisseriales
68057054	AAX87307.1	adhesion and penetration protein Hap	338009-342188	Haemophilus_influenzae_86-028NP	Neisseriales
68057254	AAX87507.1	DNA polymerase III, delta' subunit	543070-544054	Haemophilus_influenzae_86-028NP	Neisseriales
68057255	AAX87508.1	hypothetical protein	544050-544179	Haemophilus_influenzae_86-028NP	Neisseriales
68057256	AAX87509.1	autotransported protein Lav	544253-546377	Haemophilus_influenzae_86-028NP	Neisseriales
68057257	AAX87510.1	thymidylate kinase	547282-547942	Haemophilus_influenzae_86-028NP	Neisseriales
68057408	AAX87661.1	elongation factor G	707406-709509	Haemophilus_influenzae_86-028NP	Pseudomonadales
68057409	AAX87662.1	elongation factor Tu	709573-710758	Haemophilus_influenzae_86-028NP	Bacillales
68057425	AAX87678.1	putative NADPH-quinone reductase, modulator of drug activity B	723725-724304	Haemophilus_influenzae_86-028NP	Neisseriales
68057529	AAX87782.1	prolyl-tRNA synthetase	850698-852417	Haemophilus_influenzae_86-028NP	Pseudomonadales
68057595	AAX87848.1	30S ribosomal protein S11	916420-916810	Haemophilus_influenzae_86-028NP	Alteromonadales
68057815	AAX88068.1	conserved hypothetical protein	1157521-1158118	Haemophilus_influenzae_86-028NP	Neisseriales
68057816	AAX88069.1	putative type III restriction-modification system HindVIP enzyme res	1158123-1160289	Haemophilus_influenzae_86-028NP	Neisseriales
68057919	AAX88172.1	S-adenosylmethionine synthetase	1275125-1276280	Haemophilus_influenzae_86-028NP	Alteromonadales
68057966	AAX88219.1	predicted membrane protein	1324783-1325800	Haemophilus_influenzae_86-028NP	Neisseriales
68058197	AAX88450.1	conserved hypothetical transposase-like protein	1536127-1536499	Haemophilus_influenzae_86-028NP	Neisseriales
68058317	AAX88570.1	threonyl-tRNA synthetase	1642689-1644621	Haemophilus_influenzae_86-028NP	Alteromonadales
76562167	ABA44751.1	ribosomal protein L16	80237-80651	Streptococcus_agalactiae_A909	Bacillales
76562879	ABA45463.1	translation elongation factor Tu	834255-835452	Streptococcus_agalactiae_A909	Bacillales
76562971	ABA45555.1	IS10R, transposase	2042761-2043970	Streptococcus_agalactiae_A909	Enterobacteriales
76563191	ABA45775.1	ribosomal protein L20	1399556-1399916	Streptococcus_agalactiae_A909	Bacillales
76563237	ABA45821.1	ribosomal protein L2	77997-78831	Streptococcus_agalactiae_A909	Bacillales
78037898	CAJ25643.1	RNA polymerase sigma-70 factor	4490896-4492774	Xanthomonas_campestris_pv._vesicatoria	Alteromonadales
83283561	ABC01493.1	translation elongation factor Tu	183879-185067	Mycoplasma_capricolum_subsp._capricolum_ATCC_27343	Bacillales
83283748	ABC01680.1	glycyl-tRNA synthetase	603526-604897	Mycoplasma_capricolum_subsp._capricolum_ATCC_27343	Clostridiales
859212471	AAW89329.2	S-formylglutathione hydrolase	586296-587124	Neisseria_gonorrhoeae_FA_1090	Pasteurellales
859212545	AKO63675.1	hypothetical protein	954704-954947	Neisseria_gonorrhoeae_FA_1090	Pasteurellales
859212586	AKO63697.1	transposase	1148118-1148292	Neisseria_gonorrhoeae_FA_1090	Pasteurellales

< end of Supplementary Table 31 >

Section 8. Numerical data supporting main text Figures 4 and 5.

Supplementary Table 32. Numbers of lateral genes, LGT network edges and nodes inferred for the ECS dataset at *k* = 20, 30 or 40.

k size	Number of lateral genes	Number of edges	Number of nodes in maximum clique
20	58076	25	6
30	64071	22	6
40	70849	22	6

Supplementary Table 33. Number of inferred LGT genes in the BA dataset at $20 \leq k \leq 40$, analysed at the level of phylum or class.

k size	Number of lateral genes detected at phylum level	Number of lateral genes detected at class level
20	12880	28628
25	686	3043
30	167	1076
35	69	576
40	0	310

Supplementary Table 34. Number of inferred lateral genes in the BAC dataset at k = 25, 30, 35 or 40.

k size	Number of lateral genes
25	694
30	241
35	169
40	115

Section 9. Genomes, NCBI accession numbers and group information for the ECS, EB, BA and BAC datasets.

ECS dataset:

1	NC_007779	Escherichia coli str. K-12 substr. W3110
2	NC_000913	Escherichia coli str. K-12 substr. MG1655
3	NC_002655	Escherichia coli O157:H7 str. EDL933
4	NC_002695	Escherichia coli O157:H7 str. Sakai
5	NC_004337	Shigella flexneri 2a str. 301
6	NC_004431	Escherichia coli CFT073
7	NC_004741	Shigella flexneri 2a str. 2457T
8	NC_007384	Shigella sonnei Ss046
9	NC_007606	Shigella dysenteriae Sd197
10	NC_007613	Shigella boydii Sb227
11	NC_007946	Escherichia coli UTI89
12	NC_008253	Escherichia coli 536
13	NC_008258	Shigella flexneri 5 str. 8401
14	NC_008563	Escherichia coli APEC O1
15	NC_009800	Escherichia coli HS
16	NC_009801	Escherichia coli E24377A
17	NC_010468	Escherichia coli ATCC 8739
18	NC_010498	Escherichia coli SMS-3-5
19	NC_010658	Shigella boydii CDC 3083-94
20	NC_011415	Escherichia coli SE11 DNA
21	NC_011601	Escherichia coli 0127:H6 E2348/6 strain E2348/69.
22	NC_011741	Escherichia coli IAI1
23	NC_011742	Escherichia coli S88
24	NC_011745	Escherichia coli ED1a
25	NC_011748	Escherichia coli 55989
26	NC_011750	Escherichia coli IAI39
27	NC_011751	Escherichia coli UMN026

Nodes grouped by MLST; ID numbers as above:

Node A	1 2 15 17
Node B1	16 20 22 25
Node B2	6 11 12 14 21 23 24
Node D	18 26 27
Node E	3 4
Node S	5 7 8 9 10 13 19

EB dataset:

1	AM286415	Yersinia_enterocolitica_subsp._enterocolitica_8081
2	CP002246	Yersinia_enterocolitica_subsp._palearctica_105.5R(r)
3	CP002956	Yersinia_pestis_A1122
4	CP000901	Yersinia_pestis_Angola
5	CP000308	Yersinia_pestis_Antiqua
6	CP001608	Yersinia_pestis_biovar_Medievalis_str._Harbin_35

7	AE017042	Yersinia_pestis_biovar_Microtus_str._91001
8	AL590842	Yersinia_pestis_CO92
9	CP001585	Yersinia_pestis_D106004
10	CP001589	Yersinia_pestis_D182038
11	AE009952	Yersinia_pestis_KIM10+
12	CP000305	Yersinia_pestis_Nepal516
13	CP000668	Yersinia_pestis_Pestoides_F
14	CP001593	Yersinia_pestis_Z176003
15	CP000720	Yersinia_pseudotuberculosis_IP_31758
16	CP001048	Yersinia_pseudotuberculosis_PB1/+
17	CP000950	Yersinia_pseudotuberculosis_YPIII
18	FN554766	Escherichia_coli_042
19	CP000247	Escherichia_coli_536
20	CU928145	Escherichia_coli_55989
21	CP001665	Escherichia_coli_'BL21-Gold(DE3)pLysS_AG'
22	CP001671	Escherichia_coli_ABU_83972
23	CP000468	Escherichia_coli_APEC_O1
24	CP004009	Escherichia_coli_APEC_O78
25	CP000946	Escherichia_coli_ATCC_8739
26	CP000819	Escherichia_coli_B_str._REL606
27	CP001396	Escherichia_coli_BW2952
28	AE014075	Escherichia_coli_CFT073
29	CP001637	Escherichia_coli_DH1
30	CP000800	Escherichia_coli_E24377A
31	CU928162	Escherichia_coli_ED1a
32	FN649414	Escherichia_coli_ETEC_H10407
33	CP000802	Escherichia_coli_HS
34	CU928160	Escherichia_coli_IAI1
35	CU928164	Escherichia_coli_IAI39
36	CP001969	Escherichia_coli_IHE3034
37	CP002516	Escherichia_coli_KO11
38	CP002970	Escherichia_coli_KO11FL
39	CP002797	Escherichia_coli_NA114
40	CP003034	Escherichia_coli_O7:K1_str._CE10
41	AP010953	Escherichia_coli_O26:H11_str._11368
42	CP001846	Escherichia_coli_O55:H7_str._CB9615
43	CP003109	Escherichia_coli_O55:H7_str._RM12579
44	CP001855	Escherichia_coli_O83:H1_str._NRG_857C
45	AP010958	Escherichia_coli_O103:H2_str._12009
46	CP003297	Escherichia_coli_O104:H4_str._2009EL-2050
47	CP003301	Escherichia_coli_O104:H4_str._2009EL-2071
48	CP003289	Escherichia_coli_O104:H4_str._2011C-3493
49	AP010960	Escherichia_coli_O111:H-_str._11128
50	FM180568	Escherichia_coli_0127:H6_E2348/69
51	CP001164	Escherichia_coli_O157:H7_str._EC4115
52	AE005174	Escherichia_coli_O157:H7_EDL933
53	BA000007	Escherichia_coli_O157:H7_str._Sakai
54	CP001368	Escherichia_coli_O157:H7_str._TW14359
55	CP002291	Escherichia_coli_P12b
56	CU928161	Escherichia_coli_S88
57	AP009240	Escherichia_coli_SE11
58	AP009378	Escherichia_coli_SE15
59	CP000970	Escherichia_coli_SMS-3-5

60	CP002211	Escherichia_coli_str._'clone_D_i2'
61	CP002212	Escherichia_coli_str._'clone_D_i14'
62	CP000948	Escherichia_coli_str._K12_substr._DH10B
63	AP012306	Escherichia_coli_str._K-12_substr._MDS42
64	U00096	Escherichia_coli_str._K-12_substr._MG1655
65	AP009048	Escherichia_coli_str._K12_substr._W3110
66	CP002167	Escherichia_coli_UM146
67	CU928163	Escherichia_coli_UMN026
68	CP002729	Escherichia_coli_UMNK88
69	CP000243	Escherichia_coli_UTI89
70	CP001925	Escherichia_coli_Xuzhou21
71	CP001063	Shigella_boydii_CDC_3083-94
72	CP000036	Shigella_boydii_Sb227
73	CP000034	Shigella_dysenteriae_Sd197
74	AE005674	Shigella_flexneri_2a_str._301
75	AE014073	Shigella_flexneri_2a_str._2457T
76	CP000266	Shigella_flexneri_5_str._8401
77	CP001383	Shigella_flexneri_2002017
78	HE616528	Shigella_sonnei_53G
79	CP000038	Shigella_sonnei_Ss046
80	CP006608	Salmonella_bongori_N268-08
81	FR877557	Salmonella_bongori_NCTC_12419
82	CP000880	Salmonella_enterica_subsp._arizonae_serovar
83	CP001138	Salmonella_enterica_subsp._enterica_serovar_Agona_str.
84	CP006053	Salmonella_enterica_subsp._enterica_serovar_Bareilly
85	AE017220	Salmonella_enterica_subsp._enterica_serovar_Choleraesuis
86	CP006055	Salmonella_enterica_subsp._enterica_Serovar_Cubana
87	CP001144	Salmonella_enterica_subsp._enterica_serovar_Dublin
88	AM933172	Salmonella_enterica_subsp._enterica_serovar_Enteritidis
89	AM933173	Salmonella_enterica_subsp._enterica_serovar_Gallinarum_str.
90	CP005390	Salmonella_enterica_subsp._enterica_Serovar_Heidelberg
91	CP004027	Salmonella_enterica_subsp._enterica_serovar_Javiana
92	CP001113	Salmonella_enterica_subsp._enterica_serovar_Newport_str.
93	CP006631	Salmonella_enterica_subsp._enterica_serovar_Newport
94	CP000026	Salmonella_enterica_subsp._enterica_serovar_Paratyphi_A_str.ATCC
95	FM200053	Salmonella_enterica_subsp._enterica_serovar_Paratyphi_A_str.
96	CP000886	Salmonella_enterica_subsp._enterica_serovar_Paratyphi_B_str.
97	CP000857	Salmonella_enterica_subsp._enterica_serovar_Paratyphi_C
98	CP006575	Salmonella_enterica_subsp._enterica_serovar_Pullorum_str.
99	CP001127	Salmonella_enterica_subsp._enterica_serovar_Schwarzengrund
100	AE014613	Salmonella_enterica_subsp._enterica_serovar_Typhi_Ty2
101	CP006048	Salmonella_enterica_subsp._enterica_serovar_Typhimurium_var.
102	CP003683	Klebsiella_oxytoca_E718
103	CP003218	Klebsiella_oxytoca_KCTC_1686
104	FO203501	Klebsiella_pneumoniae_subsp._rhinoscleromatis_strain
105	CP000964	Klebsiella_pneumoniae_342
106	CP002910	Klebsiella_pneumoniae_KCTC_2242
107	CP003785	Klebsiella_pneumoniae_subsp._pneumoniae_1084
108	CP003200	Klebsiella_pneumoniae_subsp._pneumoniae_HS11286
109	AP006725	Klebsiella_pneumoniae_subsp._pneumoniae_NTUH-K2044
110	CP001891	Klebsiella_variicola_At-22

Nodes:

Yersinia	1-17
E. coli	18-70
Shigella	71-79
Salmonella	80-101
Klebsiella	102-110

BA dataset:

1	NC_000117	Chlamydia trachomatis D/UW-3/CX
2	NC_000853	Thermotoga maritima MSB8
3	NC_000854	Aeropyrum pernix K1
4	NC_000868	Pyrococcus abyssi
5	NC_000907	Haemophilus influenzae Rd KW20
6	NC_000908	Mycoplasma genitalium G37
7	NC_000909	Methanocaldococcus jannaschii DSM 2661
8	NC_000911	Synechocystis sp. PCC 6803 DNA
9	NC_000912	Mycoplasma pneumoniae M129
10	NC_000913	Escherichia coli str. K-12 substr. MG1655
11	NC_000915	Helicobacter pylori 26695
12	NC_000916	Methanothermobacter thermautotrophicus str. Delta H
13	NC_000917	Archaeoglobus fulgidus DSM 4304
14	NC_000918	Aquifex aeolicus VF5
15	NC_000919	Treponema pallidum subsp. pallidum str. Nichols
16	NC_000921	Helicobacter pylori J99
17	NC_000922	Chlamydophila pneumoniae CWL029
18	NC_000961	Pyrococcus horikoshii OT3 DNA
19	NC_000962	Mycobacterium tuberculosis H37Rv
20	NC_000963	Rickettsia prowazekii str. Madrid E
21	NC_000964	Bacillus subtilis subsp. subtilis str. 168
22	NC_001263	Deinococcus radiodurans R1 chromosome 1
23	NC_001318	Borrelia burgdorferi B31
24	NC_002162	Ureaplasma parvum serovar 3 str. ATCC 700970
25	NC_002163	Campylobacter jejuni subsp. jejuni NCTC 11168 = ATCC 700819
26	NC_002179	Chlamydophila pneumoniae AR39
27	NC_002488	Xylella fastidiosa 9a5c
28	NC_002491	Chlamydophila pneumoniae J138 genomic DNA
29	NC_002505	Vibrio cholerae O1 biovar El Tor str. N16961 chromosome I
30	NC_002516	Pseudomonas aeruginosa PAO1
31	NC_002528	Buchnera aphidicola str. APS (Acyrthosiphon pisum)
32	NC_002570	Bacillus halodurans C-125 DNA
33	NC_002578	Thermoplasma acidophilum DSM 1728
34	NC_002607	Halobacterium sp. NRC-1
35	NC_002620	Chlamydia muridarum Nigg
36	NC_002655	Escherichia coli O157:H7 EDL933
37	NC_002662	Lactococcus lactis subsp. lactis Il1403
38	NC_002663	Pasteurella multocida subsp. multocida str. Pm70
39	NC_002677	Mycobacterium leprae TN

40	NC_002678	Mesorhizobium loti MAFF303099 DNA
41	NC_002689	Thermoplasma volcanium GSS1 DNA
42	NC_002695	Escherichia coli O157:H7 str. Sakai
43	NC_002696	Caulobacter crescentus CB15
44	NC_002737	Streptococcus pyogenes M1 GAS
45	NC_002745	Staphylococcus aureus subsp. aureus N315 DNA
46	NC_002754	Sulfolobus solfataricus P2
47	NC_002755	Mycobacterium tuberculosis CDC1551
48	NC_002758	Staphylococcus aureus subsp. aureus Mu50 DNA
49	NC_002771	Mycoplasma pulmonis UAB CTIP
50	NC_002927	Bordetella bronchiseptica strain RB50
51	NC_002928	Bordetella parapertussis strain 12822
52	NC_002929	Bordetella pertussis Tohama I
53	NC_002932	Chlorobium tepidum TLS
54	NC_002935	Corynebacterium diphtheriae NCTC 13129
55	NC_002940	Haemophilus ducreyi strain 35000HP
56	NC_002945	Mycobacterium bovis AF2122/97
57	NC_002947	Pseudomonas putida KT2440
58	NC_002950	Porphyromonas gingivalis W83
59	NC_002971	Coxiella burnetii RSA 493
60	NC_003028	Streptococcus pneumoniae TIGR4
61	NC_003030	Clostridium acetobutylicum ATCC 824
62	NC_003047	Sinorhizobium meliloti 1021
63	NC_003062	Agrobacterium fabrum str. C58 chromosome circular
64	NC_003098	Streptococcus pneumoniae R6
65	NC_003103	Rickettsia conorii str. Malish 7
66	NC_003106	Sulfolobus tokodaii str. 7
67	NC_003112	Neisseria meningitidis MC58
68	NC_003116	Neisseria meningitidis serogroup A strain Z2491
69	NC_003143	Yersinia pestis CO92
70	NC_003155	Streptomyces avermitilis MA-4680 = NBRC 14893 DNA
71	NC_003197	Salmonella enterica subsp. enterica serovar Typhimurium str. LT2
72	NC_003198	Salmonella enterica subsp. enterica serovar Typhi str. CT18
73	NC_003210	Listeria monocytogenes EGD-e
74	NC_003212	Listeria innocua Clip11262
75	NC_003272	Nostoc sp. PCC 7120 DNA
76	NC_003295	Ralstonia solanacearum GMI1000 chromosome
77	NC_003317	Brucella melitensis bv. 1 str. 16M chromosome I
78	NC_003361	Chlamydophila caviae GPIC
79	NC_003364	Pyrobaculum aerophilum str. IM2
80	NC_003366	Clostridium perfringens str. 13 DNA
81	NC_003413	Pyrococcus furiosus DSM 3638
82	NC_003450	Corynebacterium glutamicum ATCC 13032
83	NC_003454	Fusobacterium nucleatum subsp. nucleatum ATCC 25586
84	NC_003485	Streptococcus pyogenes MGAS8232
85	NC_003551	Methanopyrus kandleri AV19
86	NC_003552	Methanosarcina acetivorans str. C2A
87	NC_003869	Thermoanaerobacter tengcongensis MB4
88	NC_003888	Streptomyces coelicolor A3(2)
89	NC_003901	Methanosarcina mazei strain Goe1
90	NC_003902	Xanthomonas campestris pv. campestris str. ATCC 33913
91	NC_003919	Xanthomonas axonopodis pv. citri str. 306
92	NC_003923	Staphylococcus aureus subsp. aureus MW2 DNA

93	NC_003997	Bacillus anthracis str. Ames
94	NC_004061	Buchnera aphidicola str. Sg (Schizaphis graminum)
95	NC_004070	Streptococcus pyogenes MGAS315
96	NC_004088	Yersinia pestis KIM10+
97	NC_004113	Thermosynechococcus elongatus BP-1
98	NC_004116	Streptococcus agalactiae 2603V/R
99	NC_004193	Oceanobacillus iheyensis HTE831 DNA
100	NC_004307	Bifidobacterium longum NCC2705
101	NC_004310	Brucella suis 1330 chromosome I
102	NC_004337	Shigella flexneri 2a str. 301
103	NC_004342	Leptospira interrogans serovar Lai str. 56601 chromosome I
104	NC_004344	Wigglesworthia glossinidia endosymbiont of Glossina brevipalpis
105	NC_004347	Shewanella oneidensis MR-1
106	NC_004350	Streptococcus mutans UA159
107	NC_004368	Streptococcus agalactiae NEM316
108	NC_004369	Corynebacterium efficiens YS-314 DNA
109	NC_004431	Escherichia coli CFT073
110	NC_004432	Mycoplasma penetrans HF-2 DNA
111	NC_004459	Vibrio vulnificus CMCP6 chromosome I
112	NC_004461	Staphylococcus epidermidis ATCC 12228
113	NC_004463	Bradyrhizobium japonicum USDA 110
114	NC_004545	Buchnera aphidicola str. Bp (Baizongia pistaciae)
115	NC_004551	Tropheryma whipplei TW08/27
116	NC_004556	Xylella fastidiosa Temecula1
117	NC_004557	Clostridium tetani E88
118	NC_004567	Lactobacillus plantarum WCFS1
119	NC_004572	Tropheryma whipplei str. Twist
120	NC_004578	Pseudomonas syringae pv. tomato str. DC3000
121	NC_004603	Vibrio parahaemolyticus RIMD 2210633 chromosome 1
122	NC_004606	Streptococcus pyogenes SSI-1 DNA
123	NC_004631	Salmonella enterica subsp. enterica serovar Typhi Ty2
124	NC_004663	Bacteroides thetaiotaomicron VPI-5482
125	NC_004668	Enterococcus faecalis V583
126	NC_004722	Bacillus cereus ATCC 14579
127	NC_004741	Shigella flexneri 2a str. 2457T
128	NC_004757	Nitrosomonas europaea ATCC 19718
129	NC_004829	Mycoplasma gallisepticum str. R(low)
130	NC_004917	Helicobacter hepaticus ATCC 51449
131	NC_005027	Rhodopirellula baltica SH 1
132	NC_005042	Prochlorococcus marinus subsp. marinus str. CCMP1375
133	NC_005043	Chlamydophila pneumoniae TW-183
134	NC_005061	Blochmannia floridanus
135	NC_005070	Synechococcus sp. WH 8102
136	NC_005071	Prochlorococcus marinus MIT9313
137	NC_005072	Prochlorococcus marinus MED4
138	NC_005085	Chromobacterium violaceum ATCC 12472
139	NC_005090	Wolinella succinogenes DSM 1740
140	NC_005125	Gloeobacter violaceus PCC 7421
141	NC_005126	Photorhabdus luminescens subsp. laumondii TTO1
142	NC_005139	Vibrio vulnificus YJ016 DNA chromosome I
143	NC_005213	Nanoarchaeum equitans Kin4-M

Nodes at Phylum level:

Crenarchaeota: 3 46 66 79
Euryarchaeota: 4 7 12 13 18 33 34 41 81 85 86 89
Nanoarchaeota: 143
Aquificales: 14
Bacteroidetes: 58 124
Chlamydiales: 1 17 26 28 35 78 133
Chlorobi: 53
Cyanobacteria: 8 75 97 132 135 136 137 140
High G+C Firmicutes: 19 39 47 54 56 70 82 88 100 108 115 119
Low G+C Firmicutes: 6 9 21 24 32 37 44 45 48 49 60 61 64 73 74 80 83 84 87 92 93 95 98 99 106 107 110 112 117 118 122 125 126 129
Planctomycetes: 131
Proteobacteria: 5 10 11 16 20 25 27 29 30 31 36 38 40 42 43 50 51 52 55 57 59 62 63 65 67 68 69 71 72 76 77 90 91 94 96 101 102 104 105 109 111 113 114 116 120 121 123 127 128 130 134 138 139 141 142
Spirochaetales: 15 23 103
Thermotogales: 2
Thermus/Deinococcus group: 22

Nodes at Class level:

Crenarchaeota:		
	1	Aeropyrum: 3
	2	Sulfolobales: 46 66
	3	Thermoproteales: 79
Euryarchaeota:		
	4	Archaeoglobales: 13
	5	Halobacteriales: 34
	6	Methanobacteriales: 12
	7	Methanococcales: 7
	8	Methanopyrales: 85
	9	Methanosarcinales: 86 89
	10	Thermococcales: 4 18 81
	11	Thermoplasmales: 33 41
Nanoarchaeota:		
	12	Nanoarchaeum: 143
Aquificales:		
	13	Aquificaceae: 14
Bacteroidetes:		
	14	Bacteroidaceae: 124
	15	Porphyromonadaceae: 58
Chlamydiales:		
	16	Chlamydiaceae: 1 17 26 28 35 78 133
Chlorobi:		
	17	Chlorobiales: 53
Cyanobacteria:		
	18	Chroococcales: 8 97 135 140
	19	Nostocales: 75

	20	Prochlorophytes: 132 136 137
High G+C Firmicutes:		
	21	Actinomycetales: 19 39 47 54 56 70 82 88 100 108 115 119
Low G+C Firmicutes:		
	22	Bacillus/Clostridium group: 6 9 21 24 32 37 44 45 48 49 60 61 64 73 74 80 83 84 87 92 93 95 98 99 106 107 110 112 117 118 122 125 126 129
Planctomycetes:		
	23	Planctomycetales: 131
Proteobacteria:		
	24	alpha subdivision: 20 40 43 62 63 65 77 101 113
	25	beta subdivision: 50 51 52 67 68 76 128 138
	26	epsilon subdivision: 11 16 25 130 139
	27	gamma subdivision: 5 10 27 29 30 31 36 38 42 55 57 59 69 71 72 90 91 94 96 102 104 105 109 111 114 116 120 121 123 127 134 141 142
Spirochaetales:		
	28	Leptospiraceae: 103
	29	Spirochaetaceae: 15 23
Thermotogales:		
	30	Thermotoga: 2
Thermus/Deinococcus group:		
	31	Deinococcus: 22

BAC dataset:

1	CP003123	Bartonella_australis_Aust/NH1
2	CP000524	Bartonella_bacilliformis_KC583
3	CP001562	Bartonella_grahamii_as4aup
4	BX897699	Bartonella_henselae_strain_Houston-1
5	BX897700	Bartonella_quintana_str._Toulouse
6	AM260525	Bartonella_tribocorum_CIP_105476
7	AP011121	Acetobacter_pasteurianus_IFO_3283-01
8	AP011163	Acetobacter_pasteurianus_IFO_3283-01-42C
9	AP011128	Acetobacter_pasteurianus_IFO_3283-03
10	AP011135	Acetobacter_pasteurianus_IFO_3283-07
11	AP011170	Acetobacter_pasteurianus_IFO_3283-12
12	AP011142	Acetobacter_pasteurianus_IFO_3283-22
13	CP001612	Rickettsia_africae_ESF-5
14	CP000847	Rickettsia_akari_str._Hartford
15	CP000087	Rickettsia_bellii_RML369-C
16	CP000409	Rickettsia_canadensis_str._McKiel
17	AE006914	Rickettsia_conorii_str._Malish_7
18	CP000053	Rickettsia_felis_URRWXCal2
19	CP000507	Shewanella_amazonensis_SB2B
20	CP002767	Shewanella_baltica_BA175
21	CP002811	Shewanella_baltica_OS117
22	CP000563	Shewanella_baltica_OS155
23	CP000753	Shewanella_baltica_OS185
24	CP000891	Shewanella_baltica_OS195

25	FN554766	Escherichia_coli_042
26	CP000247	Escherichia_coli_536
27	CU928145	Escherichia_coli_55989
28	CP001665	Escherichia_coli_'BL21-Gold(DE3)pLysS_AG'
29	CP001671	Escherichia_coli_ABU_83972
30	CP000468	Escherichia_coli_APEC_O1
31	CP001828	Legionella_pneumophila_2300/99_Alcoy
32	CP000675	Legionella_pneumophila_str._Corby
33	CR628337	Legionella_pneumophila_str._Lens
34	CR628336	Legionella_pneumophila_str._Paris
35	AE017354	Legionella_pneumophila_subsp._pneumophila_str._Philadelphia
36	CP003730	Legionella_pneumophila_subsp._pneumophila_str._Thunder
37	AE017143	Haemophilus_ducreyi_strain_35000HP
38	CP000057	Haemophilus_influenzae_86-028NP
39	CP000671	Haemophilus_influenzae_PittEE
40	CP000672	Haemophilus_influenzae_PittGG
41	CP002276	Haemophilus_influenzae_R2846
42	CP002277	Haemophilus_influenzae_R2866
43	CP001921	Acinetobacter_baumannii_1656-2
44	CP001182	Acinetobacter_baumannii_AB0057
45	CP001172	Acinetobacter_baumannii_AB307-0294
46	CP000863	Acinetobacter_baumannii_ACICU
47	CP000521	Acinetobacter_baumannii_ATCC_17978
48	CU468230	Acinetobacter_baumannii_SDF
49	CP003402	Francisella_noatunensis_subsp._orientalis_str._Toba_04
50	CP000439	Francisella_tularensis_subsp._novicida_U112
51	CP003862	Francisella_tularensis_subsp._holarctica_FSC200
52	CP000803	Francisella_tularensis_subsp._holarctica_FTNF002-00
53	AM233362	Francisella_tularensis_subsp._holarctica_LVS
54	CP000437	Francisella_tularensis_subsp._holarctica_OSU18
55	CP000050	Xanthomonas_campestris_pv._campestris_str._8004
56	AE008922	Xanthomonas_campestris_pv._campestris_str._ATCC_33913
57	AE008923	Xanthomonas_axonopodis_pv._citri_str._306
58	CP003778	Xanthomonas_citri_subsp._citri_Aw12879
59	AM039952	Xanthomonas_campestris_pv._vesicatoria
60	AE013598	Xanthomonas_oryzae_pv._oryzae_KACC_10331
61	CP001124	Geobacter_bemidjiensis_Bem
62	CP001390	Geobacter_daltonii_FRC-32
63	CP001089	Geobacter_lovleyi_SZ
64	CP000148	Geobacter_metallireducens_GS-15
65	CP002479	Geobacter_sp._M18
66	CP001661	Geobacter_sp._M21
67	AM260522	Helicobacter_acinonychis_str._Sheeba
68	AE017125	Helicobacter_hepaticus_ATCC_51449
69	FN555004	Helicobacter_mustelae_12198
70	CP000012	Helicobacter_pylori_51
71	CP001680	Helicobacter_pylori_52
72	FN598874	Helicobacter_pylori_B8
73	FN597644	Bacillus_amyloliquefaciens_DSM7
74	CP000560	Bacillus_amyloliquefaciens_FZB42
75	AE017334	Bacillus_anthracis_str._'Ames_Ancestor'
76	AE016879	Bacillus_anthracis_str._Ames
77	CP002091	Bacillus_anthracis_str._H9401

78	CP002394	Bacillus_cellulosilyticus_DSM_2522
79	AE001437	Clostridium_acetobutylicum_ATCC_824
80	CP003326	Clostridium_acidurici_9a
81	CP001581	Clostridium_botulinum_A2_str._Kyoto
82	CP000962	Clostridium_botulinum_A3_str._Loch_Maree
83	AM412317	Clostridium_botulinum_A_str._ATCC_3502
84	CP000726	Clostridium_botulinum_A_str._ATCC_19397
85	AE009948	Streptococcus_agalactiae_2603V/R
86	CP000114	Streptococcus_agalactiae_A909
87	CP002215	Streptococcus_dysgalactiae_subsp._equisimilis_ATCC_12394
88	AP010935	Streptococcus_dysgalactiae_subsp._equisimilis_GGS_124
89	FM204883	Streptococcus_equi_subsp._equi_4047
90	FM204884	Streptococcus_equi_subsp._zooepidemicus_H70
91	CP001393	Caldicellulosiruptor_bescii_DSM_6725
92	CP002219	Caldicellulosiruptor_hydrothermalis_108
93	CP002326	Caldicellulosiruptor_kristjanssonii_177R1B
94	CP002164	Caldicellulosiruptor_obsidiansis_OB47
95	CP002216	Caldicellulosiruptor_owensensis_OL
96	CP000679	Caldicellulosiruptor_saccharolyticus_DSM_8903
97	CP003699	Mycobacterium_massiliense_str._GO_06
98	CP000479	Mycobacterium_avium_104
99	AE016958	Mycobacterium_avium_subsp._paratuberculosis_str._k10
100	CP002095	Mycobacterium_bovis_BCG_str._Mexico
101	AM408590	Mycobacterium_bovis_BCG_Pasteur_1173P2
102	AP010918	Mycobacterium_bovis_BCG_str._Tokyo_172
103	AP009256	Bifidobacterium_adolescentis_ATCC_15703
104	CP002567	Bifidobacterium_animalis_subsp._animalis_ATCC_25527
105	CP001213	Bifidobacterium_animalis_subsp._lactis_AD011
106	CP003497	Bifidobacterium_animalis_subsp._lactis_B420
107	CP001853	Bifidobacterium_animalis_subsp._lactis_BB-12
108	CP003498	Bifidobacterium_animalis_subsp._lactis_Bi-07
109	CP001841	Treponema_azotonutricium_ZAS-9
110	AE017226	Treponema_denticola_ATCC_35405
111	CP001752	Treponema_pallidum_subsp._pallidum_str._Chicago
112	CP003064	Treponema_pallidum_subsp._pallidum_str._Mexico_A
113	CP002103	Treponema_paraluiscuniculi_Cuniculi_A
114	CP001843	Treponema_primitia_ZAS-2
115	AE002160	Chlamydia_muridarum_Nigg
116	AM884176	Chlamydia_trachomatis_strain_L2/434/Bu
117	CP000051	Chlamydia_trachomatis_A/HAR-13
118	FM872308	Chlamydia_trachomatis_B/Jali20/OT
119	FM872307	Chlamydia_trachomatis_B/TZ1A828/OT
120	CP002052	Chlamydia_trachomatis_D-EC
121	AE006470	Chlorobium_tepidum_TLS
122	CP000108	Chlorobium_chlorochromatii_CaD3
123	CP001097	Chlorobium_limicola_DSM_245
124	CP001101	Chlorobium_phaeobacteroides_BS1
125	CP000492	Chlorobium_phaeobacteroides_DSM_266
126	CP000607	Chlorobium_phaeovibrioides_DSM_265
127	CP001047	Mycoplasma_arthritidis_158L3-1
128	CP002188	Mycoplasma_bovis_PG45_clone_MU_clone_A2
129	CP000123	Mycoplasma_capricolum_subsp._capricolum_ATCC_27343
130	FM864216	Mycoplasma_conjunctivae_HRC/581T

131	CP001991	Mycoplasma_crocodyli_MP145
132	CP001995	Mycoplasma_fermentans_JER
133	CP000551	Prochlorococcus_marinus_str._AS9601
134	CP000878	Prochlorococcus_marinus_str._MIT_9211
135	CP000825	Prochlorococcus_marinus_str._MIT_9215
136	CP000576	Prochlorococcus_marinus_str._MIT_9301
137	CP000554	Prochlorococcus_marinus_str._MIT_9303
138	CP000111	Prochlorococcus_marinus_str._MIT_9312
139	AE004969	Neisseria_gonorrhoeae_FA_1090
140	CP003909	Neisseria_gonorrhoeae_MS11
141	CP001050	Neisseria_gonorrhoeae_NCCP11945
142	FN995097	Neisseria_lactamica_020-06
143	CP000381	Neisseria_meningitidis_053442
144	AM421808	Neisseria_meningitidis_serogroup_C_FAM18

Nodes at Order level:

1-6	Rhizobiales
7-12	Rhodospirallales
13-18	Rickettiales
19-24	Shewanella
25-30	Enterobacteriales
31-36	Legionellales
37-42	Pasteurellales
43-48	Pseudomonadales
49-54	Thiotrichales
55-60	Xanthomonadales
61-66	Desulfuromonadales
67-72	Campylobacteriales
73-78	Bacillales
79-84	Clostridiales
85-90	Lactobacillales
91-96	Thermoanaerobacteriales
97-102	Actinomycetales
103-108	Bifidobacteriales
109-114	Spirochaetales
115-120	Chlamydiales
121-126	Chlorobiales
127-132	Mycoplasmatales
133-138	Synechococcales
139-144	Neisseriales
